# Comparative Genomics of a Plant-Pathogenic Fungus, *Pyrenophora tritici-repentis*, Reveals Transduplication and the Impact of Repeat Elements on Pathogenicity and Population Divergence

**DOI:** 10.1534/g3.112.004044

**Published:** 2013-01-01

**Authors:** Viola A. Manning, Iovanna Pandelova, Braham Dhillon, Larry J. Wilhelm, Stephen B. Goodwin, Aaron M. Berlin, Melania Figueroa, Michael Freitag, James K. Hane, Bernard Henrissat, Wade H. Holman, Chinnappa D. Kodira, Joel Martin, Richard P. Oliver, Barbara Robbertse, Wendy Schackwitz, David C. Schwartz, Joseph W. Spatafora, B. Gillian Turgeon, Chandri Yandava, Sarah Young, Shiguo Zhou, Qiandong Zeng, Igor V. Grigoriev, Li-Jun Ma, Lynda M. Ciuffetti

**Affiliations:** * Department of Botany and Plant Pathology, Oregon State University, Corvallis, Oregon 97331; † Department of Forest Sciences, University of British Columbia, Vancouver, British Columbia, Canada, V6T 1Z4; ‡ Carbone/Ferguson Laboratories, Division of Neuroscience, Oregon National Primate Research Center (ONPRC), Beaverton, Oregon 97006; § USDA–Agricultural Research Service, Purdue University, West Lafayette, Indiana 47907; ** The Broad Institute, Cambridge, Massachusetts 02142; †† USDA-Agricultural Research Service, Forage Seed and Cereal Research Unit, Oregon State University, Corvallis, Oregon 97331; ‡‡ Department of Biochemistry and Biophysics, Oregon State University, Corvallis, Oregon 97331; §§ Center for Genome Research and Biocomputing, Oregon State University, Corvallis, Oregon 97331; *** Commonwealth Scientific and Industrial Research Organization−Plant Industry, Centre for Environment and Life Sciences, Floreat, Western Australia 6014, Australia; †††† Architecture et Fonction des Macromolécules Biologiques, Aix-Marseille Université, Centre National de la Recherche Scientifique, 13288 Marseille cedex 9, France; ‡‡‡ Roche 454, Branford, Connecticut 06405; §§§ US DOE Joint Genome Institute, Walnut Creek, California 94598; **** Australian Centre for Necrotrophic Fungal Pathogens, Department of Environment and Agriculture, Curtin University, Bentley, Western Australia 6845, Australia; †††† National Center for Biotechnology Information, National Library of Medicine, National Institutes of Health, Department of Health and Human Services, Bethesda, Maryland 20894; ‡‡‡‡ Laboratory for Molecular and Computational Genomics, Department of Chemistry, Laboratory of Genetics, UW Biotechnology Center, University of Wisconsin–Madison, Madison, Wisconsin 53706; §§§§ Department of Plant Pathology and Plant-Microbe Biology, Cornell University, Ithaca, New York 14850; ***** Department of Biochemistry and Molecular Biology, University of Massachusetts, Amherst, Massachusetts 01003

**Keywords:** wheat (*Triticum aestivum*), copy number variation, histone H3 transduplication, ToxA, ToxB, anastomosis

## Abstract

*Pyrenophora tritici-repentis* is a necrotrophic fungus causal to the disease tan spot of wheat, whose contribution to crop loss has increased significantly during the last few decades. Pathogenicity by this fungus is attributed to the production of host-selective toxins (HST), which are recognized by their host in a genotype-specific manner. To better understand the mechanisms that have led to the increase in disease incidence related to this pathogen, we sequenced the genomes of three *P. tritici-repentis* isolates. A pathogenic isolate that produces two known HSTs was used to assemble a reference nuclear genome of approximately 40 Mb composed of 11 chromosomes that encode 12,141 predicted genes. Comparison of the reference genome with those of a pathogenic isolate that produces a third HST, and a nonpathogenic isolate, showed the nonpathogen genome to be more diverged than those of the two pathogens. Examination of gene-coding regions has provided candidate pathogen-specific proteins and revealed gene families that may play a role in a necrotrophic lifestyle. Analysis of transposable elements suggests that their presence in the genome of pathogenic isolates contributes to the creation of novel genes, effector diversification, possible horizontal gene transfer events, identified copy number variation, and the first example of transduplication by DNA transposable elements in fungi. Overall, comparative analysis of these genomes provides evidence that pathogenicity in this species arose through an influx of transposable elements, which created a genetically flexible landscape that can easily respond to environmental changes.

Changes in global and regional climates, the adoption of alternative agricultural practices, and the global exchange of commodities often lead to changes in the profiles and ranges of pathogens that affect our most important agricultural food crops (Bailey 1996; Gange *et al.* 2011; Pautasso *et al.* 2012; Rees and Platz 1992; Stukenbrock and McDonald 2008). The disease tan spot of wheat, caused by the necrotrophic fungal pathogen *Pyrenophora tritici-repentis* (Died.) Drechs. (syn. *P. trichostoma* (Fr.) Fckl.), anamorph: *Helminthosporium tritici-repentis* (syn. *Drechslera tritici-repentis* (Died.) Shoem. (Ciuffetti and Tuori 1999, De Wolf *et al.* 1998, Strelkov and Lamari 2003), was first detected in the United States in the 1940s (Barrus 1942). Within a few decades, it was recognized as one of the fastest-spreading and economically important foliar diseases in major wheat-growing regions worldwide (Benslimane *et al.* 2011; Kohli *et al.* 1992; Murray and Brennan 2009; Postnikova and Khasanov 1998; Rees and Platz, 1992). The increase in tan spot often has been attributed to shifts in agricultural practices that allow pathogen accumulation in the field, which provides abundant inoculum to initiate disease cycles (Bailey 1996; Bockus and Claasen 1992; Bockus and Shroyer 1998; Brennan and Murray 1988; Ciuffetti *et al.* 1998; De Wolf *et al.* 1998; Rees and Platz, 1983; Summerall *et al.* 1988; Sutton and Vyn 1990). Most recently, it has been proposed that the increase in disease was due not only to changes in agricultural practices but also to the acquisition of a potent virulence/pathogenicity factor, *ToxA*, by horizontal gene transfer (HGT) (Friesen *et al.* 2006).

Symptoms produced by *P. tritici-repentis* (*Ptr*) are the result of host-selective toxins (HST) on toxin-sensitive cultivars (Ciuffetti *et al.* 2010; Lamari and Strelkov 2010; Strelkov and Lamari 2003). Currently there are three HSTs that have been described in the *Ptr*-wheat pathosystem, although others remain to be characterized (Ali *et al.* 2009; Andrie *et al.* 2007; Lepoint *et al.* 2010; Tuori *et al.* 1995). Unlike the majority of HSTs produced by fungi in the Pleosporales that are secondary metabolites (Markham and Hille 2001, Wolpert *et al.* 2002), at least two of the HSTs of *Ptr* are proteinaceous: Ptr ToxA (ToxA) and Ptr ToxB (ToxB) (Ballance *et al.* 1989; Ciuffetti *et al.* 1998; Martinez *et al.* 2001; Strelkov *et al.* 1999; Tomas *et al.* 1990; Tuori *et al.* 1995). Partial characterization of Ptr ToxC (ToxC) indicates that it is a low-molecular-weight, polar, nonionic compound (Effertz *et al.* 2002). These HSTs may be present singly or in combination in a given isolate and the composition of these HSTs (or lack of a HST) determines the eight currently characterized races of *Ptr* (Lamari *et al.* 2003; Strelkov and Lamari 2003). ToxA is the most common HST in field isolates (Antoni *et al.* 2009; Ali and Francl 2002; Ali *et al.* 2009; Ballance *et al.* 1989; Benslimane *et al.* 2011; Friesen *et al.* 2005; Lamari *et al.* 2005; Leisova *et al.* 2008; Lepoint *et al.* 2010). The discovery of a highly similar *ToxA* gene in the wheat pathogen *Stagonospora nodorum* led to the hypothesis of a HGT event of *ToxA* from *S. nodorum* to *Ptr* shortly before 1941 (Friesen *et al.* 2006). However, the presence of *ToxA* in all possible combinations with other HSTs produced by *Ptr* (Lamari *et al.* 2003), and the report of *ToxA* in the closely related barley pathogen *P. teres* (Leisova-Svobodova *et al.*, 2010a,b), though currently unable to confirm (L. Ciuffetti laboratory and B. McDonald laboratory, personal communications), suggests that the evolutionary history of ToxA may be more complicated than previously believed.

*Ptr* is a homothallic fungus (Lepoint *et al.* 2010), yet populations appear to be genetically diverse and isolates can have variable numbers and sizes of chromosomes (Aboukhaddour *et al.* 2009, 2011; Friesen *et al.* 2005; Leisova *et al.* 2008; Lepoint *et al.* 2010; Lichter *et al.* 2002; Moreno *et al.* 2008; Singh and Hughes 2006). Studies on population genetics have failed to find genetic groupings based on race or geographic location, with a few notable exceptions. Some genetic markers can be correlated with *ToxA*-containing isolates (Leisova-Svobodova *et al.* 2010b; Lichter *et al.* 2002), which appear to be more genetically similar to each other than they are to *ToxA*-minus isolates (Aboukhaddour *et al.* 2011). Other studies have shown that there are differences between isolates that can cause disease on wheat and those that do not, other than the presence (or absence) of HSTs [for simplicity, we refer to those isolates that do not cause disease on wheat as non-pathogenic (Aboukhaddour *et al.* 2009, 2011; Cao *et al.* 2009; Leisova-Svobodova *et al.* 2010b; Lepoint *et al.* 2010; Lichter *et al.* 2002)]. These differences include larger genome sizes for those isolates that cause disease (Aboukhaddour *et al.* 2009; Lichter *et al.* 2002; Martinez *et al.* 2004) and a tendency of nonpathogenic isolates to group together (although these groups are not exclusive of pathogenic isolates) in analyses of molecular data obtained by IRAP [*i.e.*, interretrotransposon amplified polymorphism (Leisova-Svobodova *et al.* 2010b)] analysis and *MAT* locus sequencing (Lepoint *et al.* 2010).

Genetic analyses have established a strong correlation between toxin production by *Ptr* and host sensitivity to the toxin and disease susceptibility to the pathogen, which underscores the “inverse” gene-for-gene nature of the *Ptr*-wheat pathosystem. In tan spot of wheat, virulence of the pathogen and disease susceptibility of the host appear to be the dominant factors (Effertz *et al.* 2002; Friesen and Faris 2004; Gamba *et al.* 1998; Lamari and Bernier 1991; Strelkov and Lamari 2003; Wolpert *et al.* 2002). Consequently, just as investigations of “classical” gene-for-gene interactions contributed much to our understanding of pathogen avirulence and host resistance (incompatibility), those relating to tan spot of wheat have considerable potential to contribute to our understanding of pathogen virulence and host susceptibility (compatibility). Indeed, understanding the *Ptr* pathosystem as an inverse gene-for-gene system has already led to insights into the contribution of HSTs and other effectors to a necrotrophic lifestyle (Friesen and Faris 2010) in this and other necrotrophic pathogenic fungi.

In this study, we generated a high-quality assembly and annotated reference genome of a pathogenic isolate (BFP-ToxAC) of *P. tritici-repentis*. To determine whether genome structure contributes to pathogenicity, the genomes of two additional isolates, one pathogenic (DW7-ToxB) and one nonpathogenic on wheat (SD20-NP) were sequenced and a comparative genome analysis was conducted. In addition, sequences of transcripts produced by the reference isolate (BFP-ToxAC), the sequenced nonpathogenic isolate (SD20-NP), and an additional pathogenic isolate (SO3-P) were analyzed. We found that transposable elements play a significant role in shaping the genomic landscape of pathogenic isolates. Analyses also led to the discovery that fungi, like plants, contain transduplicating DNA transposons, capable of replicating nontransposable element domains and coding regions throughout the genome. The acquisition of genetic flexibility and its role in pathogenicity as well as the identification of pathogen-specific genes will be discussed.

## Materials and Methods

### Phylogenetic analysis

Phylogenetic analyses were performed using the phylogenetic pipeline, Hal (Robbertse *et al.* 2006, 2011). To summarize in brief, orthologous clusters of proteins were estimated from 16 *Ascomycota* genomes using the program MCL (Enright *et al.* 2002) across 13 inflation parameters (1.1, 1.2, 1.3, 1.4, 1.5, 1.7, 2.0, 2.5, 3.0, 3.5, 4.0, 4.5, and 5.0). Orthologous clusters were then filtered to remove redundant clusters, clusters with more than one protein per genome, and clusters with fewer than 15 genomes per cluster. This resulted in 2319 orthologous clusters that were individually aligned using the program MUSCLE (Edgar 2004), with poorly aligned regions masked using GBlocks (Castresana 2000). The best model of amino acid substitution (Abascal *et al.* 2005) was estimated for the 100 longest post-GBlocks protein alignments. These 100-protein alignments were concatenated into a single super alignment, which contained 93,210 amino acid positions, and analyzed using RAxML (Stamatakis 2006) with the best model of amino acid substitution assigned to each protein partition; branch support was estimated from 100 bootstrap partitions with the rapid bootstrap option in RAxML.

### Genomic DNA isolation, reference genome sequencing, assembly, and optical mapping

Genomic DNA from the three wheat-pathogenic isolates Pt-1C (BFP-ToxAC), SO3 (SO3-P), and DW7 (DW7-ToxB) and the nonpathogenic isolate SD20 (SD20-NP) of *Ptr* (Supporting Information, Table S1) was purified via organic extraction in conjunction with a modified polysaccharide precipitation method (Martinez *et al.* 2004). For optical mapping, protoplasts were isolated as described previously (Andrie *et al.* 2007).

Whole-genome shotgun assemblies of BFP-ToxAC (6.93 X) were generated with Sanger technology. To compensate for the lack of genetic mapping information, a whole-genome optical map of the sequenced *Ptr* isolate was constructed using the optical mapping system (Dimalanta *et al.* 2004; Lin *et al.* 1999; Zhou *et al.* 2007, 2009) and the restriction enzyme *Afl*II in the Laboratory for Molecular and Computational Genomics, University of Wisconsin, Madison. The assembled sequence scaffolds were ordered and oriented, and the gaps were estimated based on the alignment of the assembled sequence scaffolds to the optical maps. The optical linkage group maps can be accessed at:

http://www.broadinstitute.org/annotation/genome/pyrenophora_tritici_repentis.3/MapsIndex.html. Genome sequence information is downloadable from: http://www.broadinstitute.org/annotation/genome/pyrenophora_tritici_repentis

### EST library preparation, mapping, and gene annotation

Gene annotation of the reference genome was facilitated by sequences from four EST libraries: three normalized libraries of transcripts produced in liquid culture by BFP-ToxAC, SD20-NP, and an additional pathogenic isolate, SO3-P, which produces an uncharacterized toxin; and one subtracted and normalized library from BFP-ToxAC *in planta*. Conidia were produced as described previously (Andrie *et al.* 2007) and used for both liquid culture and plant inoculations. For the libraries from liquid media, tissue from four culture conditions was harvested for RNA extraction: mycelial tissue from conidial inoculations of 1/4 potato dextrose broth (Difco, Becton, Dickinson, and Company, Sparks, MD) or modified Fries medium (Tomas and Bockus 1987) incubated for 48 hr at 25° in constant dark or constant light. For the *in planta* library, conidial inoculations were performed as described previously (Andrie *et al.* 2007). Plants were grown in a growth chamber set to 16 hr of light at 22° and 8 hr of darkness at 18°. Leaves were harvested 48 hr after inoculation and frozen in liquid nitrogen. All samples were stored at −80°.

RNA was isolated separately from each condition with the RNeasy Plant Mini Kit plus on-Column DNase Digestion with the RNase-Free DNase Set following the manufacturer’s instructions (QIAGEN, Chatsworth, CA). RNA integrity was assayed using the RNA 6000 nano LabChip kit on the Agilent Bioanalyzer 2100 (Agilent Technologies, Inc., Palo Alto, CA) at the Central Services Lab, Center for Genome Research and Biocomputing at Oregon State University, and its quantity was determined by a Nanodrop spectrophotometer. For each library from liquid culture, equal amounts of RNA (1 µg) from the four conditions were pooled, and cDNA was synthesized with a Mint cDNA synthesis kit (Evrogen, Moscow, Russia) and normalized with a TRIMMER cDNA normalization kit (Evrogen). For the *in planta* library, total RNA was isolated from mock-inoculated (driver) and BFP-ToxAC-inoculated (target) wheat leaves, mRNA was isolated using Dynabeads oligo (dT)_25_ (Invitrogen, Grand Island, NY), and subtraction was performed as described by Lönneborg *et al.* 1995. Subsequent cDNA synthesis and normalization of subtracted mRNA were performed as described previously. All cDNA libraries were cloned into the pGEM-T Easy vector (Promega, Madison, WI) and transformed into ElectroMAX DH10B T1 Phage Resistant Cells (Invitrogen) per the manufacturer’s instructions. Forward and reverse sequences of 5000 plasmids from each library were obtained and aligned to the genome using BLAT (Kent 2002). EST alignments with 90% identity over 50% of the EST length with canonical splice junctions were considered valid EST alignments suitable for building gene models.

Protein-encoding genes were annotated using a combination of manual curation, EST alignments, and *ab initio* gene predictions made by FGENESH, FGENESH+ (http://linux1.softberry.com) and GENEID (http://genome.crg.es/software/geneid). In addition, protein-encoding genes were predicted based on BLAST searches (E < 1e-10) of known genes available in public databases. HMMER searches also were performed using the Pfam library (Finn *et al.* 2010) to find Pfam domains on predicted protein sequences.

### Illumina sequencing

Genomic DNA of isolates DW7-ToxB and SD20-NP was randomly sheared into ~200-bp fragments using Covaris E210 (Covaris, Inc, Woburn, MA) according to the manufacturer’s recommendations and the resulting fragments were used to create an Illumina library. This library was sequenced on Illumina GAII (Illumina, Inc., San Diego, CA) sequencers that generated 75-bp paired-end reads. These reads were aligned to the reference genome and putative SNPs and small indels were called with maq-0.7.1 (Li *et al.* 2008) at default settings. K-mer analysis was performed with Tallymer (Kurtz *et al.* 2008).

To determine the presence/absence of features in the Illumina-sequenced genomes compared with the reference, read mapping was performed with SOAP2 (Li *et al.* 2009) using different mapping stringency criteria. The least stringent allowed reads with up to four mismatches and repeats to be mapped; the mid-stringency allowed mapping of reads that contain up to four mismatches but no repeat mapping; and the highest stringency allowed mapping of only those reads with exact matches. For each feature, data were normalized to reads per kilobase per million input reads and a range around the median from each stringency mapping was used to determine single coverage for that stringency. When determining the presence or absence of a repeat feature, we only used the least-stringent mapping criteria. To determine the presence or absence of a putative gene, all stringencies were considered. *De novo* assemblies were performed in Velvet (Zerbino and Birney 2008). Absence of a particular feature was confirmed by BLAST (E < 1e-100 or < 1e-50 for SSP) to the *de novo* assembled contigs.

### Comparative analysis of Pfam domains of selected cereal pathogens

Protein FASTA files were downloaded from the genome sequences of: *Pyrenophora teres* - NCBI (Ellwood *et al.* 2010); *Stagonospora nodorum* - Broad Institute [Annotation Update 2011-05-06 (Hane *et al.* 2007)]; *Mycosphaerella graminicola* - Joint Genome Institute (Goodwin *et al.* 2011); *Colletotrichum (Glomerella) graminicola* - Broad Institute; *Fusarium graminearum* - Broad Institute (Cuomo *et al.* 2007); and *Puccinia graminis* - Broad Institute (Duplessis *et al.* 2011). A stringent Pfam domain prediction (E < 1e-20) was performed for each organism with HMMER3 (Finn *et al.* 2011). Pfam domains were placed into categories relevant to plant pathogenesis and heat maps representing the number of each Pfam present in each genome were generated.

### Discovery and annotation of repetitive sequences and transposable elements

Initial detection of repeat sequences was performed at the Broad Institute with CrossMatch (http://www.genome.washington.edu/UWGC/analysistools/Swat.cfm). The genome sequence was searched against itself, filtering for alignments longer than 200 bp with greater than 60% sequence similarity. Full-length transposable elements were annotated using a combination of computational predictions based on BLAST analysis for transposase genes, and manual inspection.

For the *de novo* repeat analysis, repetitive elements were identified using RECON (Bao and Eddy 2002). Output was parsed to include families with 10 or more copies. Annotation of these families was based on BLAST analysis done with *blastx* against the NCBI ‘nr’ database. When in doubt, based on the protein frame in *blastx*, *blastp* was used. Inverted repeats were identified using ‘einverted’ in the EMBOSS package (Rice *et al.* 2000). Sequences from individual families were aligned using MUSCLE (Edgar 2004) and each alignment was edited manually. Sequences identified by RECON constitute the custom repeat library. This library accounted for 13.7% of the genome’s repetitive fraction. The custom library was used to mask the genome with RepeatMasker (http://www.repeatmasker.org). A total of 16.7% of the genome was found to be repetitive. This estimation of repeat content may be a slight overestimation (by 0.8%) due to an overlap between different repeats or nested insertions. Some additional annotations were performed in Censor (Jurka 2000). Repeat-induced point mutation (RIP) analysis was performed on the manual alignment files with RIPCAL (Hane and Oliver 2008). Only those families that were >700 bp (98 families) were considered as candidates for RIP.

### Gene family annotation

All pairwise comparisons for identity were performed in Needle (Needleman and Wunsch 1970). NRPS/PKS prediction: NRPSs were predicted by searching the genome for Pfam domains that correspond to the conserved AMP-binding enzyme, condensation domain, and phosphopantetheine attachment sites. For PTRG_10433, which contained two gaps, several attempts to fill the first gap in the sequence were not successful, but the second gap was filled in by polymerase chain reaction (PCR) amplification using primers NRPSF2 (5′-AACAGCCGGAGAGAACACAT-3′) and NRPSR2 (5′-AGTCCTGCAGCTCTGACTTG-3′) in a 50-µL reaction that contained 1.25 mM MgCl_2_, 0.0025 mM each dNTP, 2 mM primers, 10 ng of BFP-ToxAC genomic DNA, 0.25 µL of GoTaq of DNA polymerase, and 1X GoTaq Flexi buffer (Promega). Cycling was performed in a Mastercycler gradient machine (Eppendorf) with the following parameters: 94° for 5 min, 30 cycles of 94° for 45 sec, 58° for 45 sec, 72° for 1 min 30 sec, followed by 1 cycle at 72° for 7 min and a 4° hold. The PCR product was cloned into pGEM-T Easy vector and sequenced at the Central Services Lab in the Center for Genome Research and Biocomputing, Oregon State University, Corvallis, OR. PKSs were predicted by searching for Pfam domains that correspond to the conserved acyl transferase domain, beta-ketoacyl synthase N-terminal domains, and phosphopantetheine attachments sites. Coding regions were annotated manually. Putative clusters were predicted by manual inspection of the surrounding coding regions and, where possible, by comparisons with regions containing the homologous NRPS/PKS in the context of their genomes and known clusters.

CAZymes: Carbohydrate-active enzymes were identified by searching against a library of catalytic and carbohydrate-binding modules of carbohydrate-active enzymes [(Cantarel *et al.* 2009); see also www.cazy.org] using *blastp*. Additional data for comparison was adapted from Amselem *et al.* 2011.

Secreted protein prediction: Our initial dataset pooled proteins predicted to have a signal peptide [SignalP 3.0 (Dyrløv Bendtsen *et al.* 2004)] with those predicted to have extracellular localization [WoLF PSORT (Horton *et al.* 2007)]. We then looked for the presence of transmembrane domains (TM) in this dataset (TMHMM, http://www.cbs.dtu.dk/services/TMHMM/), and removed all proteins that had >1 TM. Because it is sometimes difficult to differentiate TMs from signal peptides, those proteins that were predicted to have one TM that began before aa 10 were retained in the data set. Conserved domains within these proteins were predicted with a HMMER search of the Pfam-A library with a cutoff of E < 1e-0.1 (Finn *et al.* 2010) and proteins were annotated with GO terms in Blast2GO (Conesa *et al.* 2005).

### Conidial anastomosis and microscopy

Conidia production was induced as described previously (Andrie *et al.* 2007). To test for conidial anastomosis, conidia were harvested 17 to 24 hr after induction, placed on a slide, and examined using light or fluorescent microscopy. Of 22 *Ptr* isolates tested for the presence of conidial anastomosis, 19 were positive (data not shown).

Green fluorescent protein (GFP) transformants of *Ptr* were described previously (Lorang *et al.* 2001). Fluorescent imaging of the GFP transformant was performed with a Leica DMRB epifluorescence microscope (Leica Microsystems, Wetzler, Germany) with filter sets for visualization of sGFP (Chroma Technology Corp., Rockingham, VT; Endow GFP Bandpass Emission Set: HQ470/40x exciter, Q495LP dichroic, HQ525/50m emitter). For DAPI staining, conidia were fixed on slides in 70% ethanol for l−2 hr at room temperature, stained with 0.5 µg/mL DAPI in McIlvaine’s buffer, pH 4.4, for 30 min, and examined with a Zeiss Universal microscope (filter set 48 77 02) with excitation between 340-370nm.

### PCR detection of a pathogen-specific secreted protein

To screen additional isolates for the presence of the pathogen-specific secreted protein, PTRG_11888, DNA was extracted from the isolates listed in Table S1 with the FastDNA SPIN Kit (MP Biomedicals, Solon, OH) per the manufacturer’s instructions. PCR was performed using GoTaq Flexi DNA polymerase (Promega). Primers used were PTRG_11888F2 (5′-TTCGGCCTTGCTCTACATTT-3′) and PTRG_11888R2 (5′-AAGCCGTTGCATCTACGAGT-3′) and the reaction components included 1× PCR buffer, 3.5 mM MgCl_2_, 0.8 mM dNTP, 0.5 µM of each primer, 0.25 U of GoTaq DNA polymerase and 20 ng of DNA. PCR conditions were 95° for 5 min, 30 cycles each of 95° for 1 min, 55° for 1 min, and 72° for 1 min, followed by a final extension at 72° for 7 min. For expression screening, total RNA was isolated as described for library preparation. First-strand cDNA synthesis was performed with the iScript cDNA Synthesis Kit (Bio-Rad Laboratories, Hercules, CA) per the manufacturer’s instructions. Reverse Transcriptase-PCR was performed using the same primers and reaction components described above.

### Determination of colinear blocks surrounding the 145-kb *ToxA*-containing region

A BLAST analysis of the coding regions of a 170-kb region of supercontig 4 (1.38−1.55 M) with *tblastn* against the contigs of the assemblies of *Cochliobolus heterostrophus* isolates C4 and C5, *Cochliobolus sativus*, and *S. nodorum* indicated that the 5′ and 3′ regions were present on a single scaffold in each organism and coding regions within the 145-kb *ToxA*-containing region were present on either one (*C. heterostrophus* and *C. sativus*) or several scaffolds (*S. nodorum*; data not shown). We then performed a blastn search against the *C. heterostrophus* C4 assembly and the *de novo* assembly of the Illumina-sequenced isolates, and a specific region of scaffold 37 in *C. heterostrophus*, a single contig in the SD20-NP genome, and two contigs in the DW7-ToxB genome were identified as having sequence that was present in these flanking regions. These sequences were aligned in progressiveMAUVE (Darling *et al.* 2010) and colinear blocks were defined with default parameters.

## Results and Discussion

**Figure 1:**
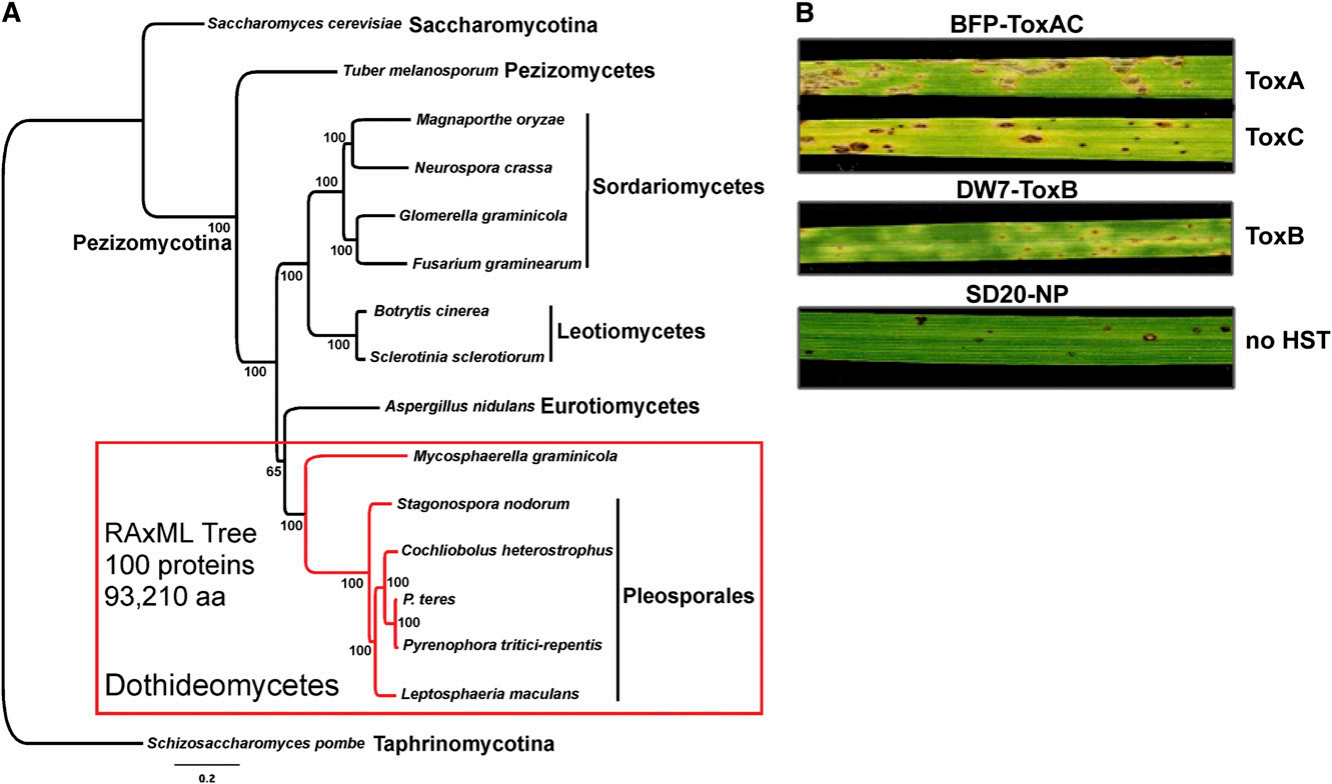
Phylogeny of *Pyrenophora tritici-repentis* and symptoms induced by the three sequenced isolates. RAxML maximum likelihood phylogenetic tree (best tree -nL1505566.555282) inferred from a 100-protein superalignment comprising 93,210 amino acid positions (A). Subphyla (-mycotina) and classes (-mycetes) of the phylum Ascomycota are shown and numbers near nodes are bootstrap partitions. (B) ToxA and ToxC symptoms induced by BFP-ToxAC (on Glenlea and 6B365, respectively; top 2 leaves), ToxB symptoms induced by DW7-ToxB (on 6B662; middle leaf), and the resistant reaction produced by the nonpathogenic SD20-NP (on Auburn; bottom leaf).

Dothideomycetes is the most diverse class of the phylum Ascomycota and includes a total of 14 orders that are distributed across two major subphyla, Pleosporomycetidae and Dothideomycetidae (Schoch *et al.* 2009a, 2006). The ancestral ecology of the class is estimated to be a plant decomposer with multiple origins of diverse ecologies including plant pathogens, lichens, marine and rock-inhabiting fungi (Schoch *et al.* 2009a). Phylogenetic analyses of genome-scale data (Figure 1A) support the monophyly of Dothideomycetes and infer a relatively close relationship with Eurotiomycetes. This finding is consistent with the distribution of certain morphological and life history characters including the formation of ascostromatic sporocarps and the production of bitunicate asci (Schoch *et al.* 2009b, Spatafora *et al.* 2006).

*Pyrenophora tritici-repentis* (*Ptr*) is a plant pathogen in the order Pleosporales and family Pleosporaceae. Pleosporales contains numerous agronomically important plant pathogens including species of *Cochliobolus*, *Leptosphaeria*, and *Stagonospora* (Schoch *et al.* 2009a). *Pyrenophora* contains important pathogens of cereal crops and individual species are typically associated with disease on only one host. However, these same pathogens have been isolated from a range of graminaceous species that may serve as an inoculum reservoir (De Wolf *et al.* 1998; Friesen *et al.* 2005; Liu *et al.* 2011; Medd *et al.* 2003; Postnikova and Khasanov 1998). *Ptr* is most associated with disease on wheat, but can cause lesions on barley, rye, and wild grasses (Ali and Francl, 2003; Krupinsky 1982). Interestingly, isolates identified as *Ptr* that appear to be nonpathogenic on wheat (from this point referred to as non-pathogenic) are often isolated from native grasses as well as wheat; whether this is due to sampling bias or actual host preference is currently unknown. All isolates for which sequencing data were acquired for this study have been shown by weighted parsimony analysis of combined ITS, *gpd*, and *MAT-2* HMG box sequence data to belong to the species *P. tritici-repentis* (Andrie *et al.* 2008).

### Comparison of *P. tritici-repentis* genomes revealed greater genetic divergence between the pathogenic and nonpathogenic isolates, suggesting genetic segregation

#### Generating the reference genome for P. tritici-repentis:

We generated a high-quality genome assembly of the race 1 isolate, Pt-1C-BFP. Race 1 is the most prevalent race found in wheat-growing regions in which tan spot is a major disease, and much of the molecular genetics and applied research have been conducted on race 1 isolates. Pt-1C-BFP is a subculture of the field isolate Pt-1C, from which the first host-selective protein toxin, ToxA, and its coding gene were characterized (Ciuffetti *et al.* 1997; Tomas and Bockus, 1987; Tomas *et al.* 1990; Tuori *et al.* 1995). Race 1 isolates also produce ToxC (Gamba *et al.* 1998; Lamari and Bernier 1991; Ballance *et al.* 1989), which appears to be a low-molecular-weight molecule that is likely the product of either a nonribosomal peptide synthetase or polyketide synthase modified in a biosynthetic pathway (Effertz *et al.* 2002). ToxA and ToxC induce necrosis and chlorosis, respectively, on toxin-sensitive cultivars (Figure 1B). In addition, data indicate that this isolate produces other uncharacterized proteinaceous toxins (Tuori *et al.* 1995). We will refer to this reference isolate as BFP-ToxAC.

The high-quality genome assembly of BFP-ToxAC presented here was generated by Whole Genome Shotgun Sanger DNA sequencing. Sequence from the entire genome was generated from paired-end reads of plasmids (insert sizes of four and 10 kb), and fosmids (insert size ~40 kb), and assembled with an improved version of Arachne [Table S2 (Jaffe *et al.* 2003)]. This genome assembly consisted of 47 sequence scaffolds with a total length of 37.8 Mb and an N_50_ scaffold length of 1.99 Mb (Table 1). More than 96% of the sequence had quality scores of at least 40 (*i.e.*, one error for every 10^4^ bases).

**Table 1:** *Pyrenophora tritici-repentis* genome assembly statistics

Assembly statistics	BFP-ToxAC
Coverage	6.93 (6.19 q > 20)
Assembly size	37.84 Mb
Total contig length	37.21 Mb
Scaffolds	47
Scaffold N50	1.99 Mb
Contigs	703
Contig N50	115.53 Kb
Q40	96.51%
Linkage groups	11
GC content	50.98%
Protein-coding genes	12,141
tRNA genes	129

q, quality score.

A whole-genome optical map of the sequenced isolate was constructed, and the genome size is estimated to be ~40 Mb using the Optical Mapping System (Dimalanta *et al.* 2004; Lin *et al.* 1999; Zhou *et al.* 2007, 2009). This optical map consists of 11 optical map contigs, with an average of ~98X physical coverage, each of which represents a single chromosome with clearly defined telomeric ends. Alignments were made between optical maps and the *in silico* maps of the sequence scaffolds based on the restriction enzyme recognition sequence and the lengths of the restriction fragments using map aligner software developed at the Broad Institute. The optical maps and sequence assemblies were highly congruent with more than 96% of the assembled scaffolds aligned to the optical map. The optical maps also allowed the assembled sequence scaffolds to be ordered and oriented (Table S3). Overall, more than 90% of the optical maps were covered by assembled sequence scaffolds.

#### Sequencing of additional P. tritici-repentis isolates from different races:

**Figure 2:**
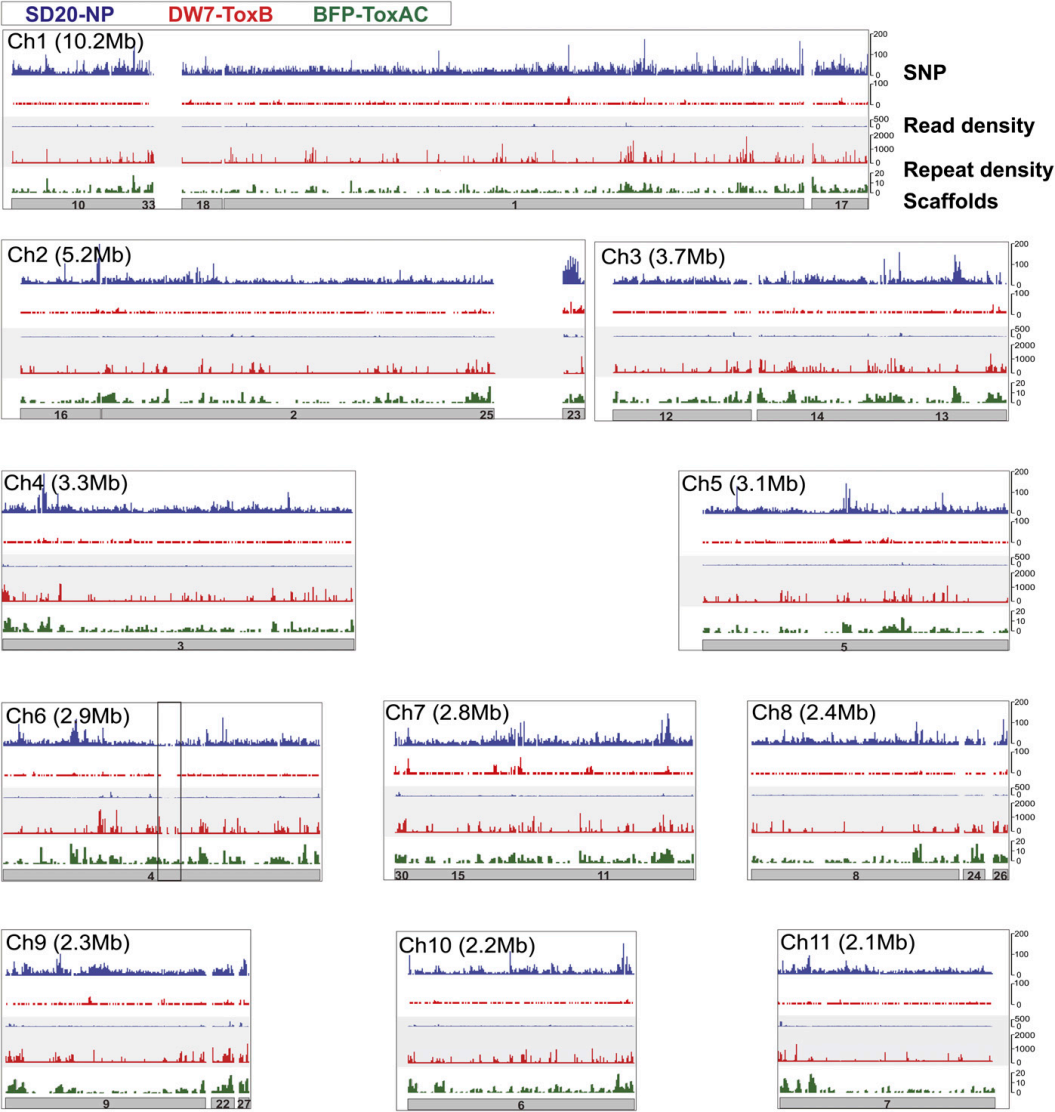
Mapping of sequence reads of resequenced isolates relative to the reference genome of *Pyrenophora tritici-repentis*. Schematic represents the reference genome scaffolds (gray bars with supercontig numbers) mapped to each chromosome as defined by the reference optical map. The border box reflects chromosome size as indicated next to the chromosome number and white spaces between the scaffolds represent gaps in the sequence assemblies. Repeat density (green) in the reference isolate, and read (gray central panel) and SNP (top panel) densities of the Illumina-sequenced isolates (DW7-ToxB-red, SD20-NP-blue), were mapped per 10 kb of the high-quality genome assembly based on the reference isolate (BFP-ToxAC) of *P. tritici-repentis*.

The authors of several studies have shown that *Ptr* populations have a high degree of genetic variability (Aboukhaddour *et al.* 2011; Friesen *et al.* 2005; Leisova *et al.* 2008; Leisova-Svobodova *et al.* 2010b; Moreno *et al.* 2008; Singh and Hughes, 2006; Pujol Vieira Dos Santos *et al.* 2002), and some suggest that there might be greater differences between pathogenic and nonpathogenic isolates (Aboukhaddour *et al.* 2011; Leisova-Svobodova *et al.* 2010b; Lepoint *et al.* 2010). Furthermore, karyotyping of multiple isolates revealed high chromosomal plasticity, with a smaller predicted genome size for all nonpathogenic isolates compared to their pathogenic counterparts (Aboukhaddour *et al.* 2009; Lichter *et al.* 2002; Martinez *et al.* 2004). In an effort to understand the population structure and the evolution of pathogenicity of *Ptr*, we generated over 100X sequence coverage for a nonpathogenic race 4 isolate (SD20-NP) and pathogenic race 5 isolate (DW7-ToxB) employing Illumina sequencing technology (Tables 2 and Table S4). No active HSTs have been reported in isolate SD20-NP, whereas DW7-ToxB produces at least one HST, ToxB, whose gene must be present in multiple copies to produce significant levels of disease (Figure 1B) (Aboukhaddour *et al.* 2012; Amaike *et al.* 2008; Ciuffetti *et al.* 2010; Strelkov *et al.* 2002). Mapping of Illumina reads from these two isolates to the reference genome assembly showed that fewer of those from SD20-NP can be mapped to the reference genome (Figure 2, gray panel) compared with DW7-ToxB (85 *vs.* 93%, respectively). A prediction of single-nucleotide polymorphisms (SNPs) present in each isolate compared with the reference revealed approximately 10 times as many SNPs in SD20-NP compared with DW7-ToxB (Figure 2, top panel). In addition, mapping of SNP density (10-kb window) onto the reference genome shows uniformity in the distribution of elevated SNP density between SD20-NP (blue histogram, top panel, Figure 2) and the reference. Taken together, these data suggest that SD20-NP is more divergent from the pathogenic reference isolate than is DW7-ToxB. Furthermore, the patterns of sequence variation among these three sequenced isolates suggest a lack of genetic exchange between the pathogenic and nonpathogenic populations, although it is possible that the genome integration might be too small to detect at the 10-kb window scale.

**Table 2:** *Pyrenophora tritici-repentis* Illumina sequencing statistics

Sequencing Statistics	DW7-ToxB	SD20-NP
Average depth	62X	111X
Maximum depth	2894X	5623X
Number PE reads mapped	34,248,040	67,243,074
PE reads mapped	93%	85%
PE pairs mapped	98%	97%
SNP found	86%	84%
Homozygous SNP	7429	73,190
SNPs/bp	5008	508
Multiallellic SNP	1250	6648
Small indels found	71%	71%
Small indels	1843	4282
Multiallellic indels	377	1660

SNP, single-nucleotide polymorphism; PE, paired-end.

### Increased content of transposable elements in pathogenic isolates of *P. tritici-repentis* may contribute to population divergence

**Figure 3:**
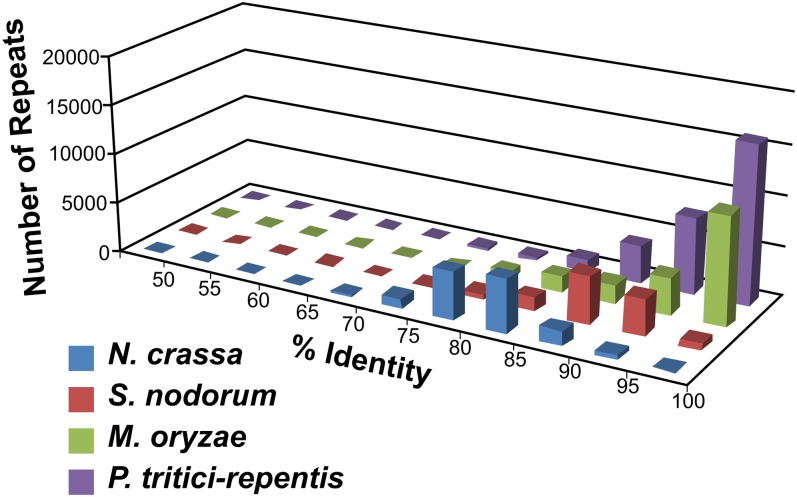
Repeat identity in the genome sequence of *Pyrenophora tritici-repentis*. The graph represents a comparison of repeat similarities between *Neurospora crassa, Stagonospora nodorum, Magnaporthe oryzae*, and *Pyrenophora tritici-repentis*. An identity was assigned to each repetitive sequence based on its similarity to other repeats in the genome (naturally grouped into one repeat family). The number shown on the y-axis is a collection of all repeats identified in the genome sequences (minimal 400 bp, 50% identity) and binned into groups according to the sequence identity present in that repeat family.

A CrossMatch repeat analysis was performed on the reference genome and indicated that approximately 16% of its content is repetitive DNA, and 81% of the repeat content is 95–100% identical (Figure 3), very high numbers when compared with most other sequenced fungal genomes. This finding suggests that repeat proliferations within the reference genome have amplified recently and that surveillance mechanisms, such as RIP (Cambareri *et al.* 1989, Selker *et al.* 1987), are inactive, despite the presence of a *Neurospora crassa rid* homolog (PTRG_05633), known to be required for RIP (Freitag *et al.* 2002). High identity in a high percentage of repeats is similar to what was detected in the genome of *Magnaporthe oryzae*, which is able to undergo RIP, but apparently far less efficiently than *N. crassa* (Dean *et al.* 2005). Examination of individual repeat families in *Ptr* for RIP-like mutations by RIPCAL (Hane and Oliver 2008) indicated that of the 98 families that contain members of sufficient length to be targets of RIP, only 10 contain family members that show CpA to TpA bias in all mutations present (Table S5), but not all family members contain these types of mutations. Additionally, 12 families have one or two members that show slight CpA to TpA bias. These data are consistent with findings that *Ptr* is most similar to fungi that do not use RIP as a genome defense mechanism (Clutterbuck 2011). Taken together, these data suggest that if RIP is functional in *Ptr*, the efficiency is low.

We can access the common repeats among the three sequenced genomes if we allow the multiple placement of repetitive sequences when mapping the reads from the additional sequenced isolates. Increased density of mapped reads from the Illumina-sequenced pathogenic isolate correlates well with the location of repeats in the reference, *i.e.*, where there is a high density of repeats in the reference there is a high density of reads from DW7-ToxB mapped to the genome (Figure 2, gray panel *vs.* green histogram). There are far fewer regions of high coverage from SD20-NP and only a few cases in which coverage from SD20-NP is greater than coverage from DW7-ToxB. Mapping of ESTs from the reference and an additional pathogenic isolate, SO3-P (Andrie *et al.* 2007), resulted in a greater number of mapping events than the number of input transcript reads, which indicated that there are transcripts of repeats being produced in the pathogenic isolates that map multiple times to the reference genome (Table S6). In contrast, only 79% of the EST sequence reads from SD20-NP mapped to the reference, supporting the hypothesis of greater divergence between pathogenic and non-pathogenic isolates of *Ptr*. Together, these data indicate that the pathogenic isolates (the high-quality reference assembly of BFP-ToxAC, the Illumina-sequenced DW7-ToxB, and the ESTs from SO3-P) share a similar repeat profile that is different from that in the non-pathogenic isolate (the Illumina-sequenced SD20-NP).

A *de novo* repeat analysis of the reference genome assembly indicated that long terminal repeat (LTR) retrotransposons and DNA transposons account for the largest percentages of repeat elements at 40.5% and 36%, respectively (Table 3 and Figure S1A−C). Approximately 1% of the repetitive sequences were MITE elements; one highly conserved family, with > 200 members that are well distributed across the genome, represents the majority of these elements. Several of the repeat families have members that are transcribed both during in culture and *in planta* growth of BFP-ToxAC, and many of these elements are also transcribed in SO3-P, but far fewer are transcribed in SD20-NP (Table S5). In fact, the most abundant transcript present in the *in planta* library belongs to a pathogen-specific short interspersed nuclear element that contains a putative tRNA^ser^ at its 5′ end. Although replication of this element has resulted in 25 copies of a tRNA^ser^ with a GCT anti-codon, there is no evidence of serine codon bias in the *Ptr* genome (Table S7). The genome of the protist parasite *Entamoeba histolytica* also contains expansions of tRNAs for which no correlation between copy number and codon usage could be made (Loftus *et al.* 2005).

**Table 3:** Repetitive content of *Pyrenophora tritici-repentis* isolates

	BFP-ToxAC			% Shared	
Repeat	Total Length, nt	% of Repetitive	% of Genome	DW7-ToxB	SD20-NP
DNA transposon	2,295,943	36.4	6.1	46.5	16.5
MITEs	74,049	1.2	0.2	0.8	1.1
LTR retrotransposon	2,553,686	40.5	6.7	28.1	20.6
Non-LTR retrotransposon	405,556	6.4	1.1	7.2	26.3
Unknown TEs	685,925	10.9	1.8	17.4	35.5
Others (genes)	159,636	2.5	0.4	ND	ND
Simple repeats	84,534	1.3	0.2	ND	ND
Low complexity	50,671	0.8	0.1	ND	ND
Total	6,310,000		16.7		

Represent nt covered by custom repeat library. MITE, miniature inverted-repeat transposable element; LTR, long terminal repeat; TE, transposable element; ND, not determined.

Determination of the shared repeat elements between SD20-NP/DW7-ToxB and the reference showed that the two most common types of repeat elements in BFP-ToxAC are also the most common elements shared with the pathogenic isolate, DW7-ToxB (Table 3); 46.5% and 28.1% of the reads that mapped to repeats in the reference represented shared DNA transposons and LTR retrotransposons, respectively. The five families in the reference genome to which the most reads map are all DNA transposons, three with characteristics of the *hAT* (*hobo-Activator-Tam3*) superfamily and two with characteristics of the *Tc1/Mariner* superfamily (Table S8); one showing high similarity to *Molly* from *S. nodorum*. In contrast, the largest class of shared repeats between the non-pathogenic and the reference isolate are those that are not well represented in the reference, the non-LTR retrotransposons (6.4% in the reference and 26.3% shared between isolates) and unknown elements (10.9% in the reference and 35.5% shared between isolates). Most repeat family reads from the non-pathogenic isolate are from non-LTR retrotransposons, similar to the *Tad1* element of *Blumeria graminis*, others are in novel, previously undescribed elements.

Clamped homogeneous electric field gel analysis had indicated previously that the genome size of DW7-ToxB was greater than that of SD20-NP (Martinez *et al.* 2004). In addition, Southern blot analysis of BFP-ToxAC and SD20-NP with probes from LTR retrotransposons and DNA transposons indicated that these elements were present in far fewer copies or not at all in the non-pathogen when compared with the pathogen. The difference in repeat elements between the pathogenic and nonpathogenic isolates suggests that amplified transposable elements (TEs) are likely to be the major underlying cause for the differences in genome size. K-mer analysis (Kurtz *et al.* 2008) of Illumina reads from DW7-ToxB and SD20-NP confirmed the repetitive nature of DW7-ToxB (Table S9). In a subsample of reads at 1X depth, 95% of 16-mers in SD20-NP were present in fewer than 10 copies as opposed to 88% in DW7-ToxB. In addition, a greater percentage of 16-mers are present 100 times or more in the DW7-ToxB reads *vs.* those from SD20-NP reads (1.68 *vs.* 0.01%, respectively). TEs can lead to chromosomal rearrangements, which can lead to reduced fertility in heterozygous progeny (Daboussi 1997; Delneri *et al.* 2003). Thus, the presence of TEs may accelerate the rate at which this incompatibility occurs (Bennetzen 2000; Coghlan *et al.* 2005).

**Figure 4:**
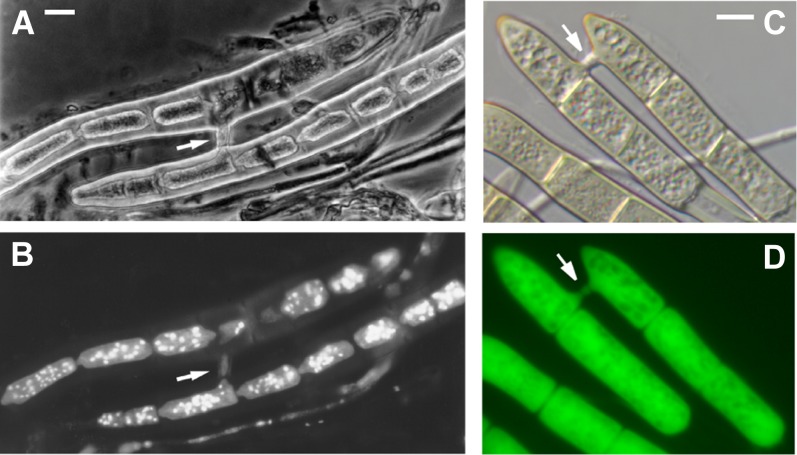
Conidia of *Pyrenophora tritici-repentis* connected by anastomosis bridges. (A) Phase-contrast microscopy of conidial anastomosis in *Ptr*. (B) The same conidia shown in A stained with DAPI and visualized under an ultraviolet filter to image nuclei; Note the loss of nuclei from the upper cell. (C) Nomarski optics (D) and fluorescence microscopy of anastomosed conidia of *Ptr* expressing green fluorescent protein. Arrows indicate the anastomosis bridges. Scale bars = approximately 5 µm.

Although *Ptr* is a homothallic fungus, outcrossing (heterothallic capability) of differentially marked antibiotic-resistant isolates in the laboratory has been shown to occur at low frequency (L. Ciuffetti and J. Gaventa, unpublished data; McCallum *et al.* 1994); however, the extent of outcrossing that occurs in nature remains unknown. In the absence of outcrossing, nuclear transfer could take place through hyphal fusion (data not shown) or conidial anastomosis (Figure 4). Conidial anastomosis has often been observed during conidiation of single isolates of *Ptr* but has not yet been described between isolates. With or without evidence of outcrossing, the data obtained from sequencing suggests a correlation between the genetic isolation of the pathogenic and nonpathogenic isolates and the expansion of TEs, most significantly, DNA transposons, which could potentially lead to speciation.

### The *P. tritici-repentis* genome exemplifies the contribution of transposable elements to pathogenicity and the creation of new genes

Transposable elements are noted for their mobility within the genome and their ability to amplify their copy number. TEs can lead to changes in gene structure and regulation through various activities; for example, transposition, insertion, excision, and recombination (Daboussi, 1997, Daboussi and Capy 2003). In the *Ptr* reference genome, we found evidence for the amplification of non-TE-encoding genes, recombination of genes into new coding regions, which results in protein diversification, and association with putative HGT events, all attributable to the action of TEs. Additionally, there are examples for how TE-driven phenomena are directly impacting pathogenicity. Among the characterized repetitive sequences in the reference genome, about 2.5% were annotated as functional genes. While a comparative analysis of Pfam domains present in the *Ptr* genome with those of other fungal wheat pathogens reveals a typical profile of an ascomycete fungal genome (Figure S2A−C), at the same time, we observed strong footprints of TEs and their clear contribution to pathogenesis.

#### Transduplications create novel gene and dosage effects:

TEs can increase gene copy number through the acquisition of segments of the genome and their subsequent movement to new locations. Although these genes may be fragmented, they may provide material for new composite genes. One possible explanation for the expansion of individual domains and genes is “transduplication” (Juretic *et al.* 2005), which refers to the process by which DNA transposable elements acquire cellular genes or gene fragments between their terminal inverted repeats (TIR) and replicate them throughout the genome. Transduplication is well documented in plants, especially rice and Arabidopsis, and it has been shown that expression of the replicated gene fragments can alter cellular gene expression (Hanada *et al.* 2009; Hoen *et al.* 2006; Jiang *et al.* 2004). In addition, chimeras from gene fragments within transduplicating transposons and a preference for incorporation of DNA-binding proteins and transcription factors have been documented (Hanada *et al.* 2009; Hoen *et al.* 2006). Compared with other TE-mediated gene duplication events, transduplication specifies the event in which a gene/gene fragment is integrated as part of the transposable element, which results in a greater possibility that the incorporated gene/gene fragment would be amplified through replication of the TE.

**Figure 5:**
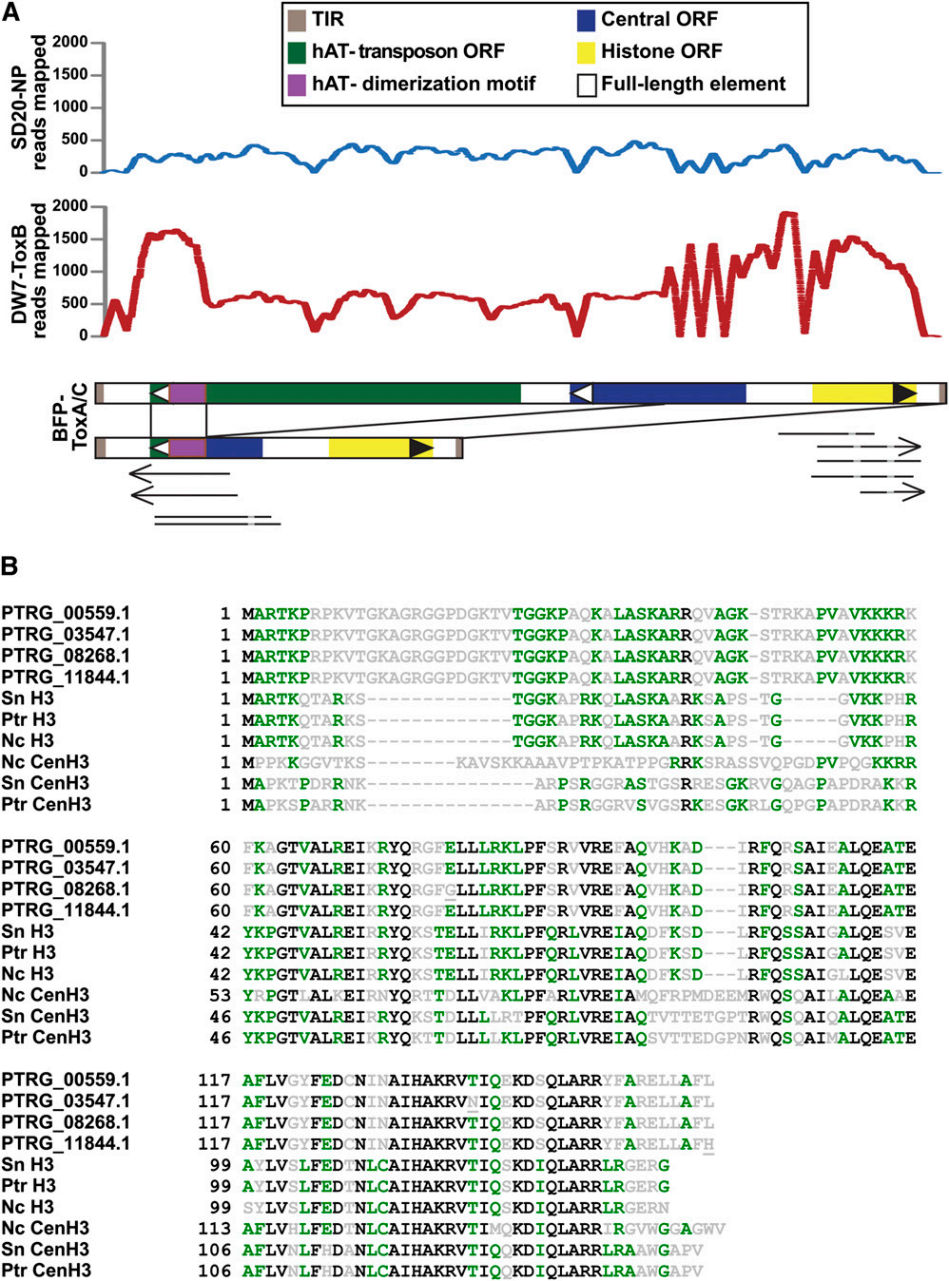
Transduplication of a histone H3-like protein family in the genome of *Pyrenophora tritici-repentis*. (A) Two representatives of a transduplicating family of DNA transposable elements in the BFP-ToxAC reference genome are shown with the characteristic motifs of the elements indicated (brown box = terminal inverted repeats (TIR); green box = *hAT* transposon coding regions; purple box = *hAT* dimerization motif; blue box = central open reading frame (ORF) coding region; yellow box = histone H3 coding region; black boundary box = full-length element). The large, likely autonomous element is approximately 5.6 kb in length. Black lines between elements illustrate a pathogen-specific recombination event that results in a smaller element of approximately 2.3 kb. The graphs show coverage of reads obtained from the pathogenic DW7-ToxB and nonpathogenic SD20-NP isolates. Arrows and lines below the element representation indicate alignments of ESTs detected in various libraries (arrows = poly(A) tails, light gray = introns). (B) Alignment of novel histone H3-like (H3L) proteins identified in *Ptr* with *bona fide* histone H3 variants from *Ptr*, *S. nodorum*, and *N. crassa*. Four variants of full-length H3L exist in *Ptr*, all are different by a single aa change (underlined; also see Table S9). Two changes in H3L alleles result in frameshift mutations, yielding 5′ and 3′ truncated versions of H3L. Most (21) copies are identical to PTRG_00559.1 in amino acid and DNA sequence. Completely conserved residues are shown in black, similar residues in green and variable residues in gray.

The first indication that transduplication might be occurring in *Ptr*, and indeed the first indication for transduplication in fungi, was the presence of an unusually large number of histone H3-like globular domains (PF00125, core histone H2A/H2B/H3/H4) in the reference genome (Figure S2B). A typical filamentous fungal genome has two or three such family members but no more (Hays *et al.* 2002). Examination of the genomic regions that contain these domains resulted in the discovery of two forms of a related repeat element that contains a *hAT* dimerization domain and carries additional open reading frames (ORFs) between their TIRs, one of which encodes a histone H3-like protein, “H3L” (Figure 5). The longer, ~5.6-kb form of the transduplication was present 10 times in the reference genome in almost identical copies. It encodes a full-length *hAT* transposase gene and a central ORF of unknown function, as well as the H3L gene. The short, 2.3-kb form of the repeat, also highly similar in DNA sequence, likely arose from recombination of the transposon ORF and the central ORF at a ‘gactat’ sequence present in both, and was found in 16 copies in the reference genome. The result of this recombination event is an in-frame chimeric protein that contains the N-terminus of the central ORF at its 5′ end and the C-terminus of the transposon ORF at its 3′ end and still retains the transposon *hAT* dimerization domain. Based on read-mapping data, both DW7-ToxB and SD20-NP contain the large, likely autonomous element, with a predicted 25 and five copies, respectively. However, only the two sequenced pathogens contain the recombinant, non-autonomous element, with an estimated 25 copies in DW7-ToxB.

We assembled parts of the element from Illumina reads, and these partial sequences contain numerous point mutations when compared with the 26 almost identical H3L repeats (43 mutations in four partial copies in SD20-NP and DW7-ToxB *vs.* 18 total mutations in 26 complete copies in the reference genome; Figure S3; data not shown). Mapping of the Illumina reads to the short and long elements found in the reference genome also suggests that point mutations or indels are present in the elements from DW7-ToxB (Figure 5A, Figure S3, and data not shown). Taken together, our analyses suggest that this family of repeats was introduced in a precursor of the sequenced races and has only relatively recently expanded in BFP-ToxAC as all 26 H3L genes are very similar. Although there is no evidence that the full-length transposon is mobile, ESTs for the H3-like protein are present in the libraries from both pathogenic and non-pathogenic isolates and the pathogenic isolate SO3-P produces transcripts that suggest that the chimeric protein is expressed (Table S5).

As a result of transduplication, there is a large expansion of H3-type proteins in the *Ptr* genome. One may wonder where these additional H3Ls originated. Alignments to other H3 genes showed that overall similarity is greatest to *bona fide* H3 genes from *Candida* species but the amino-terminal tail closely resembles that of H3 from the mosquito *Culex quinquefasciatus* (EDS45503.1), the protozoan *Cryptosporidium parvum* (EAK87921.1), and the rabbit, *Oryctolagus cuniculus* (XP_002721691.1) (data not shown). As suggested previously for the whole element, the 26 ORFs have extremely similar DNA sequences, as 12 are identical, six contain one silent mutation, two have two silent mutations, one has three silent mutations, three have a single missense mutation each, and only two contain frameshift mutations that result in one 5′- and one 3′-truncated H3 variant (Figure 5B and Table S10). Alignments to *bona fide* fungal H3 and centromere-specific H3 variant (CenH3 or CenpA) showed that the novel H3Ls belong to neither group (Figure 5B and Figure S4). True H3 proteins are extremely well conserved especially at their amino-terminal tails, which are subject to post-translational modifications, and their globular domains (Figure S4A). All 26 *Ptr* H3Ls have insertions in the amino-terminal tail and regions within conserved α-helices in the histone fold domain are also variable, matching neither H3s (Figure S4A) nor CenH3 (Figure S4B) completely, suggesting that these genes, while conserved in *Ptr* race 1, may have undergone drift.

An expansion of core histone proteins also has occurred in the obligate phytopathogenic rust fungus, *Puccinia graminis* (Table S11), but it is likely that this has occurred through amplification of the H3-H4 gene cluster, because four of six copies are still linked, as is typically the case in fungi (Hays *et al.* 2002). The H3L copies in *P. graminis* are not as similar as those in *Ptr*, nor are they linked to a potentially active transposon. It is possible, though unlikely, that transcription of the *H3L* genes within the transduplications is impacting cellular gene expression, including perhaps expression of the *bona fide* H3. It also remains possible that translation of this H3 isoform is occurring, although it is quite different from typical fungal H3 proteins. Because H3 is an essential component of nucleosomes it is possible that altered expression levels or aberrant nucleosomes can alter chromatin in specific regions or generally; however, whether this occurs in *Ptr* remains to be determined. Although attempted, expression of the *Ptr* transposon in a heterologous host has not been observed yet, but additional studies are underway (V. Manning, M. Freitag, and L. Ciuffetti, unpublished data).

To test for additional transduplication events in *Ptr*, we looked for nontransposon-associated Pfams in the repetitive DNA component of the genome (Table S12). We found an abundance of osmosensory transporter coiled-coil domains (PF08946) and domains associated with nonribosomal peptide synthetases [AMP-binding enzyme (PF00501), condensation (PF00668), methyltransferase (PF08242), and phosphopantetheine attachment (PF00550) domains]. Indeed, the osmosensory transporter coiled-coil domains were present in what appeared to be an ORF that had been sequestered between the TIRs of a DNA transposon of the *Tc1/Mariner* superfamily and multiplied across the genome (Figure S5). There are 46 members of this repeat family in the reference genome, 31 of which are almost identical and contain full-length copies of both coding regions. Although this form of transduplication was not detected in the nonpathogenic isolate SD20-NP, the read alignment from the pathogenic isolate DW7-ToxB suggested not only the existence of the same transduplicated form, but also suggested a similar number of duplications. Several transcripts from the culture libraries of the pathogens BFP-ToxAC and SO3-P map to this repeat family and, as expected, no transcript was found in the culture library of the nonpathogen, isolate SD20-NP. Interestingly, coding sequences similar to both the transduced ORF and the transposon are present in tandem on a BAC from the apple pathotype of *Alternaria alternata* (AM-BAC-13) that contains the genes necessary for the production of AM-toxin. Although the functional significance of transduplication events in the *Ptr* genome has not yet been empirically validated, our observations demonstrate the genetic plasticity achieved through the expansion of TEs.

#### Horizontal transfer, duplication, and diversification—the birthplace of virulence factors:

##### Horizontal transfer of ToxA*:*

The *Ptr* genome project revealed a wealth of evidence to suggest that amplification of TEs in the genome created an evolutionary cradle that enables rapid change and created new virulence factors. In fact, the ways in which TEs can affect the structure and evolution of individual genes appear to be almost unlimited. Significantly, researchers have proposed that TEs can move horizontally in eukaryotes and therefore can be sensitive indicators of the potential for HGT (Keeling and Palmer 2008). *ToxA* in *Ptr* appears to serve as a good example to illustrate the impact that HGT can have on pathogenicity. ToxA is the proteinaceous HST that is produced by the majority of the virulent *Ptr* isolates collected from wheat fields worldwide (Antoni *et al.* 2009; Benslimane *et al.* 2011; Lamari and Strelkov, 2010; Lamari *et al.* 2005; Leisova-Svobodova *et al.* 2010b; Shaukat and Francl 2002). ToxA is a potent pathogenicity/virulence factor, inducing rapid cell death in toxin-sensitive cultivars (Pandelova *et al.* 2012), and the mode of action is fairly well understood (Faris *et al.* 2010; Kwon *et al.* 1998; Manning and Ciuffetti, 2005; Manning *et al.* 2008, 2009; Pandelova *et al.* 2009, 2012; Rasmussen *et al.* 2004; Sarma *et al.* 2005; Vincent *et al.* 2011).

HGT of an 11-kb region of DNA that contains the *ToxA* gene flanked by two TEs has been suggested between *Ptr* and *S. nodorum*, two wheat-adapted species in the fungal order Pleosporales (Friesen *et al.* 2006). The sequence identity between the 11-kb regions is remarkably high at 98–100%, whereas the nucleotide sequence identity for a typical housekeeping gene, glyceraldehyde 3-phosphate dehydrogenase, is 80% (Friesen *et al.* 2006). The *ToxA* genes from a diverse collection of *S. nodorum* isolates have been sequenced and there are at least 13 haplotypes found in a global population. In contrast, a sampling of predominantly North American isolates of *Ptr* revealed fewer haplotypes (Stukenbrock and McDonald, 2007; Tan *et al.* 2010). The greater diversity of *SnToxA* in the *S. nodorum* population has led to the hypothesis that this species was the donor in the HGT event (Friesen *et al.* 2006), although other scenarios are still plausible.

**Figure 6:**
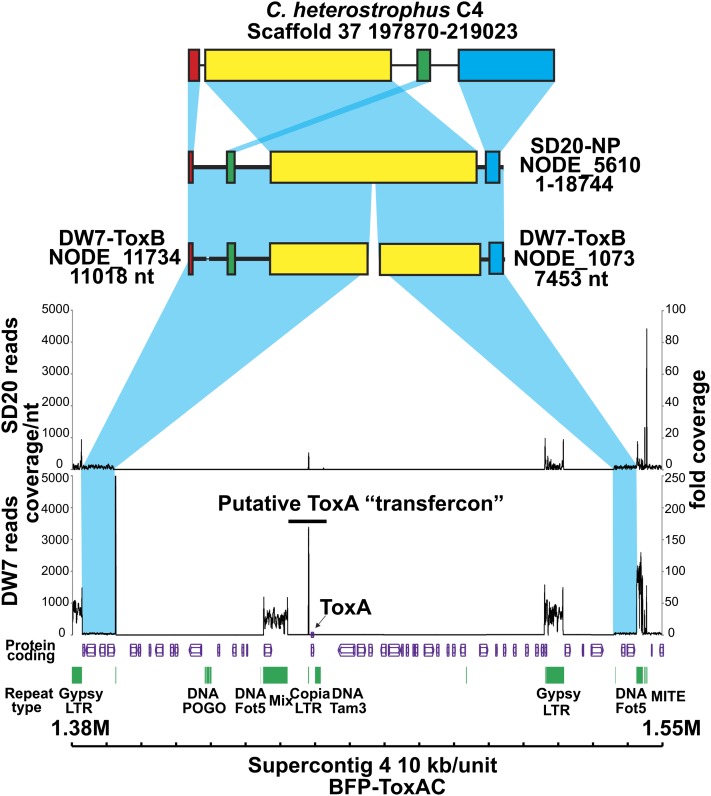
Absence of the 145-kb *ToxA*-containing region in the pathogenic DW7-ToxB and non-pathogenic SD20-NP isolates of *Pyrenophora tritici-repentis*. Schematic includes the protein-coding regions (purple) and repeats (green) present within the 170-kb *ToxA*-containing genomic region in the BFP-ToxAC reference genome. The graphs show coverage of reads obtained from the pathogenic DW7-ToxB and nonpathogenic SD20-NP isolates. Coverage depth per nucleotide (nt) is indicated on the left and fold coverage is indicated on the right. Blue shading represents synteny between genomic scaffolds of *Cochliobolus heterostrophus* isolate C4, SD20-NP, DW7-ToxB, and flanking regions of the 170-kb *ToxA*-containing region of the reference. Similarly colored bars within the scaffolds indicate colinear blocks.

Karyotype analyses of different races of *Ptr* indicate that *ToxA* can be present on different chromosomes (Aboukhaddour *et al.* 2009; Lichter *et al.* 2002), which suggests it is either mobile within the genome or has been introduced independently several times. In the reference genome assembly *ToxA* is located on Chr 6, which is comprised of a single 2.788-Mb scaffold (scaffold 4). Mapping of the reads from the non-*ToxA*-containing isolates indicated that a ~145-kb region containing *ToxA*, as well as several types of repeat elements and other coding genes, is missing in the other isolates. Given a proposed HGT event into *Ptr*, we tested whether this 145-kb segment was inserted into the reference genome in this region of Chr 6 by comparing the flanking regions on single contigs in closely related species and in the Illumina-sequenced isolates. We found that the flanking regions were present on single contigs in the closely related species *C. heterostrophus* (Figure 6) and other sequenced Pleosporales (data not shown) and in the *de novo* assembly (Table S13) of the nonpathogenic isolate SD20-NP. In DW7-ToxB, the flanking regions are on two contigs; however, there is a repeat region at the end of one of the contigs that would most likely prevent *de novo* assembly into one larger contig. Therefore, it seems likely that this 145-kb region was inserted into this position in the reference genome; however, our analysis did not support the hypothesis that the donor of this entire 145-kb piece was *S. nodorum*, as only the 11-kb *ToxA*-containing region in the center of the larger 145-kb piece showed the high nucleotide similarity level. Given that the 145-kb region is flanked by remnants of repeat elements that are present in other regions of the *Ptr* genome, it is possible that intra or inter-chromosomal recombination was responsible for the insertion of the 145-kb piece into Chr 6 of the reference genome.

##### Duplication of ToxB:

**Figure 7:**
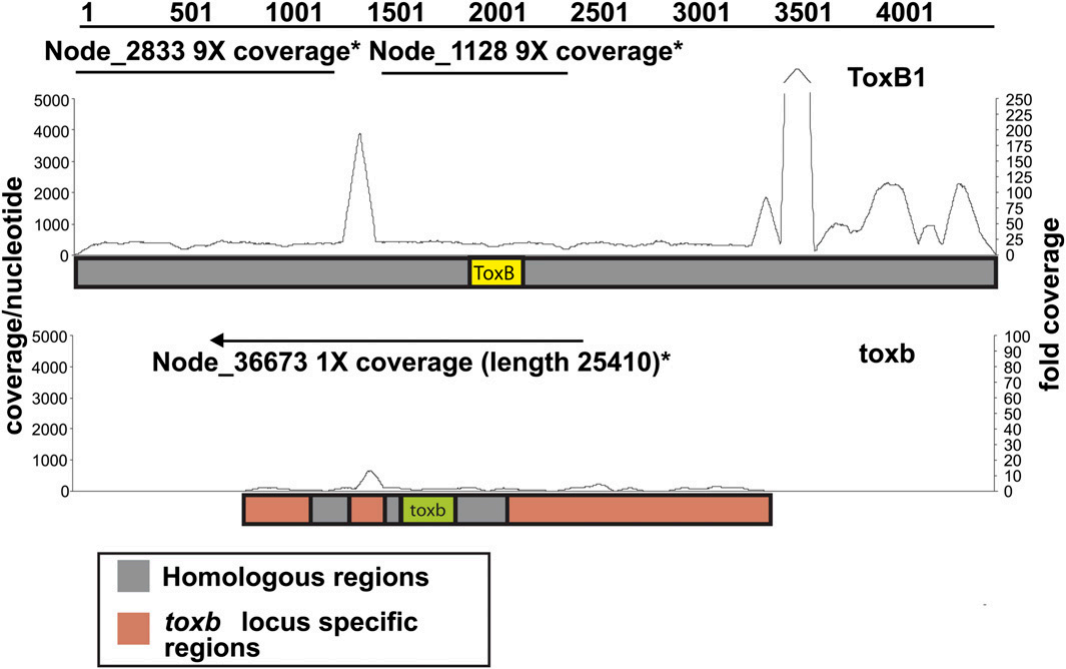
Read mapping to and *de novo* assembly of *ToxB*- and *toxb*-containing loci in the genome of *Pyrenophora tritici-repentis*. Schematic of Illumina sequence reads (line graph) of isolate DW7-ToxB mapped to the *ToxB1* locus (top: *ToxB1* locus; accession number: AY425480.1) and of SD20-NP mapped to the *toxb* locus (bottom: *toxb* locus; accession number: AY083456.2). Coverage depth per nt is indicated on the left and fold coverage is indicated on the right. Straight lines above the graphs depict the contigs present in the *de novo* assemblies of the Illumina-sequenced isolates. The arrow on the contig above the *toxb* locus shows how that contig extends beyond the locus.

ToxB is a prime example that illustrates how duplication mediated by TEs impacts pathogenesis of this species. Mature ToxB is a 64 aa cysteine-rich (6%) proteinaceous HST produced by races 6, 7 and 8 of *Ptr*, as well as the race 5 isolate, DW7-ToxB, that was sequenced as part of this study (Lamari *et al.* 2003; Martinez *et al.* 2001; Strelkov *et al.* 2002). The gene encoding ToxB displays copy-number variation depending upon the isolate (Lamari *et al.* 2003), and the severity of the disease produced by these isolates depends upon the copy number (Aboukhaddour *et al.* 2012; Amaike *et al.* 2008; Ciuffetti *et al.* 2010; Strelkov *et al.* 2002). It was previously estimated that DW7-ToxB, which produces a highly virulent ToxB phenotype, possesses nine *ToxB*-containing loci (Martinez *et al.* 2004). Cloning of six of these loci from DW7-ToxB showed that the *ToxB* sequence was identical in each and that regions upstream and downstream were highly homologous. A 197-bp insertion, likely a remnant of a *Ty1/Copia*-like retroelement, was present in different locations in three of the six loci and *Gypsy*-like retrotransposon remnants also were detected at each locus. *De novo* assembly of the DW7-ToxB reads produced two contigs that map completely within the largest locus, *ToxB1*, with a gap at the 197-bp insertion site (Figure 7). Consistent with the previous estimate of the number of *ToxB* loci in DW7-ToxB, the read coverage of these contigs is approximately ninefold. Read mapping suggested that there are many more copies of the 197-bp insert in the DW7-ToxB genome than there are ToxB-coding regions, which may explain why the two mapped contigs could not be assembled into a larger single contig. We also found that the previously defined locus-specific regions of the *ToxB*-containing loci are repeat rich and that these repeat sequences, when compared with the sequences of repeats in the reference genome, are primarily fragments of LTR (*Ty1/Copia*-, *Ty3/Gypsy*-, *Skippy*-like) and non-LTR retrotransposons (*Tad1*) (data not shown). The high repeat content surrounding *ToxB* in these loci suggests that transposition is playing a role in copy-number variation in *ToxB*-containing isolates.

Unlike most described HSTs, *ToxB* homologs are present across a broad range of plant-pathogenic ascomycetes, suggesting that this gene may have arisen in an early ancestor of the Ascomycota (Andrie *et al.* 2008). However, not all *ToxB* homologs may be functional in their host-pathogen interaction (Andrie and Ciuffetti 2011). For instance, the *ToxB* homolog in SD20-NP (Martinez *et al.* 2004), which we refer to as *toxb*, does not appear to encode a toxin active in wheat, most likely due to changes in amino acid sequence (Andrie and Ciuffetti, 2011, Figueroa Betts *et al.* 2011). A single *toxb*-containing contig, with approximately onefold coverage, was present in the *de novo* assembly of SD20-NP, consistent with the previous prediction of a single copy of *toxb* in this isolate (Martinez *et al.* 2004). The contig extends sequence information of the genomic region that contains *toxb* by approximately 10 fold (from ~2550 nt to >25,000 nt). A blastn search with this SD20-NP contig against the reference genome revealed a 10-kb stretch that is syntenic to a region of Chr 5, which optical mapping predicts to be 3.1 Mb. A previous study showed that *toxb* was localized to a 3.1-Mb chromosome in SD20-NP (Martinez *et al.* 2004); therefore, it is possible that the 3.1-Mb chromosomes of these two isolates are homologous. Sequence data provide ample evidence that highly mobile TEs around *ToxB* in pathogenic isolates provided a favorable environment for expansion of an active toxin gene in which dosage plays an important role in virulence. In addition, it leads to speculation that toxb might at one time have had a marginal function in pathogenesis, but that the lack of TEs in the SD20-NP genome, and therefore, the lack of opportunity to expand, resulted in pseudogenization.

##### Duplication and diversification of secreted proteins:

In addition to known HSTs, other candidate pathogenicity-related proteins associated with TEs also are present. One example is a family of small secreted cysteine-rich proteins (Table S14) that is present as a result of tandem duplication followed by diversification (*PTRG_11771*, *PTRG_11772*, *PTRG_11773*), and more recent duplication events (*PTRG_11773* and *PTRG_11346*) in the sequenced pathogenic isolates but is not expanded and diverged in the sequenced non-pathogenic isolate. PTRG_11772 is the most diverged member of this family, yet the cysteine residues in all of the family members are conserved. *PTRG_11773* is expressed during infection, as ESTs were found in the *in planta* expression library, suggesting that this protein may be involved in pathogenesis. A blast search of the NCBI nonredundant database indicated that, with the exception of one homolog (FOXB_14349) from the Arabidopsis-infecting strain of *F. oxysporum* (strain 5176), all other homologous proteins are present in cereal pathogens that are able to colonize wheat, including *P. teres f. teres* (PTT_08876), *Colletotrichum* (*Glomerella) graminicola* (GLRG_11876, GLRG_08163, GLRG_08162, GLRG_04750), and *Mycosphaerella graminicola* (MYCGRDRAFT_96981). Expansion of this protein family in *Ptr* and *C. graminicola* suggests that increased copy number of this gene may provide an advantage, which makes these genes compelling candidates for further studies of adaptation to a host-specific lifestyle.

##### Duplication and recombination creates pathogen-specific nonribosomal peptide synthetases:

Nonribosomal peptide synthetases (NRPS) are mono- or multimodular enzymes that produce diverse compounds with a multiplicity of functions (Schwarzer and Marahiel 2001). The most basic module contains an adenylation domain (A) followed by a thiolation domain (T), which are often accompanied by a condensation domain (C). Additional functions encoded in a module could include epimerization and N-methyltransferase activities. Certain plant-pathogenic fungi produce NRPSs that are known to be important for the biosynthesis of toxins involved in pathogenesis (Gardiner *et al.* 2004; Johnson *et al.* 2000; Walton 2006). When we surveyed the *Ptr* reference genome we found that several NRPS-coding genes (*NPS*s) were conserved between *Ptr* and other Ascomycota (Table 4). All *Ptr* isolates sequenced in this study contain *NPS 2*, *4*, *6*, and *10* (nomenclature adopted from the NRPSs of *Cochliobolus heterostrophus*), which are thought to be moderately conserved in the Ascomycota (Lee *et al.* 2005; Turgeon *et al.* 2008). They also encode a NRPS (PTRG_12015) that is similar to the NRPSs required for synthesis of the histone deacetylase (HDAC) inhibitor apicidin in *Fusarium incarnatum* (*APS1*) and HC-toxin in *Cochliobolus carbonum* (*HTS1*; Figure S6). In the reference genome, *PTRG_12015* is present within a cluster (Table S15), and almost all of the proteins in the cluster have EST support, suggesting that this is a functional biosynthetic cluster that is coordinately regulated. Homologs of 8 of the 11 proteins identified in the apicidin biosynthetic cluster (Jin *et al.* 2010) and four genes required for the production of HC-toxin (Walton 2006) are present in the *Ptr* cluster.

**Table 4:** Putative *Pyrenophora tritici-repentis* nonribosomal peptide synthetase genes

Locus	Best Hit*^a^*				*Cochliobolus* NPS (%id/sim)*^c^*	Repeated domains*^d^*	Assoc. with TE*^e^*	EST	Present (SNP)*^b^*	
	Org.	Acc.	(%id/sim)*^c^*	Rec.					DW7-ToxB	SD20-NP
Conserved										
PTRG_08276*^f^*	*Ch*	AAX09984.1	(*^g^*)	Yes	NPS2 (62/76)	No	No	Yes*^h^*	Yes (1)	Yes (2)
PTRG_01800*^f^*	*Ab*	AAP78735.1	(77/87)	Yes	NPS4 (72/84)	No	No	No*^h^*	Yes (1)	Yes (37)
PTRG_01683*^f^*	*Cm*	ABI51982.1	(77/86)	Yes	NPS6 (76/86)	No	No	No*^h^*	Yes (1)	Yes (10)
PTRG_00447*^f^*	*Ch*	AAX09992.1	(*^g^*)	Yes	NPS10 (91/96)	No	No	Yes*^h^*	Yes (0)	Yes (3)
PTRG_03139	*Ch*	AAX09994.1	(*^g^*)	Yes	NPS12 (70/80))	No	No	No	Yes (2)	Yes (10)
PTRG_09101*^f^*	*Sn*	XP_001791762.1	(72/80)	Yes		No	No	Yes	Yes (1)	Yes (39)
PTRG_09808	*Sn*	XP_001797376.1	(60/70)	Yes		No	No	No	Yes (0)	Yes (0)
PTRG_12015*^f^*	*Fi*	ACZ66258.1	(39/56)	Yes	HTS1 (35/51)	No	No	Yes	Yes (2)	Yes (18)
Hybrids										
PTRG_00649	*Mg*	XP_367899.2	(37/50)	No		No	Yes	No	Yes (1)	Yes (6)
PTRG_04244*^f^*	*Pa*	XP_001905191.1	(49/66)	No		No	No	Yes*^h^*	Yes (7)	Yes (76)
NPS pathogen-specific diversification										
PTRG_10128	*Ch*	AAX09983.1	(*^g^*)	No	NPS1- w/o 5′&3′ (61/70)	No	Yes	No	Divergent	No
PTRG_10433	*Cg*	XP_001226467.1	(28/43)	No		Yes	Yes	Yes*^i^*	Divergent	No
PTRG_10437	*Ptr*	10433 (*^j^*)				Yes	Yes	No	No	No
PTRG_11759	*Ch*	AAX09988.1	(*^g^*)	Yes	NPS6 – w/o - 3′ (73/84)	No	Yes	No	No	No
PTRG_11809	*Ptr*	10433 (*^j^*)				Yes	Yes	Yes	No	No
PTRG_11818	*Ptr*	11836 (*^j^*)				No	Yes	Yes	No	No
PTRG_11836	*Ptr*	11818 (*^j^*)				No	Yes	Yes	No	No

Ab, *Alternaria brassicae*; Acc., accession number; Cg, *Chaetomium globosum*; Ch, *Cochliobolus heterostrophus*; Cm, *Cochliobolus miyabeanus*; Fi, *Fusarium incarnatum*; Mg, *Magnaporthe grisea*; Org., organism; Pa, *Podospora anserina*; Rec., reciprocal best blast hit; Sn, *Stagonospora nodorum*; SNP, single-nucleotide polymorphism.

a Best hit BlastP (Altschul *et al*. 1997) NCBI.

b SNP predicted in maq.

c Comparison by Needle in EMBOSS (Needleman and Wunsch, 1970, Rice *et al.* 2000); % identity/similarity.

d At least two domains in the protein share >96% similarity.

e Associated with transposable elements (TE).

f Present in putative cluster.

g See Cochliobolus NPS column.

h Other members in cluster have EST support.

i P. Martinez and L. Ciuffetti, unpublished data.

j Apparent duplication in *P. tritici-repentis genome*.

Two additional NRPSs that are conserved in the three *Ptr* isolates, PTRG_09101 and 09808, have homologs in the wheat pathogen *S. nodorum*, one of which appears to be present in a biosynthetic cluster (*PTRG_09101*); however, the function of these NRPSs is unknown. There are also two NRPS:PKS (polyketide synthase) hybrid molecules present in all isolates. *PTRG_00649* encodes an incomplete NRPS module at its N-terminus and a PKS ketoacyl-synthase (KS) domain at its C-terminus. It is most closely related to MGG_07803 of *M. oryzae* with the exception that PTRG_00649 does not contain a full NRPS module. Upstream of this gene is a TE, which may have led to the truncation of the NRPS module or could be the footprint from an HGT event. Unlike *PTRG_00649*, *PTRG_04244* encodes a 5′ PKS module and a complete NRPS module at its 3′ end. *PTRG_04244* is surrounded by a putative biosynthetic cluster (Figure S7), and many of the genes in this cluster have EST support. The *PTRG_04244*-containing cluster encodes several genes similar to those found in the genome of the ascomycete *Talaromyces stipitatus*, a teleomorph of a potent human pathogen, *Penicillium marneffei* (Cooper and Vanittanakom 2008).

**Figure 8:**
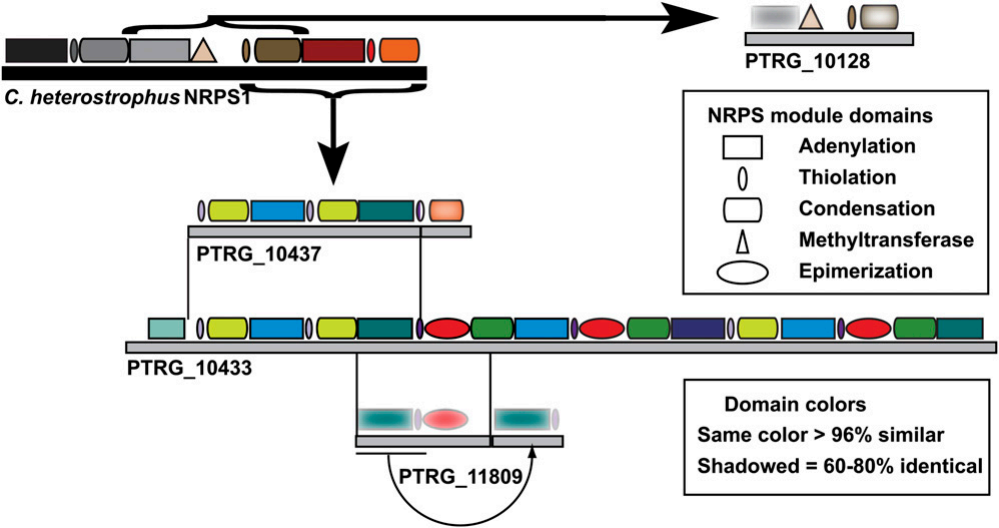
NRPSs diversification in the genome of *Pyrenophora tritici-repentis*. Schematic shows the modular architecture of *Cochliobolus heterostrophus* NRPS1 (black solid bar and associated modules) and related *P. tritici-repentis* PtNRPSs (gray solid bars and associated modules). Brackets with straight arrows define modules in *C. heterostrophus* NRPS1 that have homologous and/or related regions present in PtNRPSs. The module domains (adenylation, thiolation, condensation, methyltransferase, and epimerization), and the solid bars that represent the entire protein are drawn to scale. Thin lines between gray bars extend to the region(s) of identity between proteins and the curved arrow depicts a recent duplication event.

The observation of complicated duplication, recombination and domain swapping of *Ptr* NRPSs in the pathogenic isolates further confirms that TEs play an important role in NRPS diversification in this species (Figure 8). Many of the domains and modules that are being duplicated and recombined are related to those present in *C. heterostrophus* NRPS1 (ChNRPS1). For example *PTRG_10128*, which is orthologous to the central module of ChNRPS1, is surrounded by TEs and remnants of TEs. The putative NRPS, PTRG_10437, is composed of one incomplete (T-C) and two complete modules (A-T-C) that contain domains that are related to the 3′ end of ChNRPS1. Interestingly, the A domain of module 3 of ChNRPS1 and some of the modules present in PTRG_10437 are related to A domains in the NRPS necessary for the production of AM-toxin from *A. alternata* (Bushley and Turgeon 2010). The first incomplete module of PTRG_10437 is > 95% similar to the T-C domains in the second module, suggesting a fairly recent duplication. The A domains in modules two and three do not show that level of conservation and the C domain in the third module is more similar to the last C domain in ChNRPS1 than to any other C domain in the NRPSs of *Ptr*.

This complex level of duplication and domain swapping is even more evident in PTRG_10433, the largest NRPS (8,548 aa) in the *Ptr* genome. This protein appears to be derived from PTRG_10437 and is composed of six complete modules (A-T-C) with an additional A domain at the C-terminus. Two new A domains have been introduced into this protein, one at its N-terminus and one into the fifth module, resulting in modules with unique A domains but practically identical T and C domains. A new T, epimerization and C domain combination has been introduced, duplicated and recombined, in one case creating a unique module. Reads from the pathogenic DW7-ToxB map to these *NPS*s, but it is difficult to determine if the domains and modules are arranged in a similar manner due to the difficulty of assembling highly similar reads. The presence of a similar *NPS* in only the pathogenic isolates is consistent with a previous study in which pathogen-specific probes were isolated using a subtractive approach, and two of these probes contained sequences present in *PTRG_10433* (Lichter *et al.* 2002). There is another locus, *PTRG_11809* that appears to have been derived from *PTRG_10433*; however, neither of the two modules is complete. Whether the *NPS*s in *Ptr* that encode partial modules are functional may not be as important as their role as reservoirs for NRPS diversification.

In addition to domain and module duplication contributing to NRPS diversification, the *Ptr* reference genome also contains duplications (three copies) of an entire chromosomal region that contains not only two *NPS* genes (*PTRG_11818* and *11836*, 97% identical at the amino acid level), but also cytochrome p450, amino transferase and methyltransferase genes, although not all genes are equally maintained in the duplication (Figure S8). The repetition of this region of the genome and the loss of some genes are likely due to transposition events as there are TEs throughout the duplications. Although the function of this NRPS and the surrounding proteins present in this regional duplication is not known, it has been shown in the apple pathotype of *A. alternata* that gene duplication in the biosynthetic cluster responsible for the production of the HST, AM-toxin, is necessary for full pathogenicity (Harimoto *et al.* 2008).

Unique among the three sequenced isolates, the reference genome produces ToxC, which is hypothesized to be the product of a biosynthetic cluster as opposed to a single gene (Effertz *et al.* 2002). Therefore, the BFP-ToxAC strain-specific NRPSs and/or their biosynthetic cluster components are potential candidates for characterizing this HST. Alternatively, ToxC and other currently uncharacterized *Ptr* toxins could be the products of polyketide synthases (PKS). The *Ptr* reference genome contains 21 putative *PKS* loci. However, most of these PKSs are conserved in all of the isolates sequenced (Table S15), thereby reducing the probability that one of them is responsible for the production of the BFP-ToxAC-specific ToxC. (Table S16). *Ptr* is known to produce anthraquinone-derived phytotoxins (Bouras and Strelkov, 2010; Bouras *et al.* 2009) and a biosynthetic cluster similar to the emodin cluster of *Aspergillus nidulans* (Bok *et al.* 2009) was identified in the reference genome. Also found are clusters related to those in other fungi that produce resorcyclic-lactone-based compounds [*i.e.*, zealerone (Lysoe *et al.* 2009; Reeves *et al.* 2008)] and alternapyrone (Fujii *et al.* 2005). The PKS and accessory proteins necessary for melanin biosynthesis (Kihara *et al.* 2008) were also identified (PTRG_03323, PTRG_03315 = T4HN, PTRG_04757 = SCD, and PTRG_03318 = CmrI). Although there is no evidence for duplication and recombination events playing a role in PKS diversification in the pathogens, there are loci that have clearly been truncated when compared to their putative homologs. Additionally, there are two other pathogen-specific PKSs, which are recent duplications and are 100% identical. So although TEs are not necessarily as important for PKS as compared with NRPS evolution in *Ptr*, copy-number variation may contribute to the amount of a product that can accumulate.

### The *P. tritici-repentis* secretome provides insights into a necrotrophic lifestyle

The secretome of plant-pathogenic fungi plays an important role in breaching host defenses, establishing and maintaining growth once the host has been penetrated and, in the case of a necrotrophic pathogen, killing host tissue to obtain nutrients by initiating cell death cascades, often by taking advantage of the host plant immune system (Oliver and Solomon, 2010; Pandelova *et al.* 2009, 2012; Wolpert *et al.* 2002). Therefore, to understand the diseases caused by these pathogens in general, and necrotrophs in particular, it is imperative to analyze the secreted component of the proteome. Although the proteinaceous HSTs of *Ptr* described in the previous section are undoubtedly critical for pathogenesis and virulence, they are only two proteins in what is likely an extensive repertoire of pathogenesis-related secreted proteins important to the *Ptr*-wheat pathosystem. For the purposes of identifying a comprehensive set of putative secreted proteins, we produced a very inclusive secreted protein dataset, at the risk of including some false-positives. Once defined, the secreted proteins were annotated with conserved domains and GO ontology terms.

**Figure 9:**
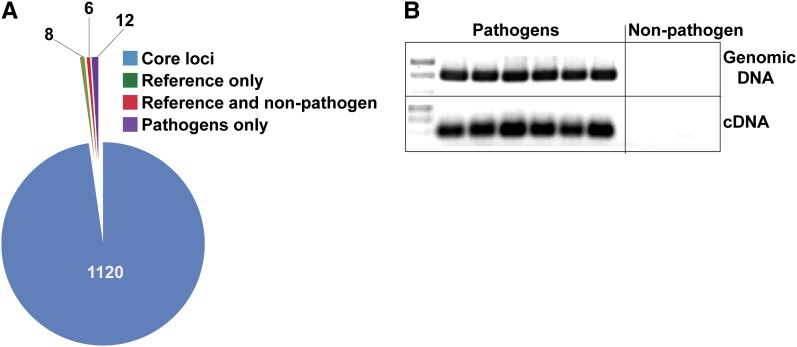
Predicted secreted proteins in the genome sequence of *Pyrenophora tritici-repentis*. (A) Distribution of secreted proteins in the reference *Ptr* isolate (BFP-ToxAC) and the Illumina-sequenced pathogenic (DW7-ToxB) and nonpathogenic (SD20-NP) isolates. (B) PCR screen of pathogenic and nonpathogenic isolates for the presence of the gene and transcript for PTRG_11888, a predicted putative secreted protein. Pathogenic isolates: BFP-ToxAC; ASC1; 86-124; D308; DW2; SO3-P; Non-pathogenic isolates: SD20-NP; 90-2; 98-31-2 (see also Table S1).

We predicted 1146 potential secreted proteins in the reference genome of *Ptr* (Table S14) of which 317 are small (< 200 aa) and 69 of those are cysteine-rich, characteristics typically shared among small effectors (Rep 2005; Stergiopoulos and De Wit 2009). One hundred ten of these proteins had no BLAST hits in the nonredundant database at the NCBI and we consider these to be *Ptr*-specific (Table S17). Comparison with the two Illumina-sequenced isolates (Figure 9A) revealed that 1120 (98%) of the secreted proteins are shared between all isolates, 12 proteins are shared only between the two pathogenic isolates, eight are found only in the reference race 1 isolate, BFP-ToxAC, and six are shared between the reference genome and that of the nonpathogen. Of the eight BFP-ToxAC-specific secreted proteins, only two have informative annotations: the known HST ToxA (PTRG_04889), and a putative endochitinase (PTRG_04903), which is also encoded in the ToxA-containing 145-kb region of the reference genome that is absent in the other two sequenced isolates. A good candidate effector among the eight BFP-ToxAC-specific secreted proteins is a small secreted protein (SSP) of 59 aa and 7% cys content (PTRG_10524). ESTs for three of the 12 pathogen-shared secreted proteins (PTRG_11888, PTRG_11773 and PTRG_12138) were detected in the *in planta* expression library, suggesting their potential contribution to disease development. PCR screening of one of these candidate pathogenicity-related genes, *PTRG_11888*, among five additional pathogenic and two additional non-pathogenic isolates (Table S1), confirmed the association of this locus with pathogenic isolates regardless of race (Figure 9B). Although its function has yet to be determined, this small (85 aa) cysteine-rich (14%) protein encodes a conserved 2Fe-2S ferredoxin, iron-sulfur binding site, indicating a potential function in redox reactions. Among all of the secreted proteins in the reference protein data set there were three main categories of abundant GO/PFAM terms: Cell wall degrading enzymes and lipases, proteins involved in oxidation-reduction reactions, and proteins involved in proteolysis (Table 5).

**Table 5:** The most abundant annotations in the predicted secretome of *Pyrenophora tritici-repentis*

Gene Ontology (GO) and Pfam Terms		
GO ID/Accession No.	GO Term/Accession Description	No. Loci with Term
Molecular function		
GO:0004553	Hydrolase activity, hydrolyzing O-glycosyl compounds	112
GO:0050660	FAD binding	43
GO:0020037	Heme binding	37
GO:0004497	Monooxygenase activity	34
GO:0009055	Electron carrier activity	32
GO:0003676	Nucleic acid binding	18
GO:0016614	Oxidoreductase activity, acting on CH-OH group of donors	18
GO:0004601	Peroxidase activity	16
GO:0004091	Carboxylesterase activity	16
GO:0004180	Carboxypeptidase activity	15
GO:0008270	Zinc ion binding	13
GO:0005515	Protein binding	13
GO:0016810	Hydrolase activity, acting on carbon-nitrogen (but not peptide) bonds	12
GO:0030248	Cellulose binding	12
GO:0004252	Serine-type endopeptidase activity	12
GO:0016829	Lyase activity	12
GO:0008237	Metallopeptidase activity	11
GO:0016757	Transferase activity, transferring glycosyl groups	11
Biological processes		
GO:0055114	Oxidation reduction	65
GO:0006508	Proteolysis	61
GO:0006066	Alcohol metabolic process	24
GO:0044267	Cellular protein metabolic process	18
GO:0051704	Multiorganism process	16
GO:0006629	Lipid metabolic process	16
GO:0051591	Response to cAMP	14
GO:0044262	Cellular carbohydrate metabolic process	14
GO:0009308	Amine metabolic process	14
GO:0034645	Cellular macromolecule biosynthetic process	13
GO:0006139	Nucleobase, nucleoside, nucleotide and nucleic acid metabolic process	13
GO:0043581	Mycelium development	12
GO:0045493	Xylan catabolic process	11
GO:0016998	Cell wall macromolecule catabolic process	11
Pfam domains*^a^*		
PF00067	Cytochrome p450	30
PF03443	Glycosyl hydrolase family 61	26
PF01565	FAD binding domain	25
PF08031	Berberine and berberine-like	21
PF00732	GMC oxidoreductase (binds FAD as a cofactor)	18
PF05199	GMC oxidoreductase	16
PF00187	Carbohydrate Binding Module 18	16
PF00135	Carboxylesterase	15
PF00082	Subtilase	11
PF00734	Carbohydrate Binding Module 1 (cellulose-binding domain)	11
PF04389	Peptidase Family M28 (metalloprotease)	10
PF00657	GDSL family of esterases/lipases	10
PF01083	Cutinase (cutin + H_2_O ↔ cutin monomers)	10

Values represent the lowest node per branch (>10 sequences) of a directed acyclical graph of the hierarchical structure of GO terms.

a e-value cutoff for Pfam analysis E < 0.1.

#### Cell wall-degrading enzymes:

Similar to most plant-pathogenic fungi, *Ptr* encodes a high number and variety of cell wall-degrading enzymes (CWDEs) to degrade host cell walls during infection (Table S18). However, the number of CWDEs encoded in *Ptr* is greater than those encoded by the “stealth pathogen,” *M. graminicola*, another hemibiotrophic Dothideomycete wheat pathogen. The lifestyle of *M. graminicola* differs from that of *Ptr* in that it has a latent biotrophic phase before switching to necrotrophic symptoms (Goodwin *et al.* 2011). In contrast, *Ptr* induces cell death rapidly in susceptible hosts. It is hypothesized that the reduction of CWDEs in *M. graminicola* results in the ability of the pathogen to escape detection by the host. Therefore, the presence of unique expansions and contractions of CWDE families can provide information about the lifestyle of *Ptr*.

The cell walls of grasses are significantly different than those of dicots as they contain, in addition to cellulose, a relatively high percentage of glucuronoarabinoxylans, mixed-linkage glucans, and a relatively low percentage of xyloglucan, mannans and glucomannans, pectin and structural proteins (Vogel 2008). Consistent with a lifestyle as a wheat pathogen, we find that the *Ptr* genome encodes fewer pectin-degrading enzymes, including those in the polysaccharide lyase and GH (glycosyl hydrolase) families 28, 78, 88, 95, compared with the dicot pathogens *Verticillium dahliae* (Klosterman *et al.* 2011) and *F. oxysporum* (Table S19) (Ma *et al.* 2010). Furthermore, there are at least three putative mixed-linkage glucanases that would aid in cell wall degradation in grasses, PTRG_07078 (GH16) and PTRG_06274 (GH12), orthologs of Mlg1 and Mlg2 of the maize pathogen *C. carbonum*, and PTRG_03370 (GH16) (Kim *et al.* 2001). *PTRG_06274* is one of the loci represented in a GO enrichment analysis of the *in planta* EST library, suggesting its importance in the pathogenesis process (Table S20).

In the GO enrichment analysis, the endoglucanases represented the largest group (six) of cellulose-degrading enzymes, with expression of four of these genes (*PTRG_04829*, *06274*, *04285*, *03392* and their families GH45, GH12, GH5, and GH61/CBM1, respectively) found exclusively in the *in planta* EST library. The cellobiohydrolase/exoglucanase PTRG_09037 (GH7), the ortholog of Cel1 of *C. carbonum* (Sposato *et al.* 1995) and CbhA of *A. nidulans* (Gielkens *et al.* 1999), had the greatest number of transcripts detected in the *in planta* library of any of the secreted proteins. Glucuronoarabinoxylans is the major hemicellulose in grass cell walls and degradation of the xylose backbone requires the production of xylanases (Aro *et al.* 2005; De Vries and Visser 2001). PTRG_08873, 09530, and 10207, orthologs of *C. carbonum* Xyl1 (GH11), Xyl4 (GH10) and Xyl3 (GH11), respectively, were detected in the *in planta* EST library with only PTRG_10207 detected both *in planta* and in culture. Xylanases, as well as other CWDEs appear to be functionally redundant in *C. carbonum* and it has been hypothesized that the differential expression of these genes may reflect a need for redundancy (Apel-Birkhold and Walton 1996); this may also be true for *Ptr* where CWDEs also appear to be differentially expressed.

Unexpectedly, *Ptr* encodes fewer proteins in the CBM family of CWDE in comparison to most of the other fungi examined in this study, be they plant pathogenic or not, although not as few as in *M. graminicola* (Table S21). Most of this decrease is due to fewer proteins in the CBM18 family, predicted to be fungal chitinases. Fungal chitinases can be grouped into three subgroups, sg A, sg B, and sg C, with sg C containing either CBM18 or CBM50 (LysM) domains (Hartl *et al.* 2012). Although functions of proteins in these families are not well understood, expression studies of sg C genes in the mycoparasitic fungi *Trichoderma virens* and *T. atroviride* suggest roles in morphogenetic development and exogenous chitin degradation (Gruber *et al.* 2011; Gruber and Seidl-Seiboth 2012).

#### Proteins involved in detoxifying redox reactions—cytochrome p450s, berberine bridge enzymes, and other oxidative-stress-related proteins:

Plant responses to invasion include the formation of reactive oxygen species, the production of secondary metabolites, and the secretion of defense-related proteins. Fungi must deal with all of these components if they are to survive. The secreted proteins of *Ptr* reflect the need to protect itself from plant-produced compounds.

A surprising number of proteins (29) containing conserved cytochrome p450 domains (p450) were present in our secreted protein dataset. This may be due to the retention of a subset of proteins with one TM domain. When we remove those p450s that contain predicted TM domains, there are still 12 loci remaining in the dataset. The secretion of these p450s must be experimentally validated, but it suggests a possible method by which *Ptr* could persist within the host prior to induction of cell death by neutralizing plant-produced anti-fungal compounds. The best example of a p450 involved in this type of interaction is pisatin demethylase, produced by the pea pathogen *Nectria hematococca*, which neutralizes the pea phytoalexin pisatin (Maloney and VanEtten 1994). Other preformed secondary metabolites (Imperato and Pandalai 2006), as well as phenolic compounds produced in response to *Ptr* (Dushnicky *et al.* 1998a,b; Pandelova *et al.* 2009, 2012), also might be a target for these p450s.

There are 21 proteins predicted in the *Ptr* secretome that contain a combination of FAD-binding and berberine or berberine-like domains. Flavoenzymes with this combination of domains are known as berberine bridge enzymes (BBE), some of which are produced in response to plant pathogens, and have been shown to be important for the production of plant alkaloids (Dittrich and Kutchan 1991; Facchini *et al.* 1996) and carbohydrate oxidases (Carter and Thornburg 2004; Custers *et al.* 2004), and the generation of hydrogen peroxide. BBEs also are over-represented in the secretome of the oomycete *P. infestans* (Raffaele *et al.* 2010), where they may contribute to the production of reactive oxygen species and alkaloid modification during *P. infestans* infection. TEs also are playing a role in the expansion of BBE-family proteins in *Ptr*. A segment of the genome containing *PTRG_11734*, a BBE, and *PTRG_11735* is surrounded by TEs and a duplication of this segment (96.4% identical at the nucleotide level), also surrounded by TEs, is present in the genome (*PTRG_02303* = *PTRG_11734* and *PTRG_02304* = *PTRG_11735*).

Other dominant Pfams suggest that proteins important for oxidation-reduction reactions are predominant in the secretome; these include glucose−methanol−choline oxidoreductase domains (18 loci). Although these enzymes appear to have multiple catalytic functions (Cavener 1992), little is known about their impact in plant host-pathogen interactions. It has recently been proposed that a glucose−methanol−choline oxidoreductase produced by *Glomerella cingulata* plays a role in plant pathogenicity through the reduction of pathogen-induced quinones, which have anti-fungal activities, and phenoxy radicals produced for reinforcement of plant cells walls during pathogen attack (Sygmund *et al.* 2011).

### Summary

The era of genome sequencing has provided us with incredible resources to answer questions and pose new hypotheses about what contributes to the pathogenicity of microorganisms and the host responses that govern resistance or susceptibility. Sequencing of multiple isolates of *Ptr*, including two pathogenic and one non-pathogenic isolate, has provided ample evidence that the establishment of pathogenicity in this system, and possibly host switching from wild grasses (nonpathogens) to wheat (pathogens), likely started with structural changes to the genome. These changes were facilitated by the influx of TEs that resulted in increased genetic plasticity and thereby likely also reduced the possibility of gene flow between pathogenic and non-pathogenic isolates. In addition, TEs are probably one of the major factors that contribute to the high genetic diversity of the pathogen population. Such dynamic changes enabled the introduction, amplification, and diversification of various toxin genes and pathogenicity factors and created the complex race structure that this organism displays today. Many of these TEs appear to be active, which contributes to a genomic landscape in the pathogen population that is capable of rapid adaptation. These findings are consistent with the “jump or die model” for the macroevolutionary persistence of host-specialized filamentous fungi proposed by Raffaele and Kamoun (2012). In this model, pathogen lineages with highly adaptable genomes are better able to jump to new hosts than those with less flexible genomes, and therefore, are more likely to persist over evolutionary time when host extinction or complete resistance would otherwise result in pathogen extinction.

Highly adaptable genomes often have relatively high amounts of repetitive DNA (Raffaele and Kamoun 2012), which may result in expansion of genome size; for instance, biotrophic- and oomycete-plant pathogens have some of the largest genome sizes and repetitive DNA content (Haas *et al.* 2009; Spanu *et al.* 2010). Though the size of the *Ptr* genome and the amount of repetitive DNA falls into the range of that of other Dothideomycete-plant pathogens (Goodwin *et al.* 2011; Hane *et al.* 2007; Rouxel *et al.* 2011), nonpathogenic *Ptr* isolates tend to have smaller genome sizes and the isolates sequenced in this study indicate a higher repetitive DNA content in the pathogenic isolates. The expansion of TEs in the pathogenic *Ptr* isolates appears to be an ongoing event, as members of a single repeat family often share identical or very similar sequences. The low amount of typical repeat-induced point mutations in these highly similar repeat sequences suggests that RIP, if occurring at all, is inefficient in *Ptr*. The lack of RIP has been postulated to be one of the drivers in the expansion of genome size in the powdery mildew fungi (Spanu *et al.* 2010) and may also explain genome expansion in pathogenic isolates of *Ptr*.

Many of the events that contribute to genome plasticity also contribute to changes in pathogen virulence. Recombination can lead to domain swapping between virulence proteins as has been shown in the oomycete Crinkler family of modular proteins, which exhibit domain swapping and chimera formation (Haas *et al.* 2009), and in the NRPSs of *Ptr*. TE-mediated duplications can lead to copy number variation (CNV), which in *Phytophthora sojae* has been associated with silencing of effectors (Qutob *et al.* 2009) and in *P. infestans* has been proposed to contribute to the assembly of novel genes (Jiang *et al.* 2006). In *Ptr*, CNV of the HST gene, *ToxB*, correlates with isolate virulence. Additional loci in the *Ptr* genome display CNV; however, their role in virulence has yet to be determined.

HGT is another mechanism that can alter the plant-pathogen genome landscape and contribute to virulence. Entire chromosomes can be transferred between fungi that alter pathogenicity or host-specificity (Coleman *et al.* 2009; Ma *et al.* 2010). Proposed gene transfer events that would alter virulence include the transfer of HSTs, as in the case of *ToxA* in *Ptr* and AAL toxin in *Alternaria alternata* (Akagi *et al.* 2009). Whether genome flexibility is a pre-requisite for efficient HGT is currently unknown.

And what are the implications of transduplication in *Ptr*? Aside from changes normally associated with transposition, capture of genes and gene fragments and their deposition throughout the genome increases the chances of novel gene formation and may very well play important roles in cellular gene expression. Discovery of this phenomenon in *Ptr* allows us to ask some interesting questions. Is there selective pressure for the gene/gene fragments that are captured; and if so, why? Do they play a role in development or pathogenicity? Clearly, there is an increased copy number and expression of a histone H3 variant, which might very well have a significant impact on genome structure and function as histones are essential components of chromosomes. Could altered chromatin be in part responsible for the recent amplification of transposable elements, segments of the genome, and the unique recombination events we see in the pathogen? Can this unusual histone H3 provide a tool for better understanding of chromatin structure and function? Implementation of novel strategies, computational and experimental approaches will be required to answer these fundamental questions.

## Supplementary Material

Supporting Information
