# Supplementary material for: Comparative Genomics of a Plant-Pathogenic Fungus, Pyrenophora tritici-repentis, Reveals Transduplication and the Impact of Repeat Elements on Pathogenicity and Population Divergence
Source: G3 (Bethesda). 2013 Jan 1;3(1):41–63. doi: 10.1534/g3.112.004044 (PMC3538342; doi:10.1534/g3.112.004044)
Supplement: Supporting Information [file supp_3_1_41__index.html]

Supporting Information 

# Comparative Genomics of a Plant-Pathogenic Fungus, *Pyrenophora tritici-repentis*, Reveals Transduplication and the Impact of Repeat Elements on Pathogenicity and Population Divergence

## Supporting Information for Manning *et al.*, 2013

**Files in this Data Supplement:**

- Supporting Information - Figures S1-S8 and Tables S1-S21 (PDF, 3.3 MB)
- Figure S1 - Similarity and distribution of (A) LTR retrotransposons, (B) DNA transposons and (C) MITES across the Supercontig assemblies of BFP-ToxAC (PDF, 1.6 MB)
- Figure S2 - Heat map of Pfam domains for (A) carbohydrate active enzymes and other cell wall enzymes, (B) proteins involved in sensing and response (C) proteins associated with transport, oxidative stress and toxin activity present in *Ptr* and other cereal pathogens (PDF, 340 KB)
- Figure S3 - Alignment of novel H3-like (H3L) genes identified in three different *Ptr* isolates (PTRG numbers refer to genes identified in the reference genome of BFP-ToxAC, while DW7 and SD20 refer to the pathogenic DW7-ToxB and non-pathogenic SD20-NP strains, respectively) (PDF, 97 KB)
- Figure S4 - Alignment of *bona fide* histone H3 proteins from *Ptr* with those of other fungi reveal the expected level of conservation (PDF, 168 KB)
- Figure S5 - Transduplication of osmosensory transporter coiled-coil (Osmo-CC) domain in *Ptr* (PDF, 259 KB)
- Figure S6 - Modular architecture and biosynthetic cluster of a putative histone deacetylase inhibitor biosynthetic cluster in *P. tritici-repentis* (PDF, 414 KB)
- Figure S7 - Modular architecture and biosynthetic cluster of a *P. tritici-repentis*-specific PKS/NRPS hybrid (PDF, 332 KB)
- Figure S8 - Duplicated genomic regions associated with the NRPSs PTRG\_11818 & 11836 (PDF, 370 KB)
- Table S1 - *P. tritici-repentis* isolates used in this study (PDF, 81 KB)
- Table S2 - *P. tritici-repentis* reference genome libraries sequenced(PDF, 73 KB)
- Table S3 - *P. tritici-repentis* assembly anchored to the optical maps (PDF, 74 KB)
- Table S4 - Illumina sequenced *P. tritici-repentis* isolate read alignment under different stringencies (PDF, 75 KB)
- Table S6 - EST libraries produced to facilitate gene calling (PDF, 70 KB)
- Table S7 - Serine codon usage in *P. tritici-repentis* (PDF, 73 KB)
- Table S8 - Top five repeat familes shared between the *P. tritici-repentis* reference genome and resequenced pathogenic and non-pathogenic isolates (PDF, 75 KB)
- Table S9 - Count of identical 16-mers in 1X sampling of resequencing reads of DW7-ToxB and SD20-NP (PDF, 76 KB)
- Table S12 - Pfam domains in repeats (PDF, 65 KB)
- Table S13 - *De novo* assembly of resequenced *P. tritici-repentis* isolates (PDF, 73 KB)
- Table S15 - Putative NRPS-containing clusters in the *P. tritici-repentis* reference genome (PDF, 85 KB)
- Table S17 - *Pyrenophora tritici-repentis*-specific secreted proteins as predicted in Blast2GO (PDF, 102 KB)
- Table S18 - Total numbers of predicted CAZymes in *PTR* and selected ascomycetes (PDF, 75 KB)
- Table S19 - Comparison of pectinolytic enzymes in *P. tritici-repentis* vs. other grass and non-grass plant pathogens (PDF, 78 KB)
- Table S20 - GO enrichment analysis of ESTs present in the in planta library (PDF, 67 KB)
- Table S21 - Comparison of CBM-containing protein families in *P. tritici-repentis* and other grass and non-grass plant pathogens (PDF, 79 KB)
- Table S5 - Repeat families in *P. tritici-repentis* (.xlsx, 63 KB)
- Table S10 - Histone genes present in *P. tritici-repentis* (.xlsx, 14 KB)
- Table S11 - Histone genes present in *Puccinia graminis* (.xlsx, 50 KB)
- Table S14 - Predicted secretome of *P. tritici-repentis* (.xlsx, 197 KB)
- Table S16 - Putative polyketide synthetase gene of *P. tritici-repentis* (.xlsx, 47 KB)
