# Supplementary material for: Comparative Genomics of a Plant-Pathogenic Fungus, Pyrenophora tritici-repentis, Reveals Transduplication and the Impact of Repeat Elements on Pathogenicity and Population Divergence
Source: G3 (Bethesda). 2013 Jan 1;3(1):41–63. doi: 10.1534/g3.112.004044 (PMC3538342; doi:10.1534/g3.112.004044)
Supplement: Supporting Information [file supp_3.1.41_004044SI.pdf]

A

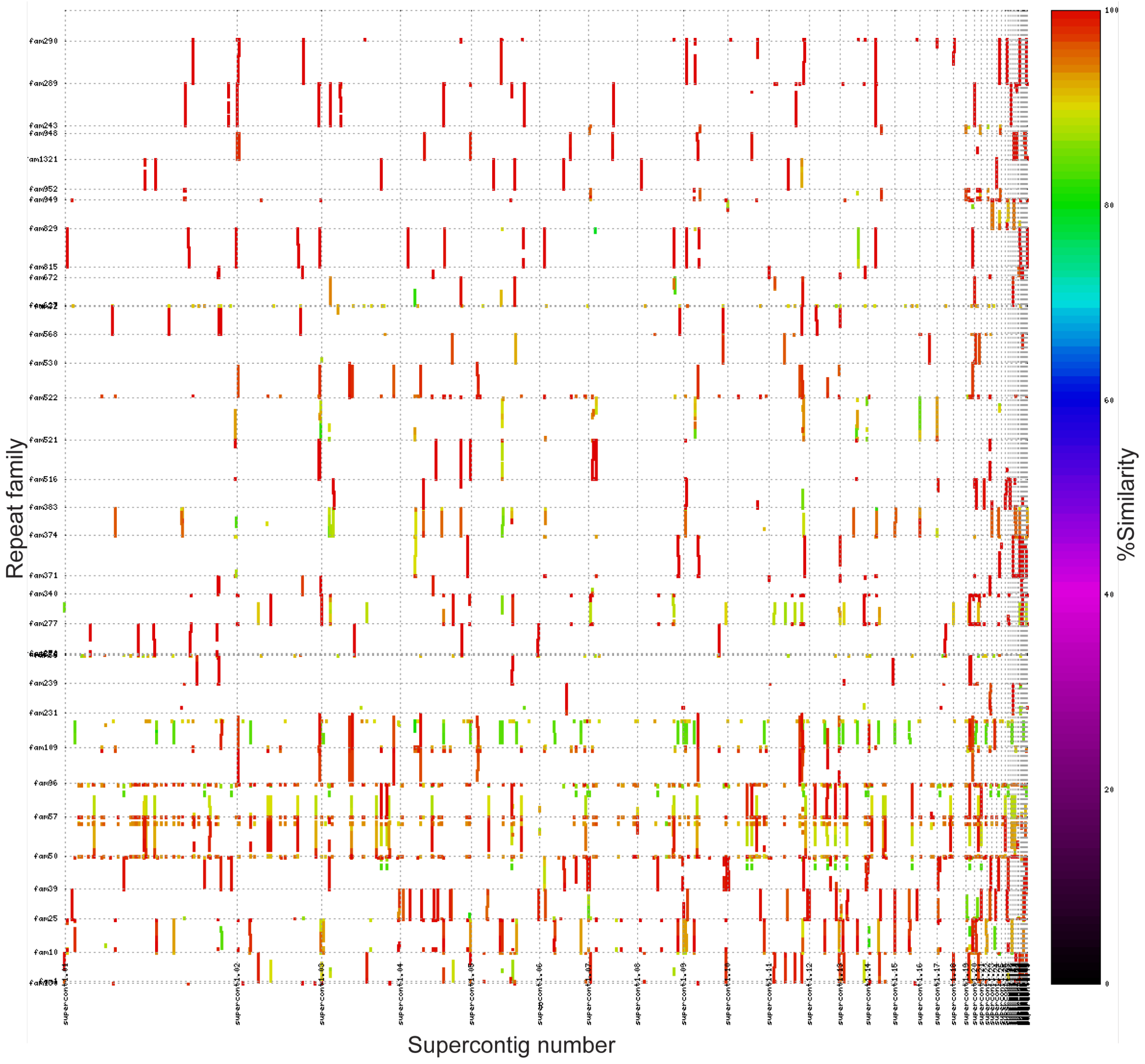

B

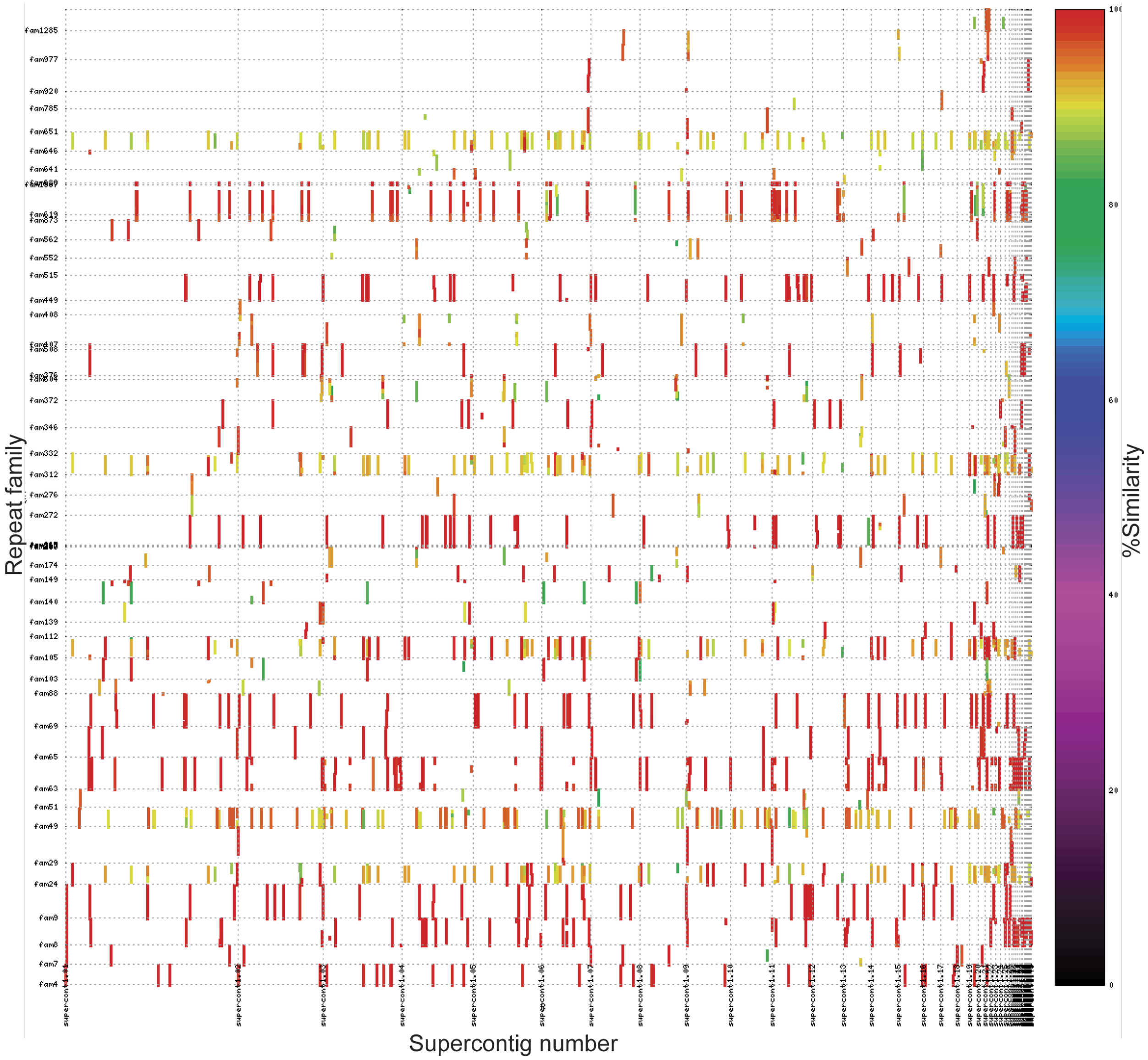
Repeat family
Supercontig number
%Similarity
100
80
60
40
20
0

C

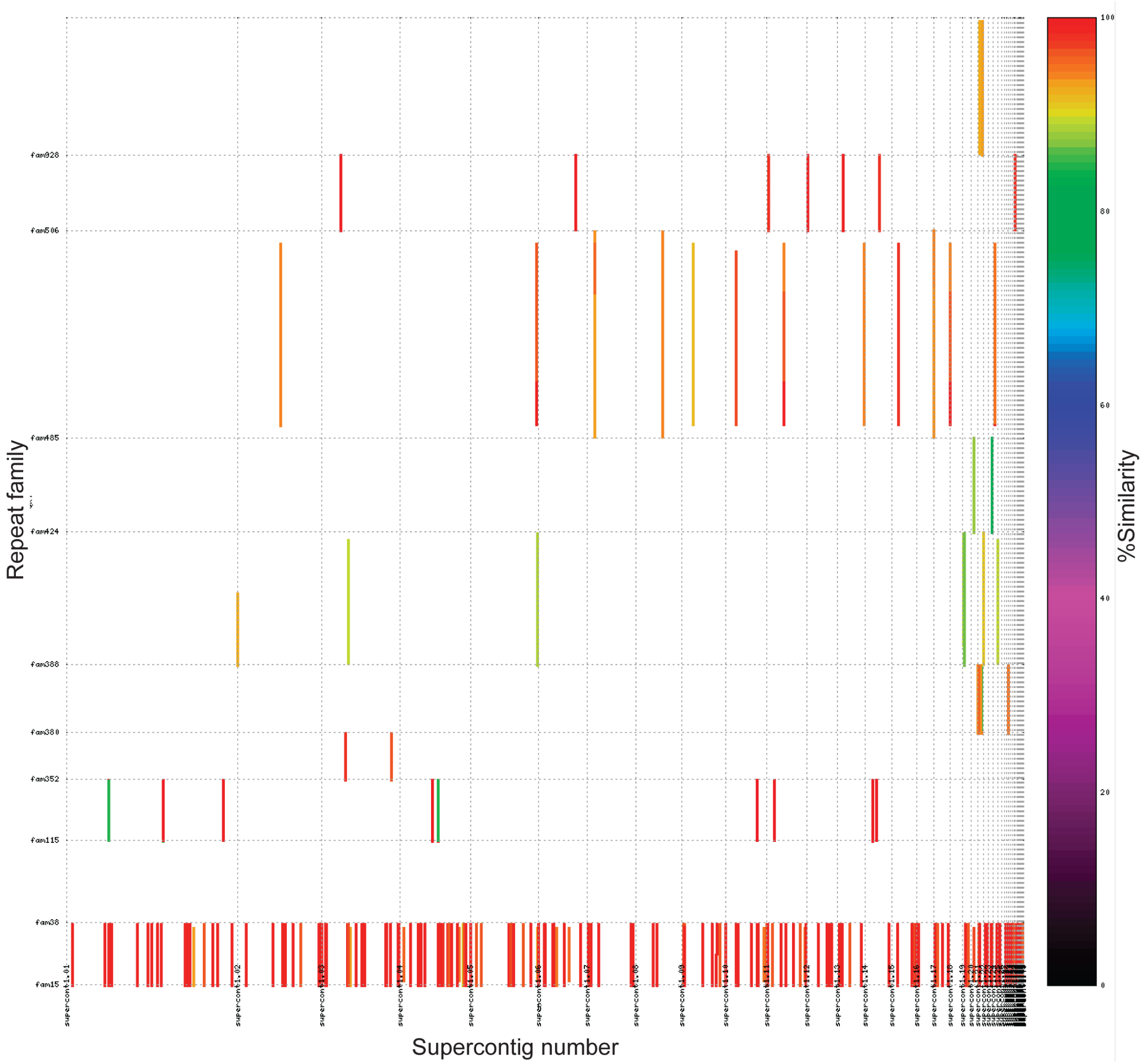

Figure S1 Similarity and distribution of (A) LTR retrotransposons, (B**)** DNA transposons and (C) MITES across the Supercontig assemblies of BFP-ToxAC. The repeat family name is indicated on the left. The color scale on the right indicates % similarity from 0 (black) to 100 (red).

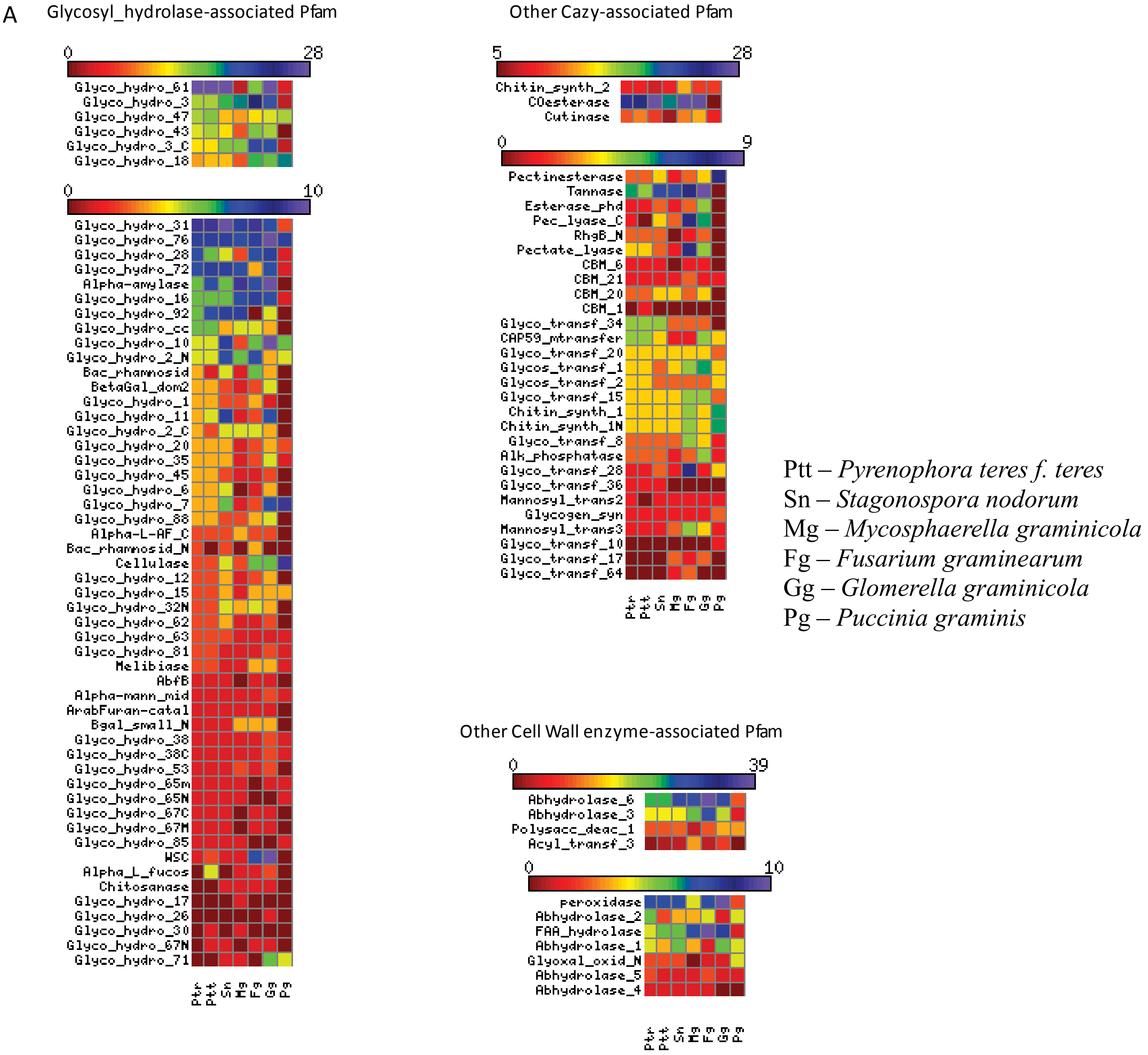
A
Glycosyl_hydrolase-associated Pfam
0
28
Glyco_hydro_61
Glyco_hydro_3
Glyco_hydro_47
Glyco_hydro_43
Glyco_hydro_3_C
Glyco_hydro_18
0
10
Glyco_hydro_31
Glyco_hydro_76
Glyco_hydro_28
Glyco_hydro_72
Alpha-amylase
Glyco_hydro_16
Glyco_hydro_92
Glyco_hydro_cc
Glyco_hydro_10
Glyco_hydro_2_N
Bac_rhamnosid
BetaGal_dom2
Glyco_hydro_1
Glyco_hydro_11
Glyco_hydro_2_C
Glyco_hydro_20
Glyco_hydro_35
Glyco_hydro_45
Glyco_hydro_6
Glyco_hydro_7
Glyco_hydro_88
Alpha-L-AF_C
Bac_rhamnosid_N
Cellulase
Glyco_hydro_12
Glyco_hydro_15
Glyco_hydro_32N
Glyco_hydro_62
Glyco_hydro_63
Glyco_hydro_81
Melibiase
AbfB
Alpha-mann_mid
ArabFuran-catal
Bgal_small_N
Glyco_hydro_38
Glyco_hydro_38C
Glyco_hydro_53
Glyco_hydro_65m
Glyco_hydro_65N
Glyco_hydro_67C
Glyco_hydro_67M
Glyco_hydro_85
WSC
Alpha_L_fucos
Chitosanase
Glyco_hydro_17
Glyco_hydro_26
Glyco_hydro_30
Glyco_hydro_67N
Glyco_hydro_71
Ptr Ptt Sn Mg Fg Gg Pg
Other Cazy-associated Pfam
5
28
Chitin_synth_2
COesterase
Cutinase
0
9
Pectinesterase
Tannase
Esterase_phd
Pec_lyase_C
RhgB_N
Pectate_lyase
CBM_6
CBM_21
CBM_20
CBM_1
Glyco_transf_34
CAP59_mtransfer
Glyco_transf_20
Glycos_transf_1
Glycos_transf_2
Glyco_transf_15
Chitin_synth_1
Chitin_synth_1N
Glyco_transf_8
Alk_phosphatase
Glyco_transf_28
Glyco_transf_36
Mannosyl_trans2
Glycogen_syn
Mannosyl_trans3
Glyco_transf_10
Glyco_transf_17
Glyco_transf_64
Ptr Ptt Sn Mg Fg Gg Pg
Ptt – Pyrenophora teres f. teres
Sn – Stagonospora nodorum
Mg – Mycosphaerella graminicola
Fg – Fusarium graminearum
Gg – Glomerella graminicola
Pg – Puccinia graminis
Other Cell Wall enzyme-associated Pfam
0
39
Abhydrolase_6
Abhydrolase_3
Polysacc_deac_1
Acyl_transf_3
0
10
peroxidase
Abhydrolase_2
FAA_hydrolase
Abhydrolase_1
Glyoxal_oxid_N
Abhydrolase_5
Abhydrolase_4
Ptr Ptt Sn Mg Fg Gg Pg

B

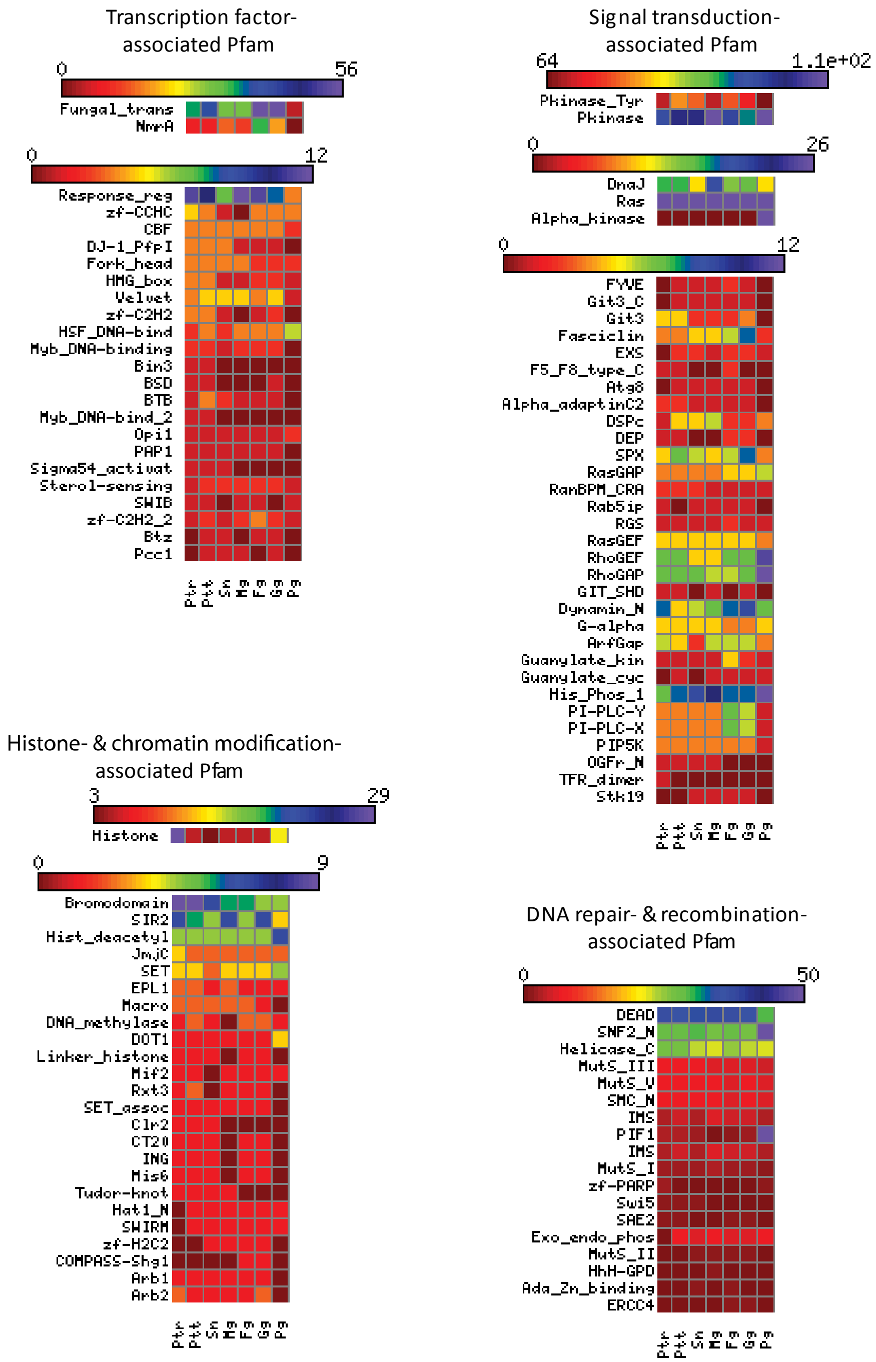

Transcription factor-associated Pfam
0
56
Fungal_trans
NmrA
0
12
Response_reg
zf-CCHC
CBF
DJ-1_PfpI
Fork_head
HMG_box
Velvet
zf-C2H2
HSF_DNA-bind
Myb_DNA-binding
Bin3
BSD
BTB
Myb_DNA-bind_2
Opi1
PAP1
Sigma54_activat
Sterol-sensing
SWIB
zf-C2H2_2
Btz
Pcc1
Ptr Ptt Sn Mg Fg Gg Pg
Signal transduction-associated Pfam
64
1.1e+02
Pkinase_Tyr
Pkinase
0
26
DnaJ
Ras
Alpha_kinase
0
12
FYVE
Git3_C
Git3
Fasciclin
EXS
F5_F8_type_C
Atg8
Alpha_adaptinC2
DSPc
DEP
SPX
RasGAP
RanBPM_CRA
Rab5ip
RGS
RasGEF
RhoGEF
RhoGAP
GIT_SHD
Dynamin_N
G-alpha
ArfGap
Guanylate_kin
Guanylate_cyc
His_Phos_1
PI-PLC-Y
PI-PLC-X
PIP5K
OGFr_N
TFR_dimer
Stk19
Ptr Ptt Sn Mg Fg Gg Pg
Histone- & chromatin modification-associated Pfam
3
29
Histone
0
9
Bromodomain
SIR2
Hist_deacetyl
JmjC
SET
EPL1
Macro
DNA_methylase
DOT1
Linker_histone
Mif2
Rxt3
SET_assoc
Clr2
CT20
ING
Mis6
Tudor-knot
Hat1_N
SWIRM
zf-H2C2
COMPASS-Shg1
Arb1
Arb2
Ptr Ptt Sn Mg Fg Gg Pg
DNA repair- & recombination-associated Pfam
0
50
DEAD
SNF2_N
Helicase_C
MutS_III
MutS_V
SMC_N
IMS
PIF1
IMS
MutS_I
zf-PARP
Swi5
SAE2
Exo_endo_phos
MutS_II
HhH-GPD
Ada_Zn_binding
ERCC4
Ptr Ptt Sn Mg Fg Gg Pg

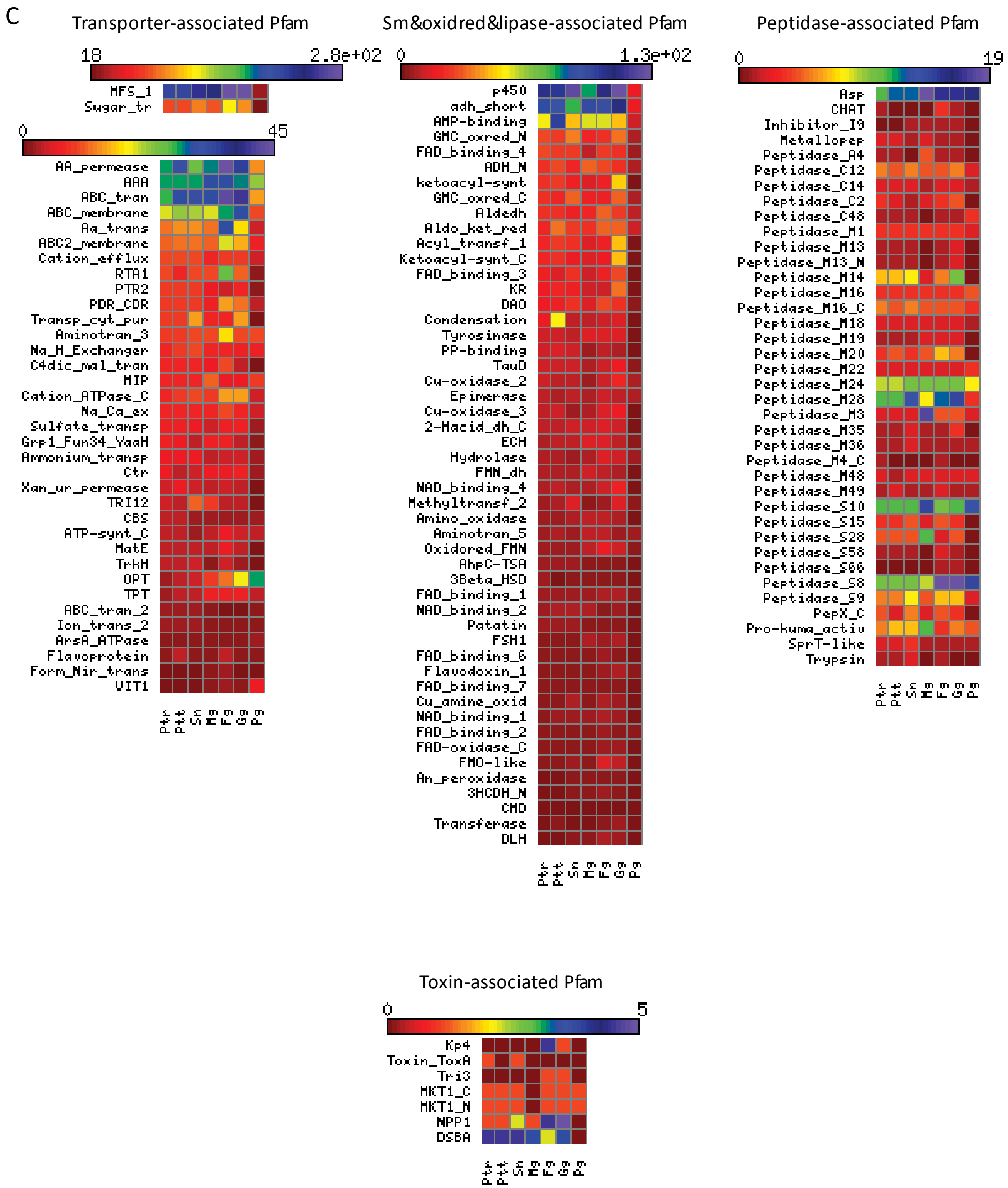

Figure S2 Heat map of Pfam domains for (A) carbohydrate active enzymes and other cell wall enzymes, (B) proteins involved in sensing and response (C) proteins associated with transport, oxidative stress and toxin activity present in *Ptr* and other cereal pathogens.

```
PTRG_03457    CGTCATGCAGTCCGTGCGCTGCTGCCTTTACTAGTGCGGAAATCGGGAAATTGCCGTCGCCCAAACTTTCCAGCTACCTAACTCTTTCAGCCTACTACTT
DW7_94        CGTCATGCAGTCCGTGCGCTGCTGCCTTTACTAGTGCGGAAATCGGGAAATTGCCGTCGCCCAAACTTTCCAGCTACCTAACTCTTTCAGCCTACTACTT
DW7_14025     CGTTATGCAGTCCGTGCGCTGCTGCCTTTACTAGTGCGGAAATCGGGAAATTGCCGTCGCCCAAACTTTCCAGCTACCTAACTCTTTCAGCCTACTACTT
PTRG_00559                                                                           AGCTACCTAACTCTTTCAGCCTACTACTT
SD20_20175                                                                                       CTTTCAGCCTACTACTT

DW7_94        TCTTAATTGTCGACCACATTTATCACAGTAATTAATCACATACGTCGCCTACGTTCATCATCTACTCTACTACGCAATGGCAAGGACAAAACCACGGCCT
DW7_14025     TCTTAATTGTCGACCACATTGATTACAGTAATTAATTACATACGTCGCCTACGTTTATCATCTACTCCACTACGCAATAGCAAGGACAAAACCACGGCCT
PTRG_00559    TCTTAATTGTCGACCACATTTATCACAGTAATTAATCACATACGTCGCCTACGTTCATCATCTACTCTACTACGCAATGGCAAGGACAAAACCACGGCCT
SD20_20175    TCTTAATTGTCGACCACATTTATTACAGTAATTAATTACATACGTCGCCTACATTTATCATCTACTCTACTACGCAATAGCAAGGACAAAACCACGGCCT
PTRG_03457    TCTTAATTGTCGACCACATTTATCACAGTAATTAATCACATACGTCGCCTACGTTCATCATCTACTCTACTACGCAATGGCAAGGACAAAACCACGGCCT

DW_94         AAGGTCACCGGTAAAGCCG
DW_14025      AAGGTCACCGGTAAAGCCG
PTRG_00559    AAGGTCACCGGTAAAGCCGGCCGGGGTGGTCCTGATGGCAAGACAGTTACTGGCGGCAAGCCGGCTCAGAAGGCCCTTGCTAGTAAGGCTAGACGTCAAG
SD20_20175    AAGGTCACCGGTAAAGCCGGCCGGGGTGGTCCTGATGGCAAGACAGTTACTGGCGGCAAGCCGGCTCAGAAGGCCCTTGCTAGTAAGGCTAGACGTCAAG
PTRG_03457    AAGGTCACCGGTAAAGCCGGCCGGGGTGGTCCTGATGGCAAGACAGTTACTGGCGGCAAGCCGGCTCAGAAGGCCCTTGCTAGTAAGGCTAGACGTCAAG

PTRG_00559    TAGCAGGAAAGTCTACGCGAAAGGCACCTGTAGCTGTTAAGAAGAAGCGCAAGTTTAAGGCCGGCAgtaggttatcacagtcgtttatcacagctatttt
SD20_20175    TAGCAGGAAAGTCTACGCGAAAGGCACCTGTAGCTGTTAAGAAGAAGCGCAAGTTTAAGGCCGGCAgtaggttatcacagttgtttattacagctatttt
DW7_11351                                              AAGAAGCGCAAGTTTAAGGCCGGCAgtaggttatcacagttgtttattacagctatttt
PTRG_03457    TAGCAGGAAAGTCTACGCGAAAGGCACCTGTAGCTGTTAAGAAGAAGCGCAAGTTTAAGGCCGGCAgtaggttatcacagtcgtttatcacagctatttt

PTRG_00559    ctaatttagcttagCTGTCGCATTACGGGAAATCAAGAGATACCAGAGAGGTTTTGAACTACTCTTGCGAAAACTCCCCTTTTCCCGCGTAGTGCGCGAA
SD20_20175    ctagataagcttagCTGTCGCATTACGGGAAATTAAGAGATACCAGAGAGGTTTTAAACTACTCTTGCGAAAACTCCCCTTTTCCCGCGTAGTGCGCGAA
DW7_11351     ctaatcaagcttagCTGTCGCATTACTGAAAATTAAGAGATACCAGAGAGGTTTTAAACTACTCTTGCGAAAACTCCCCTTTTCCCGCGTAGTGCGCGAA
PTRG_03457    ctaatttagcttagCTGTCGCATTACGGGAAATCAAGAGATACCAGAGAGGTTTTGAACTACTCTTGCGAAAACTCCCCTTTTCCCGCGTAGTGCGCGAA

PTRG_00559    TTTGCACAGGTGCACAAGGCCGATATCCGCTTTCAACGATCTGCAATCGAAGCTCTTCAGGAAGCTACAGAAGCTTTCTTAGTTGGTTATTTTgagggta
SD20_20175    TTTGCACAGGTGCACAAGGCCGATATCCGCTTTCAACGATCTGCAATCGAAGCTCTTCAGGAAGCTACAGAAGCTTTCTTAGTTACTTATTTTaagggta
DW7_11351     TTTGCACAGGTGCACAAGGCCGATATCCGCTTTTAACGATCTGCAATCGAAGCTCTTCAGGAAGCTACATAAGCTTTCTTAGTTAGTTATTTTaagggta
PTRG_03457    TT-GCACAGGTGCACAAGGCCGATATCCGCTTTCAACGATCTGCAATCGAAGCTCTTCAGGAAGCTACAGAAGCTTTCTTAGTTGGTTATTTTgagggta

PTRG_00559    tagtctatctaccctatactatttttcatttaataacttttatagACTGCAACATCAATGCTATTCACGCAAAGAGGGTTACTATTCAAGAGAAGGATTC
SD20_20175    taatctatctaccctatactatttttcatttaataacttttatagACTGCAACATCAATGCTATTCACGCAAAGAGGGTTACTATTCAAGAGAAGGATTC
DW7_11351     tgatctatctatcctatactatttttcatttaataacttttatagACTGCAACATCAATGCTATTCACGCAAAGAGGGTTACTATTCAAGAGAAGGATTC
PTRG_03457    tagtctatctaccctatactatttttcatttaataacttttatagACTGCAACATCAATGCTATTCACGCAAAGAGGGTTACTATTCAAGAGAAGGATTC

PTRG_00559    TCAATTGGCTAGGCGCTACTTTGCGCGCGAGTTACTAGCTTTTCTCTAGATTATAAGATATATCTAGATACACGTGCTTAAATGCTAGCTGGCGAAGCCT
SD20_20175    TCAATTGGCTAGGCGCTACTTTGCGCGCGAGTTACTAGCTTTTCTCTAGATTATAAGATATATCTAGATACACGTGCTTAAATGCTAGCTGGCGAAGCCT
DW7_11351     TCAATTAGCTAGGCGCTACTTTGCGCGCGAGTTAATAGCTTTTCTCTAGATTATAAGATATATCTAGATACATGTGCTTAAATGGTAGCTGGCGAAGCCT
PTRG_03457    TCAATTGGCTAGGCGCTACTTTGCGCGCGAGTTACTAGCTTTTCTCTAGATTATAAGATATATCTAGATACACGTGCTTAAATGCTAGCTGGCGAAGCCT

PTRG_00559    CTGTATATTATAAATGAAACTAGTTAATGCTACACTG
SD20_20175    CTGTATATTATAAATGAAACTAGTTAATGCTACACTGTGTTAATCTACTG
DW7_11351     CTGTATAGTATAAATGAAACTAGTTAATGCTACACTGTAATAATCTACTGTGATACTATATAAACTTTTCTTTTATTGAAATCTATATTATATATCTTGC
PTRG_03457    CTGTATATTATAAATGAAACTAGTTAATGCTACACTG
```

Figure S3 Alignment of novel H3-like (H3L) genes identified in three different *Ptr* isolates (PTRG numbers refer to genes identified in the reference genome of BFP-ToxAC, while DW7 and SD20 refer to the pathogenic DW7-ToxB and non-pathogenic SD20-NP strains, respectively). In all cases, contigs assembled *de novo* from Illumina reads suggest that copies of H3L genes in both DW7-ToxB and SD20-NP are inactive (note the many point mutations when compared to the PTRG sequences). DW7_14025 and SD20_20175 in particular lack a proper start codon (ATG to ATA change, underlined), while DW7_11351 contains several in-frame nonsense codons in addition to the conserved stop codon (underlined).

A

```
Ptr H3           1 MARTKQTARKSTGGKAPRKQLASKAARKSAPSTGGVKKPHRYKPGTVALREIRRYQKSTE
Sn H3            1 MARTKQTARKSTGGKAPRKQLASKAARKSAPSTGGVKKPHRYKPGTVALREIRRYQKSTE
NcH3             1 MARTKQTARKSTGGKAPRKQLASKAARKSAPSTGGVKKPHRYKPGTVALREIRRYQKSTE
ScH3             1 MARTKQTARKSTGGKAPRKQLASKAARKSAPSTGGVKKPHRYKPGTVALREIRRFQKSTE
SpH3             1 MARTKQTARKSTGGKAPRKQLASKAARKAAPATGGVKKPHRYRPGTVALREIRRYQKSTE
HsH3.1           1 MARTKQTARKSTGGKAPRKQLATKAARKSAPATGGVKKPHRYRPGTVALREIRRYQKSTE
HsH3.3           1 MARTKQTARKSTGGKAPRKQLATKAARKSAPSTGGVKKPHRYRPGTVALREIRRYQKSTE

SNOG_03319      61 LLIRKLPFQRLVREIAQDFKSDLRFQSSAIGALQESVEAYLVSLFEDTNLCAIHAKRVTI
PTRG_11558.1    61 LLIRKLPFQRLVREIAQDFKSDLRFQSSAIGALQESVEAYLVSLFEDTNLCAIHAKRVTI
NcH3            61 LLIRKLPFQRLVREIAQDFKSDLRFQSSAIGLLQESVESYLVSLFEDTNLCAIHAKRVTI
ScH3            61 LLIRKLPFQRLVREIAQDFKTDLRFQSSAIGALQESVEAYLVSLFEDTNLAAIHAKRVTI
SpH3            61 LLIRKLPFQRLVREIAQDFKTDLRFQSSAIGALQEAVEAYLVSLFEDTNLCAIHGKRVTI
HsH3.1          61 LLIRKLPFQRLVREIAQDFKTDLRFQSSAVMALQEACEAYLVGLFEDTNLCAIHAKRVTI
HsH3.3          61 LLIRKLPFQRLVREIAQDFKTDLRFQSAAIGALQEASEAYLVGLFEDTNLCAIHAKRVTI

SNOG_03319     121 QSKDIQLARRLRGERG
PTRG_11558.1   121 QSKDIQLARRLRGERG
NcH3           121 QSKDIQLARRLRGERN
ScH3           121 QKKDIKLARRLRGERS
SpH3           121 QPKDMQLARRLRGERS
HsH3.1         121 MPKDIQLARRIRGERA
HsH3.3         121 MPKDIQLARRIRGERA
```

B

```
SNOG_15370    1 MAPKTPDRRNKARPSRGGRAS-------TGSRRESGKRVGQAGPAPDRAKKRYKPGTVAL
PtrCenH3      1 MAPKSPARRNKARPSRGGRVS-------VGSRKESGKRLGQPGPAPDRAKKRYKPGTVAL
Sc_Cse4       1 METEVPA------PVRTHSYA-------LDRYVRQKRREKQRKQSLKRVEKKYTPSELAL
Nc_CenH3      1 MPPKKGGVTKSKAVSKKAAAVPTPKATPPGRRKSRASSVQPGDPVPQGKKRRYRPGTLAL
Sp_Cnp1       1 -------------MAKKSLMA------------------EPGDPIPRPRKKRYRPGTTAL
MmCENpA       1 MGPRR---KPQTPRRR-PS-S-------------PAPGPSRQSSSVGSQTLRR-RQKFMWL
HsCENpA       1 MGPRRRSRKPEAPRRRSPSPT-------------PTPGPSRRGPSLGASSHQHSRRRQGWL

SNOG_15370   54 REIRRYQKSTDLLLLRTPFQRLVREIAQTVTT---ETGPTRWQSQAIQALQEATEAFLVN
PtrCenH3     54 REIKRYQKTTDLLLLKLPFQRLVREIAQSVTT---EDGPNRWQSQAIMALQEATEAFLVN
Sc_Cse4      48 YEIRKYQRSTDLLISKIPFARLVKEVTDEFTT---KDQDLRWQSMAIMALQEASEAYLVG
Nc_CenH3     61 KEIRNYQRTTDLLVAKLPFARLVREIAMQFRP---MDEEMRWQSQAILALQEAAEAFLVH
Sp_Cnp1      30 REIRKYQRSTDLLIQRLPFSRIVREISSEFVANFSTDVGLRWQSTALQCLQEAAEAFLVH
MmCENpA      43 KEIKTLQKSTDLLFRKKPFSMVVREICEKFSR----GVDFWWQAQALLALQEAAEAFLIH
HsCENpA      49 KEIRKLQKSTHLLIRKLPFSRLAREICVKFTR----GVDFNWQAQALLALQEAAEAFLVH

SNOG_15370  111 LFHDANLCAIHAKRVTIQQKDIQLARRLRAAWGAPV--
PtrCenH3    111 LFHDANLCAIHAKRVTIQQKDIQLARRLRAAWGAPV--
Sc_Cse4     105 LLEHTNLLALHAKRITIMKKDMQLARRIRGQFI-----
Nc_CenH3    118 LFEDTNLCAIHAKRVTIMQKDIQLARRIRGVWGGAGWV
Sp_Cnp1      90 LFEDTNLCAIHAKRVTIMQRDMQLARRIRGA-------
MmCENpA      99 LFEDAYLLSLHAGRVTLFPKDIQLTRRIRGFEGGLP--
HsCENpA     105 LFEDAYLLTLHAGRVTLFPKDVQLARRIRGLEEGLG--
```

Figure S4 Alignment of *bona fide* histone H3 proteins from *Ptr* with those of other fungi reveal the expected level of conservation. (A) Histone H3 is extremely well conserved among *Ptr*, *S. nodorum* (Sn), *N. crassa* (Nc), *Schizosaccharomyces pombe* (Sp), *Saccharomyces cervisiae* (Sc) and strongly resembles the replication-dependent H3.1 or replication-independent H3.3 from human (Hs). This is in stark contrast to alignments with the novel family of H3L proteins identified in *Ptr* (see Figure 3B). (B) Centromere-specific variants of H3, called CenH3 or CenpA, have variable N-terminal regions and more conserved histone-fold and C-terminal domains. While this variability suggested that H3Ls may be similar to CenH3 proteins, alignments of the histone-fold domain suggest that H3Ls are derived from canonical H3s (see Figure 3B). In both figures, completely conserved residues are shown in black, similar residues are in green and variable residues are in red.

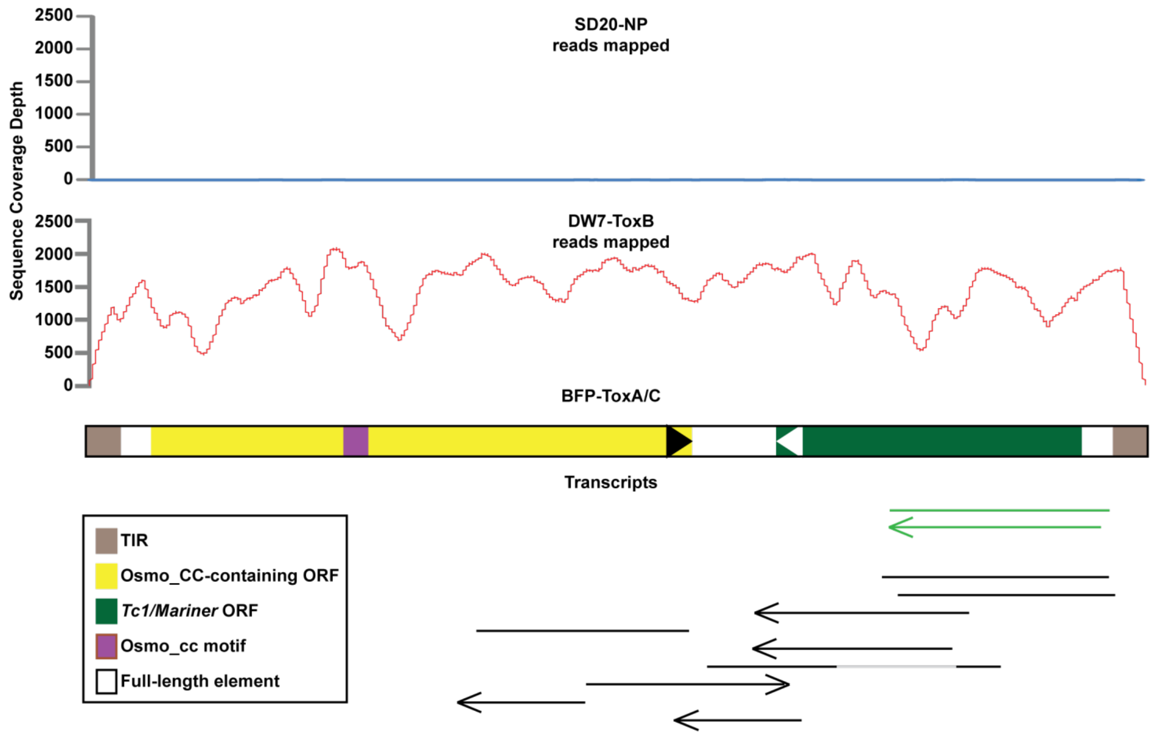

Figure S5 Transduplication of osmosensory transporter coiled-coil (Osmo-CC) domain in *Ptr*. The bar represents a full length element (3.4-kb) of this repeat family present in the reference genome of BFP-ToxAC. The graphs on the top are a result of mapping of all Illumina sequencing reads from each isolate to the reference and represents the sequence depth at that position. Arrows and lines below the element representation indicate alignments of transcripts present in various libraries (arrows - polyA tails, light gray - introns).

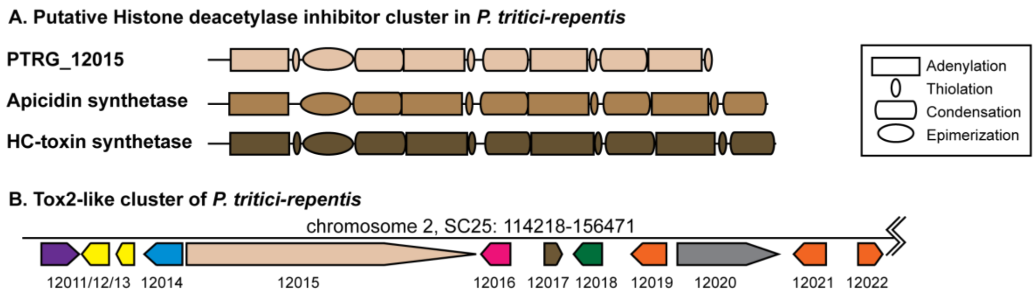

P. tritici-repentis		Best BlastP hit			
**Locus**	**Proposed function**	**Accession Number**	**Organism**	**Annotation**	**e value**
12011	FAD-linked oxidoreductase	ACZ66255.1	*F. incarnatum*	APS9	9e-160
		XP_001209092.1	*A. terreus*	conserved hypothetical	4e-104
12012	Short chain dehydrogenase	XP_965074.1	*N. crassa*	conserved hypothetical	8e-80
		XP_001239054.1	*C. immitis*	conserved hypothetical	1e-77
12013	Short chain dehydrogenase	XP_001820062.1	*A. oryzae*	hypothetical protein	3e-68
		XP_002374381.1	*A. flavus*	short chain type dehydrogenase, putative	9e-68
12014	MFS transporter	ACZ66257.1	*F. incarnaturm*	APS11	1e-123
		sp:Q00357	*C. carbonum*	HC-toxin efflux carrier	2e-102
12015	NRPS	ACZ66258.1	*F. incarnatum*	APS1	0.0
		sp:Q01886.2	*C. carbonum*	HC-toxin synthetase	0.0
12016	Basic leucine zipper transcription factor	ACZ66248.1	*F. incarnatum*	APS2	2e-26
		sp:O74205	*C. carbonum*	Transcription factor ToxE	2e-16
12017	Thioesterase	XP_001799397.1	*S. nodorum*	hypothetical protein SNOG_09095	2e-24
		XP_002378766.1	*A. flavus*	hypothetical protein	1e-23
12018	Aminotransferase	sp:Q9Y885.1	C. carbonum	Aminotransferase ToxF	9e-123
		ACZ66250.1	*F. incarnatum*	APS4	6e-105
12019	cytochrome P450	XP_001555869.1	*B. fuckeliana*	hypothetical protein	4e-105
		XP_001795303	*S. nodorum*	hypothetical protein	1e-96
12020	Fatty acid synthase	ACZ66251.1	*F. incarnatum*	APS5	0.0
		XP_002146311.1	*P. marneffei*	3-oxoacyl-[acyl-carrier-protein] synthase	0.0
12021	cytochrome P450	XP_001275091.1	*A. clavatus*	benzoate 4-monooxygenase, cytochrome P450	3e-134
		ACZ66253.1	*F. incarnatum*	APS7	8e-125
12022	cytochrome P450	ACZ66254.1	*F. incarnatum*	APS8	2e-109
		XP_002477830.1	*T. stipitatus*	cytochrome P450, putative	2e-76

Figure S6 Modular architecture and biosynthetic cluster of a putative histone deacetylase inhibitor biosynthetic cluster in *P. tritici-repentis.* (A) Modular architecture of PTRG_12015, APS1 (Apicidin synthetase) and HTS1 (HC-toxin synthetase). Domains (Adenylation, Thiolation, Condensation, and Epimerization) and spacing are to scale. (B) Putative biosynthetic cluster containing PTRG_12015. Putative open reading frames are designated as arrows. Proteins with similar function are coded with a similar color. Blastp search was performed against the NCBI nr database. Proposed function as determined by KOG or GO classification.

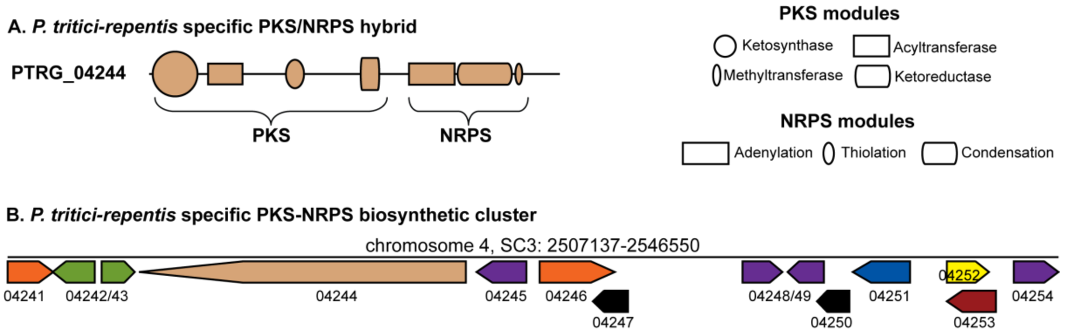

P. tritici-repentis		Best BlastP hit			
**Locus**	**Proposed function**	**Accession Number**	**Organism**	**Annotation**	**e value**
04241	cytochrome p450	gb|EEQ33486.1	*M. canis*	n-alkane inducible cytrochrome P450	9e-168
04242	Flavoprotein mono-oxygenase	XP_001801158.1	*S. nodorum*	conserved hypothetical	3e-109
04243	2OG-Fe(II) oxygenase	XP_001905187.1	*P. anserina*	unnamed protein product	3e-68
04244	PKS:NPS hybrid	XP_001905191.1	*P. anserina*	unnamed protein product	0.0
04245	FAD-linked oxidase	XP_391370.1	*F. graminearum*	hypothetical protein	1e-162
04246	cytochrome p450	XP_002487173.1	*T. stipitatus*	cytochrome P450 monooxigenase	2e-120
04247	predicted protein				
04248	alchohol oxidase	gb|EEU33852	*N. haematococca*	hypothetical protein	2e-117
04249	oxidoreductase	XP_002479371.1	*T. stipitatus*	oxidoreductase, putative	1e-162
04250	hypothetical protein	gb|EEU35594.1	*N. haematococca*	hypothetical protein	4e-08
04251	MFS efflux pump	XP_002341986.1	*T. stipitatus*	efflux pump antibiotic resistance protein, putative	2e-136
04252	putative short chain dehydrogenase	XP_001229641.1	*C. globosum*	hypothetical protein	2e-66
04253	O-methyltransferase	XP_001550242.1	*B. fuckeliana*	hypothetical protein	2e-66
04254	FAD-linked oxidase	XP_002483865.1	*T. stipitatus*	FAD-dependent isoamyl alcohol oxidase, putative	6e-122

Figure S7 Modular architecture and biosynthetic cluster of a *P. tritici-repentis*-specific PKS/NRPS hybrid. (A) Modular architecture of PTRG_04244. Domains and spacing are to scale. (B) Putative biosynthetic cluster containing PTRG_04244. Putative proteins are designated as arrows. Proteins with similar function are coded with a similar color. Blastp search against GenBank nr database. Proposed function as determined by KOG or GO classification.

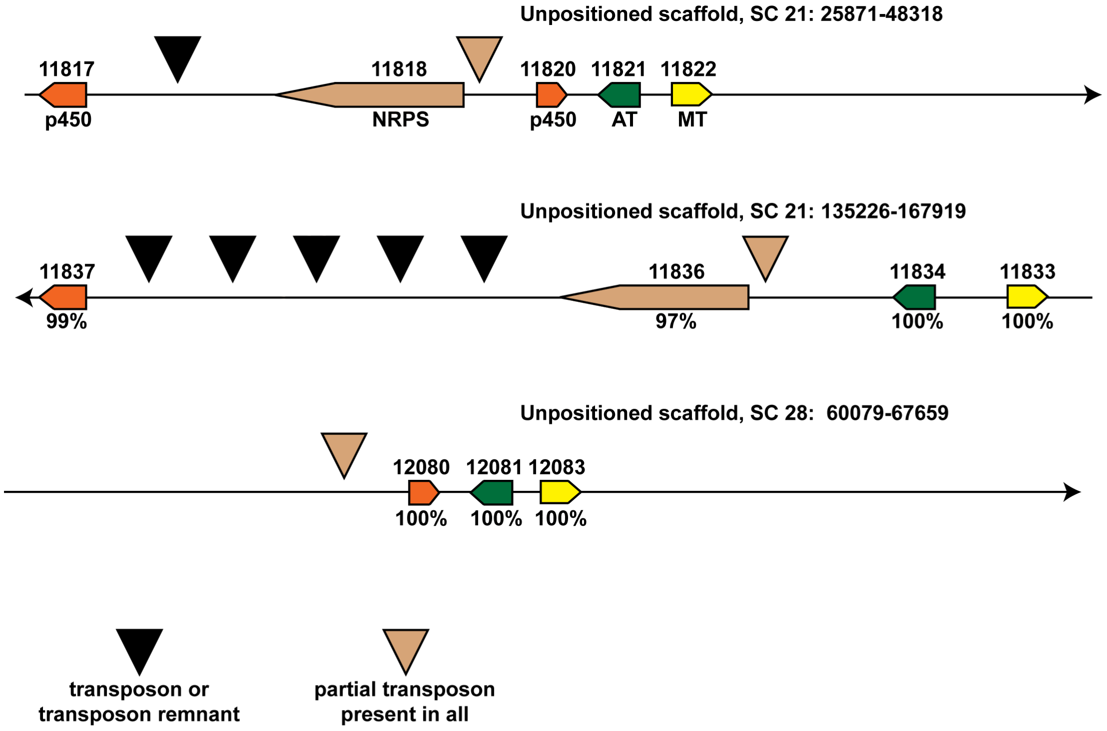

Figure S8 Duplicated genomic regions associated with the NRPSs PTRG_11818 & 11836. Putative proteins are designated as arrows. Proteins with similar function are coded with similar color. AT - -acyltransferase; MT - methyltransferase. Protein coding regions and spacing between are to scale. Numbers above boxes indicate locus postion in the reference. Numbers beneath boxes represent the % identity of the proteins relative to PTRG_11817 through 11822. Triangles represent transposons or transposon remnants.

Table S1 ***P. tritici-repentis*** **isolates used in this study**

Isolate name	Race	Geographic location	Path/non-path	Analyses
BFP-ToxA/C	1	S. Dakota, USA	Path	Reference Genome/EST/PCR
ASC1	1	Manitoba, Canada	Path	PCR
86-124	2	Manitoba, Canada	Path	PCR
D308	3	Manitoba, Canada	Path	PCR
DW2	5	N. Dakota, USA	Path	PCR
DW7	5	N. Dakota, USA	Path	Resequencing
SO3	?	Oregon, USA	Path	EST/PCR
SD20-NP	4	S. Dakota, USA	Non-path	Resequencing/EST/PCR
90-2	4	Manitoba, Canada	Non-path	PCR
98-31-2	4	N. Dakota, USA	Non-path	PCR

Table S2 ***P. tritici-repentis* reference genome libraries sequenced**

Library	Reads	Physical Coverage (Fold)	Sequence Coverage * (Fold)
4kb Plasmid	266,377	14	4.73
10kb Plasmid	56,616	9	1.11
40kb Fosmid	64,483	35	1.09
**Total**	**387,476**	**58**	**6.93**

* Q20 base coverage

Table S3 ***P. tritici-repentis*** **assembly anchored to the optical maps**

Optical Linkage group	Estimated Size (Mb)	Mapped Scaffolds	Scaffold Size (Mb)
chr1	10.166	10, 33, 18,1,17	9.504
chr2	5.206	16,2,25,23	4.322
chr3	3.708	12,14,13	3.361
chr4	3.301	3	3.079
chr5	3.151	5	2.677
chr6	2.951	4	2.787
chr7	2.753	30,15,11	2.612
chr8	2.391	8,24,26	2.115
chr9	2.287	9,22,27	2.026
chr10	2.204	6	1.985
chr11	2.113	7	1.888
**Total**	**40.231**		**36.355**

Table S4 Illumina sequenced *P. tritici-repentis* isolate read alignment under different stringencies

	All repeats/Best hit* (Low Stringency)		No repeats/Best hit* (Medium Stringency)		No repeats/Unique hit** (High Stringency)	
	DW7	SD20	DW7	SD20	DW7	SD20
PE alignment (M)	75.59	56.87	22.18	44.10	21.65	36.40
SE alignment (M)	5.80	9.11	0.52	3.43	0.41	2.20
Unmapped (M)	2.92	17.26	6.72	19.56	7.36	28.49
Paired read count (M)	14.71	33.54				
**Total read count (M)**	**29.42**	**67.09**				

*Best hit = up to 4 mismatches; **Unique = no mismatches; Mapping performed with SOAP2

Table S5 Repeat families in *P. tritici-repentis*

Available for download at http://www.g3journal.org/lookup/suppl/doi:10.1534/g3.112.004044/-/DC1.

Table S6 EST libraries produced to facilitate gene calling

Libraries	Pathogenicity	Conditions	Reads	Spans	Average Sequence Identity %
Pt-1C(BFP)	Yes-Race 1	In planta	10,083	3,088	
Pt-1C(BFP)	Yes-Race 1	Mixed culture	10,444	12,198	
SO3-P	Yes-Race 9	Mixed culture	10,185	12,115	98.08
SD20-NP	No-Race 4	Mixed culture	10,328	8,195	98.2

Table S7 Serine codon usage in *P. tritici-repentis*

Ser codon	% usage	# of codons
AGC	19.4	118705
AGT	12.4	76150
TCA	18.4	112959
TCC	16.4	100858
TCG	16.8	103107
TCT	16.6	101583
**Total**	**100**	**613362**

Table S8 Top five repeat familes shared between the *P. tritici-repentis* reference genome and resequenced pathogenic and non-pathogenic isolates

	Reference family	Repeat class	Annotation*
Pathogenic - DW7-ToxB			
	8	DNA transposon	*hAT* superfamily; similar to Tfo1 from *F. oxysporum*
	63	DNA transposon	*hAT* superfamily; similar to Restless
	105	DNA transposon	*Tc1/Mariner* superfamily
	49	DNA transposon	*Tc1/Mariner* superfamily; similar to Molly from *S. nodorum*
	619	DNA transposon	*hAT* superfamily; similar to Restless
Non-pathogenic-SD20-NP			
	26	Non-LTR retrotransposon	similar to Tad1 of *Blumeria*
	27	Non-LTR retrotransposon	similar to Tad1 of *Blumeria*
	238	Non-LTR retrotransposon	similar to Tad1 of *Blumeria*
	116	unknown	
	293	unknown	

*Repeat annotation performed in Censor

Table S9 Count of identical 16-mers in 1X sampling of resequencing reads of DW7-ToxB and SD20-NP

	DW7-ToxB		SD20-NP	
Times present	Count	% of Total	Count	% of Total
1 to 10	21571304	88.03	22331413	94.66
11 to 20	1585463	6.47	534634	2.27
21 to 30	523010	2.13	309650	1.31
31 to 40	251216	1.03	250797	1.06
41 to 50	113859	0.46	102102	0.43
51 to 60	24937	0.10	41720	0.18
61 to 70	7766	0.03	9912	0.04
71 to 80	3857	0.02	3513	0.01
81 to 90	6792	0.03	1368	0.01
91 to 100	4498	0.02	382	0.00
101 to 110	2746	0.01	631	0.00
111 to 120	1566	0.01	737	0.00
121 to 130	2732	0.01	280	0.00
131 to 140	6719	0.03	0	0.00
141 to 150	11469	0.05	378	0.00
151 to 160	11769	0.05	2237	0.01
161 to 170	4064	0.02	900	0.00
171 to 180	4337	0.02	0	0.00
181 to 190	5051	0.02	0	0.00
191 to 200	4506	0.02	0	0.00
201 to 210	4314	0.02	0	0.00
211 to 220	3851	0.02	0	0.00
221 to 230	8033	0.03	0	0.00
231 to 240	12958	0.05	0	0.00
241 to 250	16405	0.07	0	0.00
251 to 260	11951	0.05	0	0.00
261 to 270	11559	0.05	0	0.00
271 to 280	9348	0.04	0	0.00
281 to 290	14204	0.06	0	0.00
291 to 300	18206	0.07	0	0.00
301 to 310	21485	0.09	0	0.00
311 to 320	36139	0.15	0	0.00
321 to 330	35316	0.14	0	0.00
331 to 340	38744	0.16	0	0.00
341 to 350	32057	0.13	0	0.00
351 to 360	23219	0.09	0	0.00
361 to 370	10710	0.04	0	0.00
371 to 380	10067	0.04	0	0.00

381 to 390	11319	0.05	0	0.00
391 to 400	10130	0.04	0	0.00
401 to 410	9831	0.04	0	0.00
411 to 417	6554	0.03	0	0.00
**Total**	**24504061**		**23590654**	

Table S10 Histone genes present in *P. tritici-repentis*

Available for download at http://www.g3journal.org/lookup/suppl/doi:10.1534/g3.112.004044/-/DC1.

Table S11 Histone genes present in *Puccinia graminis*

Available for download at http://www.g3journal.org/lookup/suppl/doi:10.1534/g3.112.004044/-/DC1.

Table S12 Pfam domains in repeats*

PFAM description	# in repeats
AMP-binding enzyme	6
AT hook motif	26
Bacterial regulatory helix-turn-helix proteins, AraC family	2
Bacterial regulatory proteins, lacI family	2
Bacterial SH3 domain	2
Centromere binding protein B, DNA binding	3
'chromo' (CHRromatin Organisation MOdifier) domain	32
Condensation domain	6
Core histone H2A/H2B/H3/H4	33
DDE superfamily endonuclease	111
hAT family dimerisation domain	178
helix-turn-helix, Psq domain	72
Integrase core domain	157
LTXXQ motif	14
MORN repeat variant	5
Motilin/ghrelin	7
Opioid growth factor receptor repeat	2
Osmosensory transporter coiled coil	23
Phosphopantetheine attachment site	8
PPAK motif	1
Protein of unknown function (DUF1299)	1
Protein of unknown function (DUF1409)	4
Retrotransposon gag protein	4
Reverse transcriptase (RNA-dependent DNA polymerase)	139
RNase H	33
SAMP Motif	1
Transposase	1
Viral A-type inclusion protein repeat	16
WCCH motif	19
WD domain, G-beta repeat	1
Zinc knuckle	28

*Pfam domains predicted by Broad Institute and present in custom repeat library

Table S13 *De novo* **assembly of resequenced P. tritici-repentis isolates***

	DW7-ToxB	SD20-NP
k-mer	41	41
# contigs	21300	40383
largest contig (nt)	225936	320323
L50 (nt)	39556	69524
% contigs > 10 kb	85	88
% nt in contigs > 500 nt	98	98

* Assembly performed in Velvet

Table S14 Predicted secretome of *P. tritici-repentis*

Available for download at http://www.g3journal.org/lookup/suppl/doi:10.1534/g3.112.004044/-/DC1.

Table S15 Putative NRPS-containing clusters in the *P. tritici-repentis* reference genome

P. tritici-repentis locus	Size (aa)	Putative function
PTRG_04244		
PTRG_04241	461	p450
PTRG_04242	410	flavoprotein monooxygenase
PTRG_04243	322	2OG-Fe(II) oxygenase
PTRG_04244	3850	pks:nrps hybrid
PTRG_04245	558	FAD-linked oxidase
PTRG_04246	491	p450 monooxigenase
PTRG_04247	384	predicted protein
PTRG_04248	452	alcohol oxidase
PTRG_04249	407	oxidoreductase
PTRG_04250	169	hypothetical protein
PTRG_04251	557	MFS efflux pump transporter
PTRG_04252	297	putative short chain dehydrogenase
PTRG_04253	401	O-methyltransferase
PTRG_04254	540	FAD-linked oxidase
PTRG_00447		
PTRG_00447	1282	nrps10
PTRG_00448	182	
PTRG_00449	199	
PTRG_00450	317	cyclin d-interacting protein
PTRG_00451	295	TBP-binding protein, activator of basal transricption
PTRG_00452	363	uncharacterized, conserved
PTRG_00453	347	
PTRG_00454	421	p450
PTRG_00455	317	unbiquitin ligase
PTRG_00456	296	zinc finger protein
PTRG_00457	554	AMP domain
PTRG_01683		
PTRG_01677	548	oxidoreductase
PTRG_01678	603	Long chain fatty acid acyl-CaA ligase
PTRG_01679	438	
PTRG_01680	423	Flavin-containing monooxygenase
PTRG_01681	1299	ABC transporter
PTRG_01682	219	N-acetyltransferase
PTRG_01683	1841	nrps6
PTRG_01684	475	
PTRG_01685	360	tyrosinase

PTRG_01800		
PTRG_01796	1270	splicing coactivator
PTRG_01797	539	MFS
PTRG_01798	89	
PTRG_01799	1489	ABC transporter
PTRG_01800	6909	nrps4
PTRG_01801	79	
PTRG_01802	369	Malic acid transporter
PTRG_01803	187	secreted protein
PTRG_01804	172	
PTRG_01805	507	p450
PTRG_01806	689	F-box domain
PTRG_01807	294	cyclin-like F-box domain
PTRG_08276		
PTRG_08272	858	translation initiation factor
PTRG_08273	906	YAKI-serine threonine kinase
PTRG_08274	529	Flavin-containing monooxygenase
PTRG_08275	1310	ABC transporter
PTRG_08276	5326	nrps2
PTRG_08280	402	Lipid phosphatase, PAP2 family
PTRG_08281	161	DNA-binding protein
PTRG_08282	476	mRNA splicing factor
PTRG_08283	276	transcription factor
PTRG_08284	1049	von Willebrands; may be two proteins or misannotated
PTRG_08285	743	AAA+-type ATPase
PTRG_08286	706	
PTRG_08287	855	RNA-binding protein
PTRG_08288	445	Hormone sensitive-lipase
PTRG_08289	165	
PTRG_08290	144	DNA methyltransferase
PTRG_08291	349	
PTRG_09101		
PTRG_09095	1432	ABC transporter
PTRG_09096	328	RTA1-like protein
PTRG_09097	391	Aminotransferase, class IV
PTRG_09098	2054	Fatty acid synthase, beta
PTRG_09099	1590	Beta-ketoacyl synthase
PTRG_09100	537	P450
PTRG_09101	6024	nrps
PTRG_09102	446	kynurenine 3-monooxygenase

PTRG_09103	552	MFS
PTRG_12015		
PTRG_12011	597	FAD-linked oxidoreductase
PTRG_12012	305	Short chain dehydrogenase
PTRG_12013	266	Short chain dehydrogenase
PTRG_12014	564	MFS transporter
PTRG_12015	4634	nrps HDAC
PTRG_12016	470	Basic leucine zipper transcription factor
PTRG_12017	242	Thioesterase
PTRG_12018	339	Aminotransferase
PTRG_12019	532	Cytochrome P450
PTRG_12020	1618	Fatty acid synthase
PTRG_12021	437	Cytochrome P450
PTRG_12022	338	Cytochrome P450

Table S16 Putative polyketide synthetase gene of *P. tritici-repentis*

Available for download at http://www.g3journal.org/lookup/suppl/doi:10.1534/g3.112.004044/-/DC1.

Table S17 ***Pyrenophora tritici-repentis*-specific secreted proteins as predicted in Blast2GO**

Locus	Size (aa)	% Cys	HMM	E-value	Present DW7	Present SD20	EST			
							Race1 culture	Race1 Planta	Race4 culture	Race9 culture
					Race 1 specific					
PTRG_05296	118	0.85			no	no				
PTRG_04919	123	0.82			no	no				
PTRG_10524	59	6.9			no	no				
					Pathogen Specific					
PTRG_06853	122	1.65			yes	no				
PTRG_11888	86	14.12			yes	no	7	8		4
PTRG_12138	73	9.72			yes	no	14	2		
					Discontinuously distributed					
PTRG_09431	73	4.17			no	yes				
					Present in all sequenced isolates					
PTRG_00723	184	0.55			yes	yes				
PTRG_05020	85	0	GRP	0.0022	yes	yes				2
PTRG_10257	61	0			yes	yes	2		14	7
PTRG_00384	63	1.61			yes	yes				
PTRG_00529	52	0			yes	yes				
PTRG_00558	80	10.13			yes	yes		2		
PTRG_00758	75	4.05			yes	yes				
PTRG_00935	54	9.43			yes	yes				
PTRG_01132	87	2.33			yes	yes				
PTRG_01371	54	11.32			yes	yes		2		3
PTRG_01382	201	0			yes	yes				
PTRG_01617	59	0			yes	yes				
PTRG_01675	55	3.7			yes	yes				
PTRG_01823	133	6.06			yes	yes				
PTRG_01890	50	4.08			yes	yes				
PTRG_01923	78	7.79			yes	yes				
PTRG_02034	128	3.94			yes	yes				
PTRG_02202	90	2.25			yes	yes				
PTRG_02218	92	3.3			yes	yes				
PTRG_02241	99	6.12			yes	yes				
PTRG_02456	90	1.12			yes	yes				
PTRG_02606	91	5.56	PRA1	0.098	yes	yes				
			Innexin	0.036						
PTRG_02982	76	10.67			yes	yes				
PTRG_03151	69	1.47			yes	yes				
PTRG_03183	50	4.08			yes	yes	2	1		

PTRG_03424	129	6.25			yes	yes				
PTRG_03447	65	0			yes	yes				
PTRG_03460	79	2.56			yes	yes				
PTRG_03545	92	6.59			yes	yes				
PTRG_03740	137	0.74			yes	yes				
PTRG_03833	155	6.49			yes	yes				
PTRG_03972	117	3.45			yes	yes				
PTRG_04061	95	7.45			yes	yes				
PTRG_04438	132	3.05			yes	yes				
PTRG_04467	75	1.35			yes	yes				
PTRG_04476	130	3.88			yes	yes				
PTRG_04489	137	3.68			yes	yes				
PTRG_04511	57	5.36			yes	yes				
PTRG_04567	113	6.25	Peptidase_M19	0.076	yes	yes			2	2
			Meleagrin	0.089						
PTRG_04595	127	3.97			yes	yes				
PTRG_04645	109	3.7			yes	yes				
PTRG_04835	99	8.16			yes	yes			2	
PTRG_04870	73	9.72			yes	yes				
PTRG_05019	142	0			yes	yes				
PTRG_05021	143	1.41			yes	yes				
PTRG_05037	130	1.55			yes	yes				
PTRG_05069	95	6.38			yes	yes				
PTRG_05127	95	6.38			yes	yes				
PTRG_05187	141	0			yes	yes	8			2
PTRG_05203	130	0.78			yes	yes				2
PTRG_05392	96	3.16			yes	yes				
PTRG_05427	81	1.25	DEC-1_N	0.0014	yes	yes				
			PLRV_ORF5	0.048						
PTRG_05658	110	7.34			yes	yes				
PTRG_05788	395	0.51			yes	yes				2
PTRG_05858	77	0			yes	yes				
PTRG_05999	77	2.63			yes	yes				
PTRG_06015	56	1.82			yes	yes				
PTRG_06042	119	3.39			yes	yes				
PTRG_06295	57	0			yes	yes	2			
PTRG_06429	140	6.47			yes	yes		2		
PTRG_06505	295	0			yes	yes				
PTRG_06517	104	7.77			yes	yes				
PTRG_06556	94	3.23			yes	yes				
PTRG_06557	52	1.96			yes	yes				2
PTRG_06608	53	1.92			yes	yes				
PTRG_07001	69	8.82			yes	yes				

PTRG_07260	292	3.78			yes	yes				
PTRG_07271	88	5.75			yes	yes				
PTRG_07510	74	1.37			yes	yes				
PTRG_07515	128	7.09			yes	yes				
PTRG_07580	168	0	PIR	0.0000046	yes	yes	10	2	6	4
PTRG_07935	80	1.27	Peptidase_M66	0.0094	yes	yes				
PTRG_07936	164	3.68			yes	yes				
PTRG_08136	76	2.67	Mfp-3	0.032	yes	yes				
PTRG_08379	69	2.94			yes	yes	8		2	
PTRG_08398	107	1.89			yes	yes				
PTRG_08448	130	3.1			yes	yes				
PTRG_08572	87	16.28			yes	yes	2			
PTRG_08636	60	0			yes	yes				
PTRG_09109	92	6.59			yes	yes				
PTRG_09139	105	3.85			yes	yes	6			
PTRG_09257	293	1.71			yes	yes				
PTRG_09351	77	10.53			yes	yes				2
PTRG_09380	96	7.37			yes	yes	2		1	
PTRG_09458	49	4.17			yes	yes	2			
PTRG_09580	459	1.97			yes	yes				
PTRG_09958	97	6.25			yes	yes				
PTRG_10036	54	1.89			yes	yes				
PTRG_10069	70	4.35			yes	yes				
PTRG_10109	153	0.66			yes	yes	8		2	2
PTRG_10127	119	2.54			yes	yes	2			4
PTRG_10138	130	0			yes	yes				
PTRG_10852	61	5			yes	yes				
PTRG_10951	142	1.42			yes	yes				
PTRG_10990	123	3.28			yes	yes				
PTRG_11024	51	4			yes	yes				
PTRG_11036	135	4.48			yes	yes				
PTRG_11089	64	9.52			yes	yes	6	3		2
PTRG_11261	70	5.8			yes	yes				
PTRG_11351	94	0			yes	yes				
PTRG_11361	122	4.96			yes	yes				
PTRG_11416	60	1.69			yes	yes	2			
PTRG_11417	65	4.69	PsaX	0.05	yes	yes				

Table S18 Total numbers of predicted CAZymes in *PTR* and selected ascomycetes

Species	GH	GT	PL	CE	CBM	EXPN
Pyrenophora tritici-repentis	245	91	10	39	45	2
Mycosphaerella graminicola	191	99	3	18	21	3
Trichoderma reesei	192	93	6	17	48	4
Fusarium graminearum	247	102	21	44	67	4
Neurospora crassa	173	76	4	22	42	1
Magnaporthe grisea	232	92	5	47	65	1
Aspergillus nidulans	251	91	21	31	41	1
Stagonospora nodorum	273	89	10	50	61	4

GH = glycoside hydrolases; GT = glycosyl transferases; PL = polysaccharide lyases; CE =carbohydrate esterases; CBM = carbohydrate binding modules; EXPN = distantly related to plant expansins. Predicted CAZymes were identified using the carbohydrate-active enzymes database tools

Table S19 Comparison of pectinolytic enzymes in *P. tritici-repentis* vs. other grass and non-grass plant pathogens

	PL family							GH family					
Species	1	3	4	9	11	20	**Total**	28	78	88	95	105	**Total**
Magnaporthe grisea 70-15	2	1	1	0	0	1	**5**	3	1	1	1	3	**9**
Pyrenophora tritici-repentis Pt-1C-BFP	3	3	4	0	0	0	**10**	6	3	1	2	3	**15**
Phaeosphaeria nodorum SN15*	4	2	4	0	0	0	**10**	4	4	1	2	3	**14**
Gibberella zeae PH-1 (*F. graminearum*)	9	7	3	1	0	1	**21**	6	7	1	2	3	**19**
Gibberella moniliformis 7600 (*F. verticillioides*)	11	7	3	2	0	0	**23**	9	9	2	1	3	**24**
Fusarium oxysporum f. sp. lycopersici 4286	11	7	3	2	1	0	**24**	15	14	3	2	5	**39**
Verticillium albo-atrum VaMs.102	16	11	4	2	1	0	**34**	12	9	4	2	3	**30**
Verticillium dahliae VdLs.17	17	11	4	2	1	0	**35**	12	9	4	2	4	**31**

* Data from Amselem et al. 2011

Table S20 GO enrichment analysis of ESTs present in the in planta library*

GO Term	Name	FDR	FWER	single test p-Value
GO:0003735	structural constituent of ribosome	2E-09	7.1E-09	7.9E-14
GO:0046933	hydrogen ion transporting ATP synthase activity, rotational mechanism	0.0003	0.002	0.000017
GO:0008553	hydrogen-exporting ATPase activity, phosphorylative mechanism	0.0048	0.047	0.00033
GO:0015935	small ribosomal subunit	0.0052	0.051	0.00037
GO:0045263	proton-transporting ATP synthase complex, coupling factor F(o)	0.0088	0.092	0.00058
GO:0043581	mycelium development	0.017	0.18	0.0013
GO:0031176	endo-1,4-beta-xylanase activity	0.02	0.29	0.0021
GO:0030245	cellulose catabolic process	0.02	0.29	0.0021
GO:0008810	cellulase activity	0.043	0.54	0.0035
GO:0004784	superoxide dismutase activity	0.043	0.54	0.0035
GO:0006414	translational elongation	0.043	0.54	0.0035
GO:0006801	superoxide metabolic process	0.053	0.65	0.0054
GO:0009279	cell outer membrane	0.075	0.8	0.0069
GO:0003871	5-methyltetrahydropteroyltriglutamate-homocysteine S-methyltransferase activity	0.075	0.8	0.0069
GO:0019843	rRNA binding	0.079	0.82	0.0078
GO:0005576	extracellular region	0.083	0.85	0.01
GO:0006099	tricarboxylic acid cycle	0.088	0.89	0.012
GO:0004775	succinate-CoA ligase (ADP-forming) activity	0.11	0.95	0.013
GO:0043022	ribosome binding	0.11	0.95	0.013

* Fisher's exact test performed in Blast2GO; Most specific terms

Table S21 Comparison of CBM-containing protein families in *P. tritici-repentis* and other grass and non-grass plant pathogens

	CBM family																
Species	1	6	12	13	18	20	21	24	32	35	38	42	43	48	50	52	**Total**
Magnaporthe grisea 70-15	22	2	0	0	33	3	1	0	0	3	0	1	1	1	21	1	**89**
Pyrenophora tritici-repentis Pt-1C-BFP	11	1	0	0	19	2	1	0	1	3	0	1	2	3	0	0	**44**
Phaeosphaeria nodorum SN15 *	13	1	0	0	43	3	1	0	1	3	0	1	2	2	0	0	**70**
Gibberella zeae PH-1 (*F. graminearum*)	12	1	0	2	35	2	2	1	4	2	0	1	1	1	33	0	**97**
Gibberella moniliformis 7600 (*F. verticillioides*)	14	1	0	1	26	2	1	1	3	4	1	1	1	3	52	0	**111**
Fusarium oxysporum f. sp. lycopersici 4286	13	1	4	1	36	2	10	2	3	4	3	1	0	1	46	0	**127**
Verticillium albo-atrum VaMs.102	26	0	0	0	29	4	1	3	0	3	1	2	1	1	33	0	**104**
Verticillium dahliae VdLs.17	30	0	0	0	29	4	0	3	0	3	1	2	1	1	32	0	**106**

* Data from Anselem et al. 2011
