# Supplementary material for: Comparative Genomics of a Plant-Pathogenic Fungus, Pyrenophora tritici-repentis, Reveals Transduplication and the Impact of Repeat Elements on Pathogenicity and Population Divergence
Source: G3 (Bethesda). 2013 Jan 1;3(1):41–63. doi: 10.1534/g3.112.004044 (PMC3538342; doi:10.1534/g3.112.004044)
Supplement: Supporting Information [file supp_3.1.41_FigureS1.pdf]

A

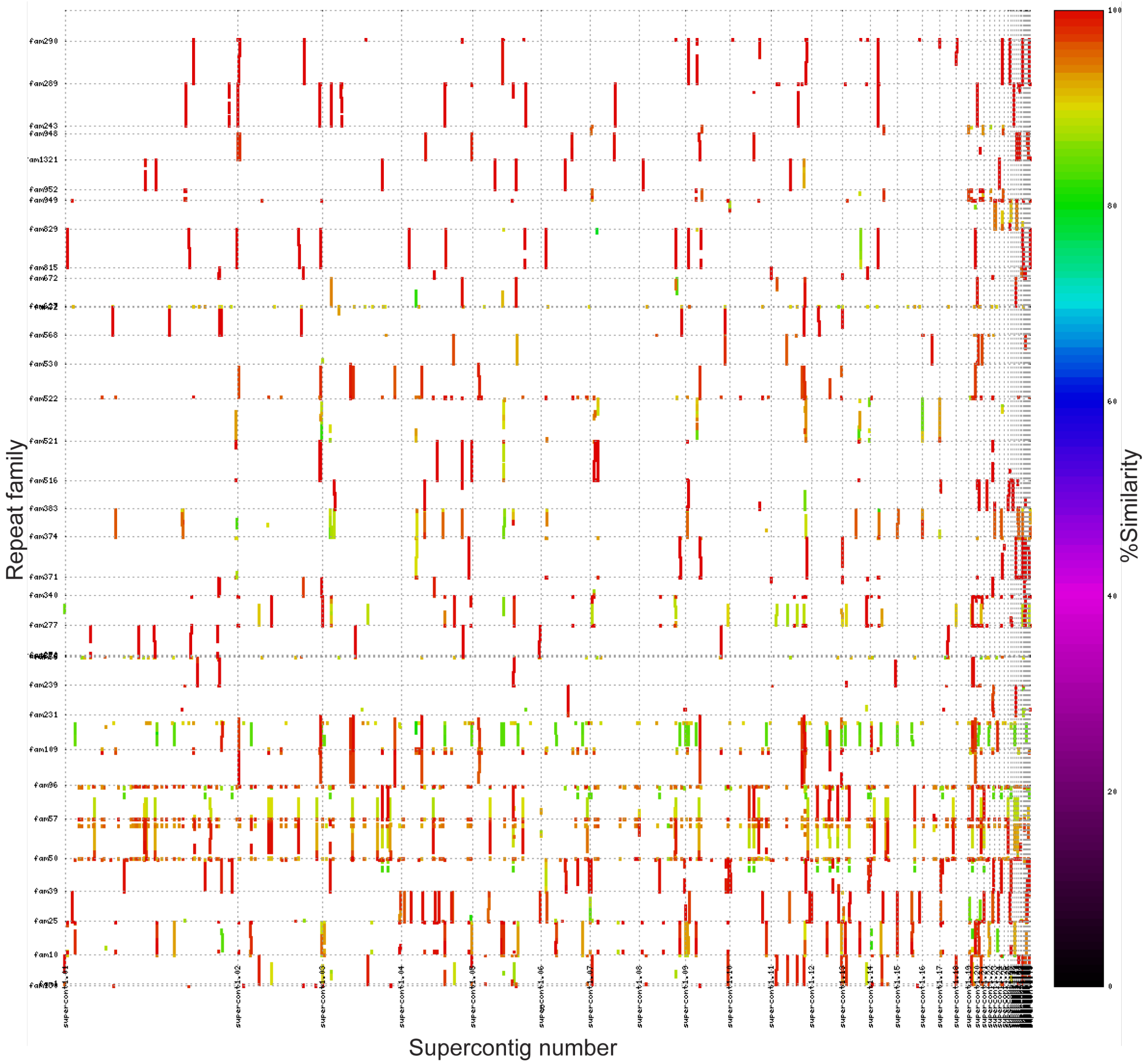
Repeat family
Supercontig number
%Similarity
fam290
fam289
fam243
fam948
fam1321
fam952
fam949
fam829
fam815
fam672
fam568
fam530
fam522
fam521
fam516
fam383
fam374
fam371
fam340
fam277
fam239
fam231
fam109
fam96
fam57
fam50
fam39
fam25
fam10
100
80
60
40
20
0

B

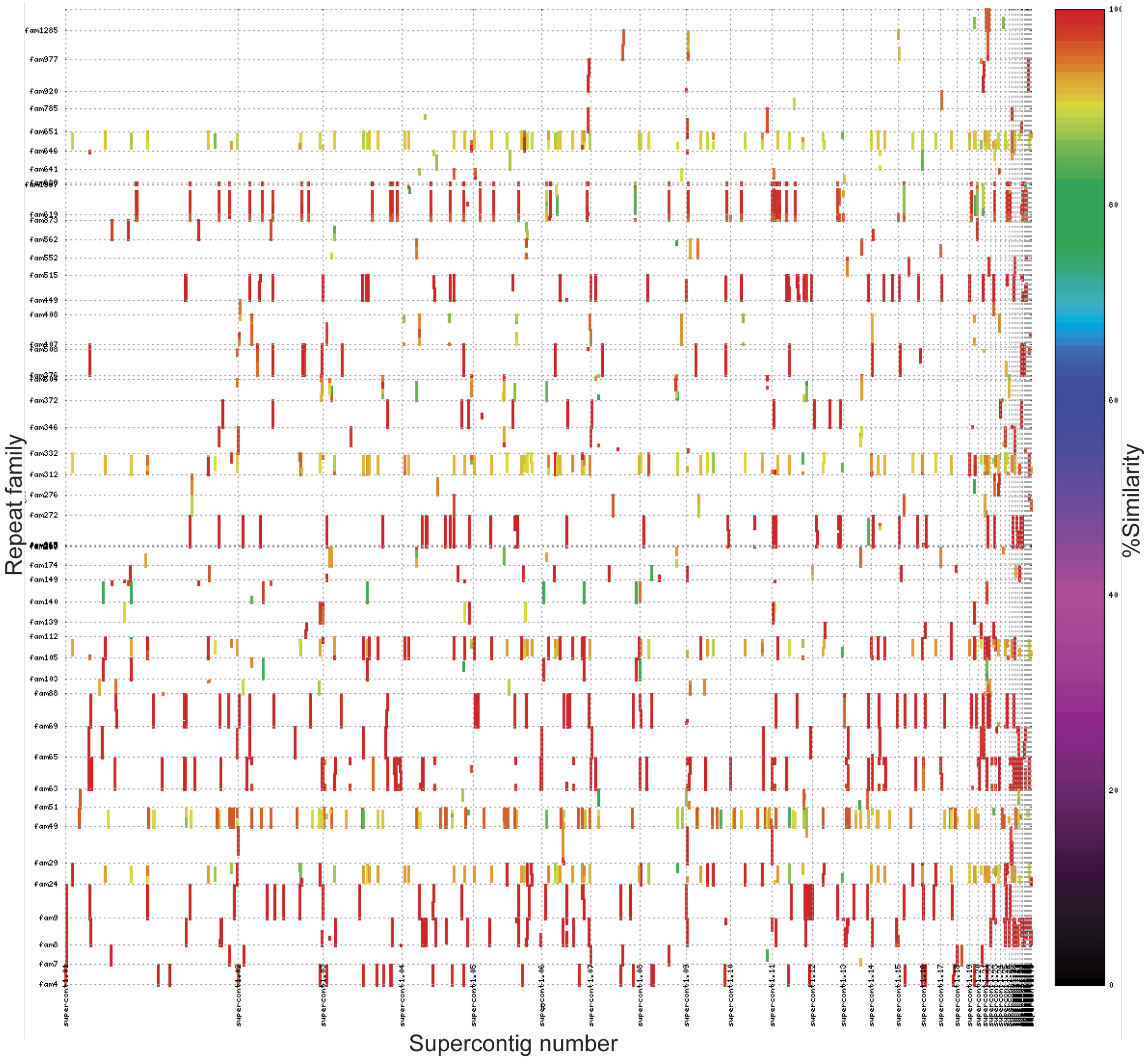
Repeat family
Supercontig number
%Similarity
100
80
60
40
20
0

C

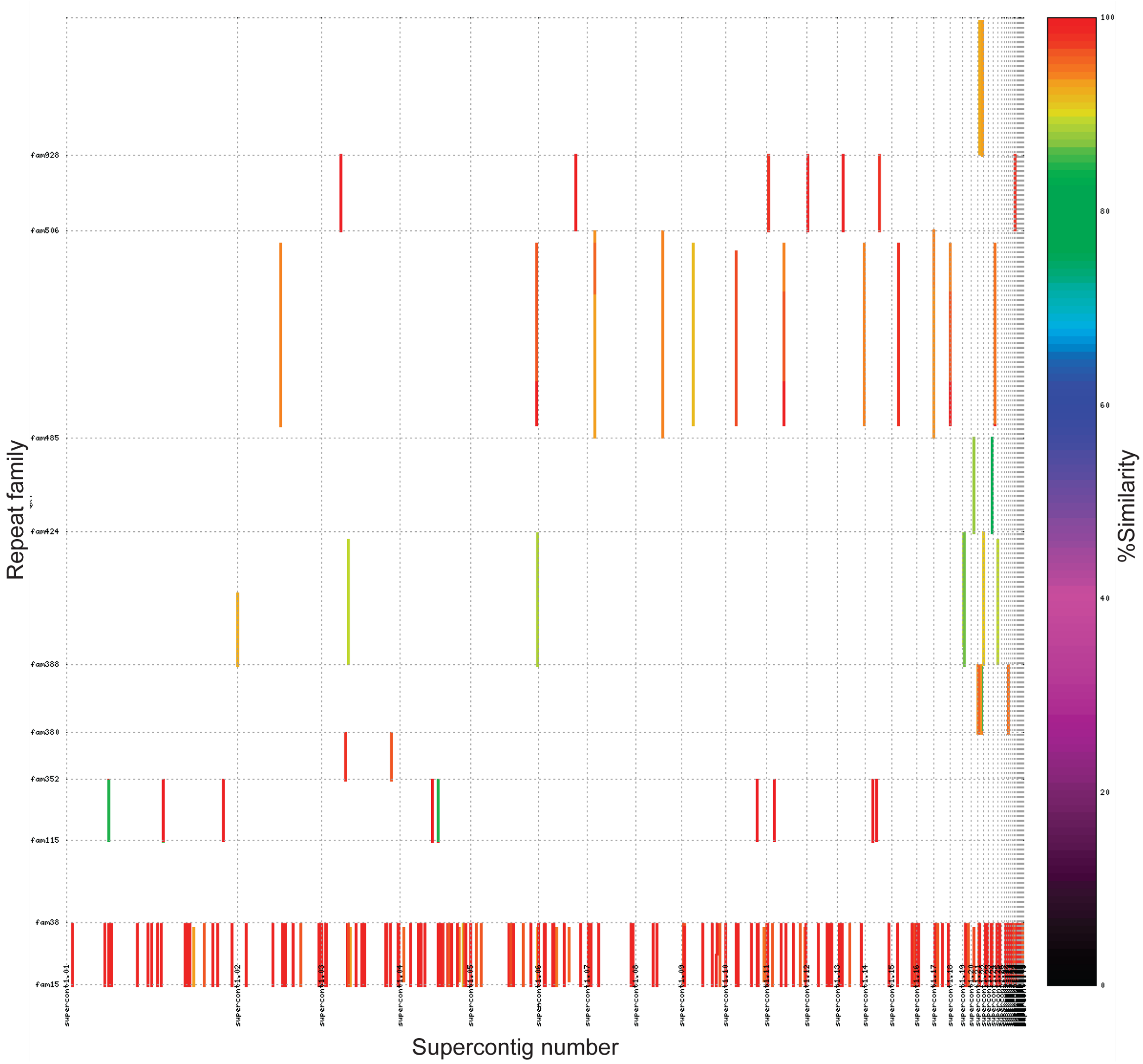

Figure S1 Similarity and distribution of (A) LTR retrotransposons, (B**)** DNA transposons and (C) MITES across the Supercontig assemblies of BFP-ToxAC. The repeat family name is indicated on the left. The color scale on the right indicates % similarity from 0 (black) to 100 (red).
