# Supplementary material for: Comparative Genomics of a Plant-Pathogenic Fungus, Pyrenophora tritici-repentis, Reveals Transduplication and the Impact of Repeat Elements on Pathogenicity and Population Divergence
Source: G3 (Bethesda). 2013 Jan 1;3(1):41–63. doi: 10.1534/g3.112.004044 (PMC3538342; doi:10.1534/g3.112.004044)
Supplement: Supporting Information [file supp_3.1.41_FigureS2.pdf]

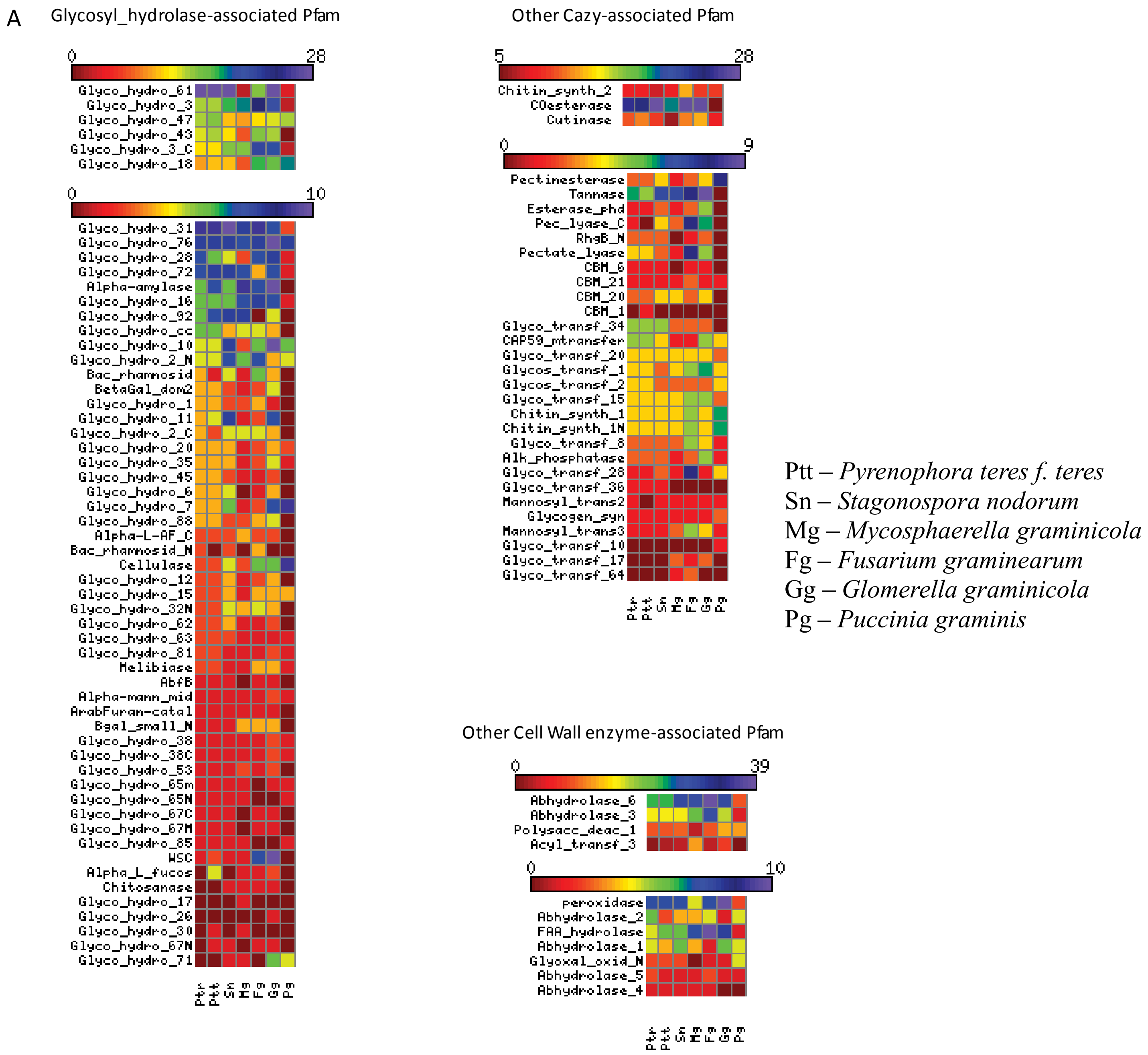
A
Glycosyl_hydrolase-associated Pfam
0
28
Glyco_hydro_61
Glyco_hydro_3
Glyco_hydro_47
Glyco_hydro_43
Glyco_hydro_3_C
Glyco_hydro_18
0
10
Glyco_hydro_31
Glyco_hydro_76
Glyco_hydro_28
Glyco_hydro_72
Alpha-amylase
Glyco_hydro_16
Glyco_hydro_92
Glyco_hydro_cc
Glyco_hydro_10
Glyco_hydro_2_N
Bac_rhamnosid
BetaGal_dom2
Glyco_hydro_1
Glyco_hydro_11
Glyco_hydro_2_C
Glyco_hydro_20
Glyco_hydro_35
Glyco_hydro_45
Glyco_hydro_6
Glyco_hydro_7
Glyco_hydro_88
Alpha-L-AF_C
Bac_rhamnosid_N
Cellulase
Glyco_hydro_12
Glyco_hydro_15
Glyco_hydro_32N
Glyco_hydro_62
Glyco_hydro_63
Glyco_hydro_81
Melibiase
AbfB
Alpha-mann_mid
ArabFuran-catal
Bgal_small_N
Glyco_hydro_38
Glyco_hydro_38C
Glyco_hydro_53
Glyco_hydro_65m
Glyco_hydro_65N
Glyco_hydro_67C
Glyco_hydro_67M
Glyco_hydro_85
WSC
Alpha_L_fucos
Chitosanase
Glyco_hydro_17
Glyco_hydro_26
Glyco_hydro_30
Glyco_hydro_67N
Glyco_hydro_71
Ptr Ptt Sn Mg Fg Gg Pg
Other Cazy-associated Pfam
5
28
Chitin_synth_2
COesterase
Cutinase
0
9
Pectinesterase
Tannase
Esterase_phd
Pec_lyase_C
RhgB_N
Pectate_lyase
CBM_6
CBM_21
CBM_20
CBM_1
Glyco_transf_34
CAP59_mtransfer
Glyco_transf_20
Glycos_transf_1
Glycos_transf_2
Glyco_transf_15
Chitin_synth_1
Chitin_synth_1N
Glyco_transf_8
Alk_phosphatase
Glyco_transf_28
Glyco_transf_36
Mannosyl_trans2
Glycogen_syn
Mannosyl_trans3
Glyco_transf_10
Glyco_transf_17
Glyco_transf_64
Ptr Ptt Sn Mg Fg Gg Pg
Ptt – Pyrenophora teres f. teres
Sn – Stagonospora nodorum
Mg – Mycosphaerella graminicola
Fg – Fusarium graminearum
Gg – Glomerella graminicola
Pg – Puccinia graminis
Other Cell Wall enzyme-associated Pfam
0
39
Abhydrolase_6
Abhydrolase_3
Polysacc_deac_1
Acyl_transf_3
0
10
peroxidase
Abhydrolase_2
FAA_hydrolase
Abhydrolase_1
Glyoxal_oxid_N
Abhydrolase_5
Abhydrolase_4
Ptr Ptt Sn Mg Fg Gg Pg

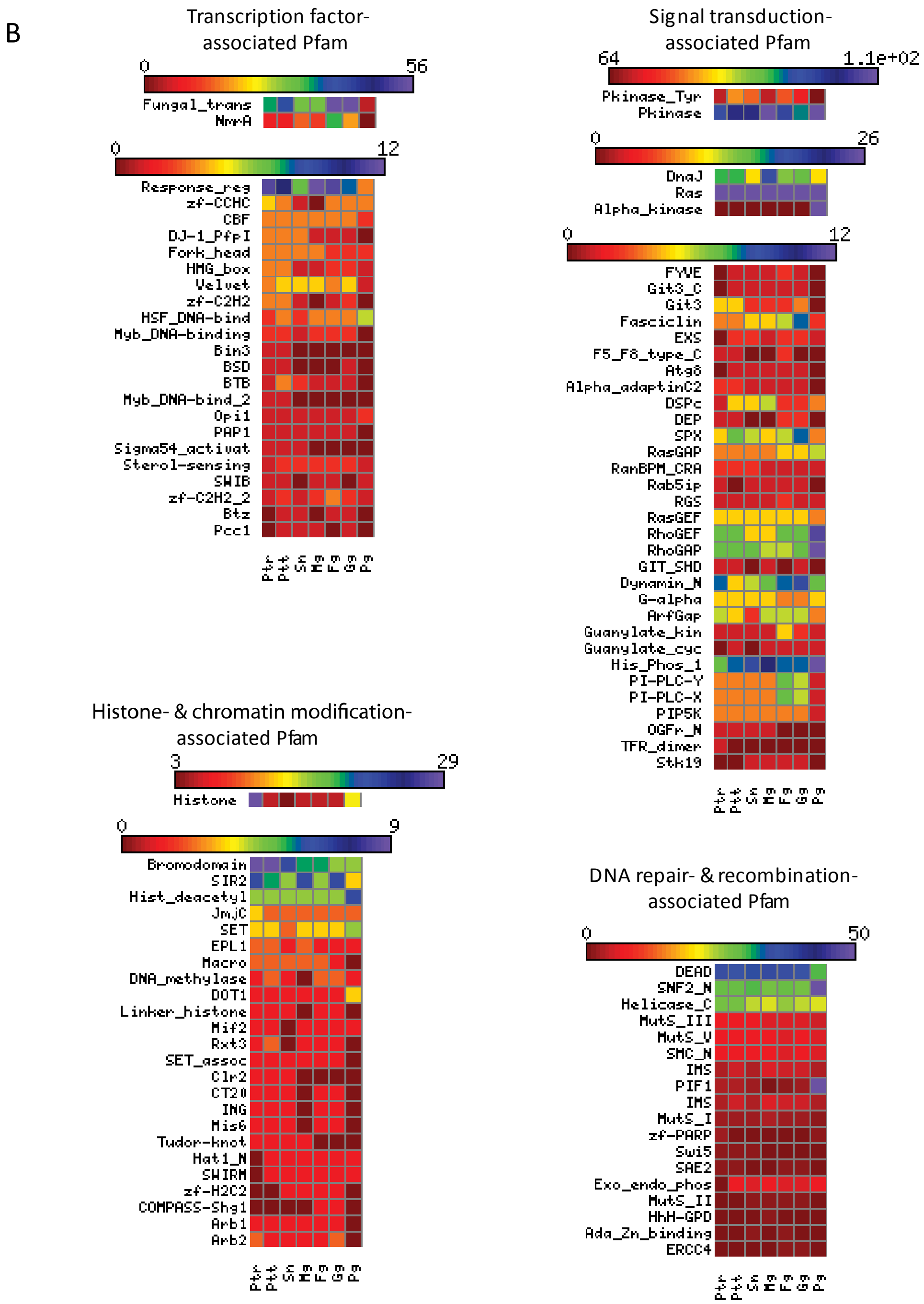
B
Transcription factor-associated Pfam
0
56
Fungal_trans
NmrA
0
12
Response_reg
zf-CCHC
CBF
DJ-1_PfpI
Fork_head
HMG_box
Velvet
zf-C2H2
HSF_DNA-bind
Myb_DNA-binding
Bin3
BSD
BTB
Myb_DNA-bind_2
Opi1
PAP1
Sigma54_activat
Sterol-sensing
SWIB
zf-C2H2_2
Btz
Pcc1
Ptr Ptt Sn Mg Fg Gg Pg
Signal transduction-associated Pfam
64
1.1e+02
Pkinase_Tyr
Pkinase
0
26
DnaJ
Ras
Alpha_kinase
0
12
FYVE
Git3_C
Git3
Fasciclin
EXS
F5_F8_type_C
Atg8
Alpha_adaptinC2
DSPc
DEP
SPX
RasGAP
RanBPM_CRA
Rab5ip
RGS
RasGEF
RhoGEF
RhoGAP
GIT_SHD
Dynamin_N
G-alpha
ArfGap
Guanylate_kin
Guanylate_cyc
His_Phos_1
PI-PLC-Y
PI-PLC-X
PIP5K
OGFr_N
TFR_dimer
Stk19
Ptr Ptt Sn Mg Fg Gg Pg
Histone- & chromatin modification-associated Pfam
3
29
Histone
0
9
Bromodomain
SIR2
Hist_deacetyl
JmjC
SET
EPL1
Macro
DNA_methylase
DOT1
Linker_histone
Mif2
Rxt3
SET_assoc
Clr2
CT20
ING
Mis6
Tudor-knot
Hat1_N
SWIRM
zf-H2C2
COMPASS-Shg1
Arb1
Arb2
Ptr Ptt Sn Mg Fg Gg Pg
DNA repair- & recombination-associated Pfam
0
50
DEAD
SNF2_N
Helicase_C
MutS_III
MutS_V
SMC_N
IMS
PIF1
IMS
MutS_I
zf-PARP
Swi5
SAE2
Exo_endo_phos
MutS_II
HhH-GPD
Ada_Zn_binding
ERCC4
Ptr Ptt Sn Mg Fg Gg Pg

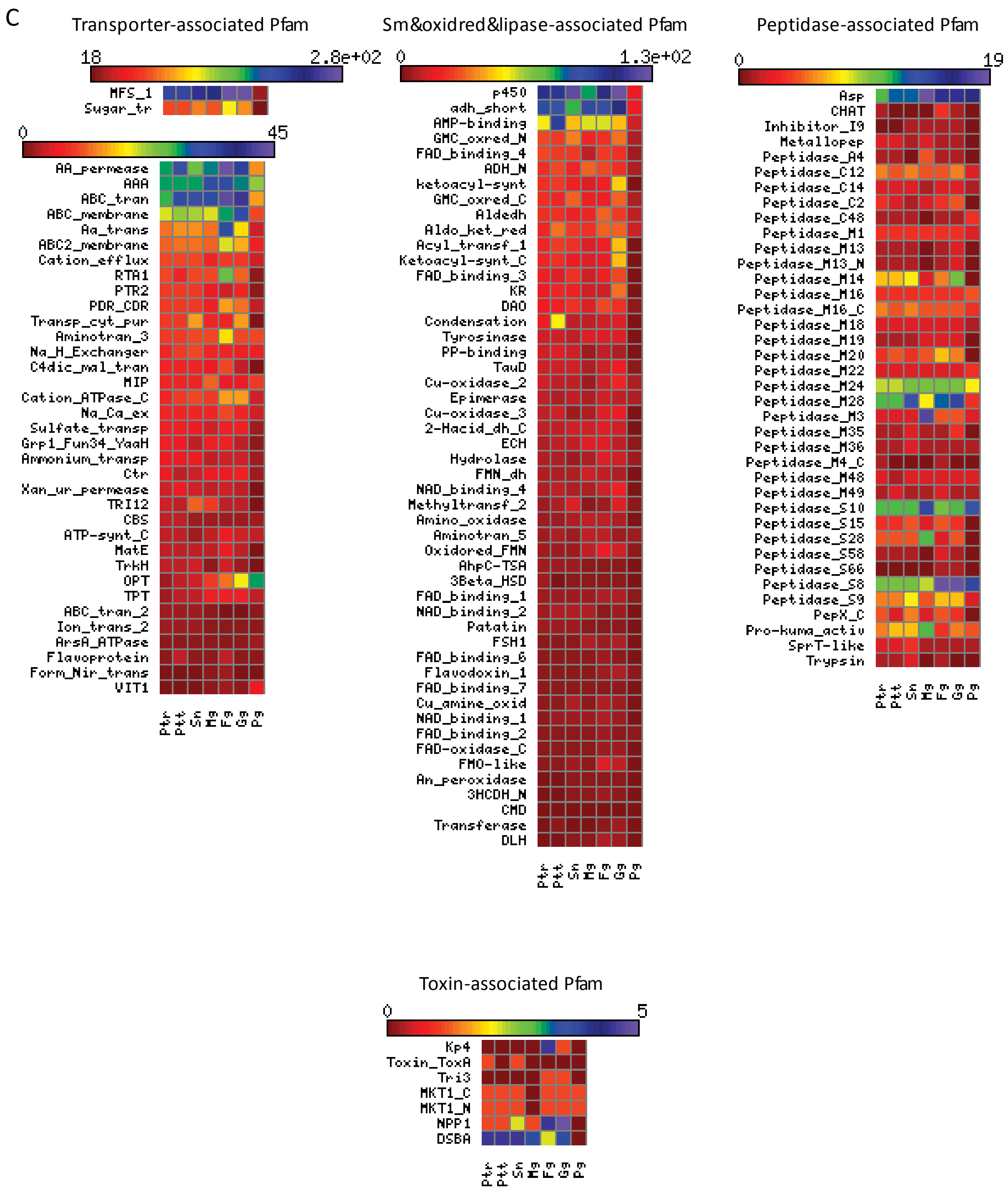

Figure S2 Heat map of Pfam domains for (A) carbohydrate active enzymes and other cell wall enzymes, (B) proteins involved in sensing and response (C) proteins associated with transport, oxidative stress and toxin activity present in *Ptr* and other cereal pathogens.
