# Supplementary material for: Comparative Genomics of a Plant-Pathogenic Fungus, Pyrenophora tritici-repentis, Reveals Transduplication and the Impact of Repeat Elements on Pathogenicity and Population Divergence
Source: G3 (Bethesda). 2013 Jan 1;3(1):41–63. doi: 10.1534/g3.112.004044 (PMC3538342; doi:10.1534/g3.112.004044)
Supplement: Supporting Information [file supp_3.1.41_FigureS3.pdf]

```
PTRG_03457     CGTCATGCAGTCCGTGCGCTGCTGCCTTTACTAGTGCGGAAATCGGGAAATTGCCGTCGCCCAAACTTTCCAGCTACCTAACTCTTTCAGCCTACTACTT
DW7_94         CGTCATGCAGTCCGTGCGCTGCTGCCTTTACTAGTGCGGAAATCGGGAAATTGCCGTCGCCCAAACTTTCCAGCTACCTAACTCTTTCAGCCTACTACTT
DW7_14025      CGTTATGCAGTCCGTGCGCTGCTGCCTTTACTAGTGCGGAAATCGGGAAATTGCCGTCGCCCAAACTTTCCAGCTACCTAACTCTTTCAGCCTACTACTT
PTRG_00559                                                                            AGCTACCTAACTCTTTCAGCCTACTACTT
SD20_20175                                                                                        CTTTCAGCCTACTACTT

DW7_94         TCTTAATTGTCGACCACATTTATCACAGTAATTAATCACATACGTCGCCTACGTTCATCATCTACTCTACTACGCAATGGCAAGGACAAAACCACGGCCT
DW7_14025      TCTTAATTGTCGACCACATTGATTACAGTAATTAATTACATACGTCGCCTACGTTTATCATCTACTCCACTACGCAATAGCAAGGACAAAACCACGGCCT
PTRG_00559     TCTTAATTGTCGACCACATTTATCACAGTAATTAATCACATACGTCGCCTACGTTCATCATCTACTCTACTACGCAATGGCAAGGACAAAACCACGGCCT
SD20_20175     TCTTAATTGTCGACCACATTTATTACAGTAATTAATTACATACGTCGCCTACATTTATCATCTACTCTACTACGCAATAGCAAGGACAAAACCACGGCCT
PTRG_03457     TCTTAATTGTCGACCACATTTATCACAGTAATTAATCACATACGTCGCCTACGTTCATCATCTACTCTACTACGCAATGGCAAGGACAAAACCACGGCCT

DW_94          AAGGTCACCGGTAAAGCCG
DW_14025       AAGGTCACCGGTAAAGCCG
PTRG_00559     AAGGTCACCGGTAAAGCCGGCCGGGGTGGTCCTGATGGCAAGACAGTTACTGGCGGCAAGCCGGCTCAGAAGGCCCTTGCTAGTAAGGCTAGACGTCAAG
SD20_20175     AAGGTCACCGGTAAAGCCGGCCGGGGTGGTCCTGATGGCAAGACAGTTACTGGCGGCAAGCCGGCTCAGAAGGCCCTTGCTAGTAAGGCTAGACGTCAAG
PTRG_03457     AAGGTCACCGGTAAAGCCGGCCGGGGTGGTCCTGATGGCAAGACAGTTACTGGCGGCAAGCCGGCTCAGAAGGCCCTTGCTAGTAAGGCTAGACGTCAAG

PTRG_00559     TAGCAGGAAAGTCTACGCGAAAGGCACCTGTAGCTGTTAAGAAGAAGCGCAAGTTTAAGGCCGGCAgtaggttatcacagtcgtttatcacagctatttt
SD20_20175     TAGCAGGAAAGTCTACGCGAAAGGCACCTGTAGCTGTTAAGAAGAAGCGCAAGTTTAAGGCCGGCAgtaggttatcacagttgtttattacagctatttt
DW7_11351                                               AAGAAGCGCAAGTTTAAGGCCGGCAgtaggttatcacagttgtttattacagctatttt
PTRG_03457     TAGCAGGAAAGTCTACGCGAAAGGCACCTGTAGCTGTTAAGAAGAAGCGCAAGTTTAAGGCCGGCAgtaggttatcacagtcgtttatcacagctatttt

PTRG_00559     ctaatttagcttagCTGTCGCATTACGGGAAATCAAGAGATACCAGAGAGGTTTTGAACTACTCTTGCGAAAACTCCCCTTTTCCCGCGTAGTGCGCGAA
SD20_20175     ctagataagcttagCTGTCGCATTACGGGAAATTAAGAGATACCAGAGAGGTTTTAAACTACTCTTGCGAAAACTCCCCTTTTCCCGCGTAGTGCGCGAA
DW7_11351      ctaatcaagcttagCTGTCGCATTACTGAAAATTAAGAGATACCAGAGAGGTTTTAAACTACTCTTGCGAAAACTCCCCTTTTCCCGCGTAGTGCGCGAA
PTRG_03457     ctaatttagcttagCTGTCGCATTACGGGAAATCAAGAGATACCAGAGAGGTTTTGAACTACTCTTGCGAAAACTCCCCTTTTCCCGCGTAGTGCGCGAA

PTRG_00559     TTTGCACAGGTGCACAAGGCCGATATCCGCTTTCAACGATCTGCAATCGAAGCTCTTCAGGAAGCTACAGAAGCTTTCTTAGTTGGTTATTTTgagggta
SD20_20175     TTTGCACAGGTGCACAAGGCCGATATCCGCTTTCAACGATCTGCAATCGAAGCTCTTCAGGAAGCTACAGAAGCTTTCTTAGTTACTTATTTTaagggta
DW7_11351      TTTGCACAGGTGCACAAGGCCGATATCCGCTTTTAACGATCTGCAATCGAAGCTCTTCAGGAAGCTACATAAGCTTTCTTAGTTAGTTATTTTaagggta
PTRG_03457     TT-GCACAGGTGCACAAGGCCGATATCCGCTTTCAACGATCTGCAATCGAAGCTCTTCAGGAAGCTACAGAAGCTTTCTTAGTTGGTTATTTTgagggta

PTRG_00559     tagtctatctaccctatactatttttcatttaataacttttatagACTGCAACATCAATGCTATTCACGCAAAGAGGGTTACTATTCAAGAGAAGGATTC
SD20_20175     taatctatctaccctatactatttttcatttaataacttttatagACTGCAACATCAATGCTATTCACGCAAAGAGGGTTACTATTCAAGAGAAGGATTC
DW7_11351      tgatctatctatcctatactatttttcatttaataacttttatagACTGCAACATCAATGCTATTCACGCAAAGAGGGTTACTATTCAAGAGAAGGATTC
PTRG_03457     tagtctatctaccctatactatttttcatttaataacttttatagACTGCAACATCAATGCTATTCACGCAAAGAGGGTTACTATTCAAGAGAAGGATTC

PTRG_00559     TCAATTGGCTAGGCGCTACTTTGCGCGCGAGTTACTAGCTTTTCTCTAGATTATAAGATATATCTAGATACACGTGCTTAAATGCTAGCTGGCGAAGCCT
SD20_20175     TCAATTGGCTAGGCGCTACTTTGCGCGCGAGTTACTAGCTTTTCTCTAGATTATAAGATATATCTAGATACACGTGCTTAAATGCTAGCTGGCGAAGCCT
DW7_11351      TCAATTAGCTAGGCGCTACTTTGCGCGCGAGTTAATAGCTTTTCTCTAGATTATAAGATATATCTAGATACATGTGCTTAAATGGTAGCTGGCGAAGCCT
PTRG_03457     TCAATTGGCTAGGCGCTACTTTGCGCGCGAGTTACTAGCTTTTCTCTAGATTATAAGATATATCTAGATACACGTGCTTAAATGCTAGCTGGCGAAGCCT

PTRG_00559     CTGTATATTATAAATGAAACTAGTTAATGCTACACTG
SD20_20175     CTGTATATTATAAATGAAACTAGTTAATGCTACACTGTGTTAATCTACTG
DW7_11351      CTGTATAGTATAAATGAAACTAGTTAATGCTACACTGTAATAATCTACTGTGATACTATATAAACTTTTCTTTTATTGAAATCTATATTATATATCTTGC
PTRG_03457     CTGTATATTATAAATGAAACTAGTTAATGCTACACTG
```

**Figure S3** Alignment of novel H3-like (H3L) genes identified in three different *Ptr* isolates (PTRG numbers refer to genes identified in the reference genome of BFP-ToxAC, while DW7 and SD20 refer to the pathogenic DW7-ToxB and non-pathogenic SD20-NP strains, respectively). In all cases, contigs assembled *de novo* from Illumina reads suggest that copies of H3L genes in both DW7-ToxB and SD20-NP are inactive (note the many point mutations when compared to the PTRG sequences). DW7_14025 and SD20_20175 in particular lack a proper start codon (ATG to ATA change, underlined), while DW7_11351 contains several in-frame nonsense codons in addition to the conserved stop codon (underlined).
