# Supplementary material for: Comparative Genomics of a Plant-Pathogenic Fungus, Pyrenophora tritici-repentis, Reveals Transduplication and the Impact of Repeat Elements on Pathogenicity and Population Divergence
Source: G3 (Bethesda). 2013 Jan 1;3(1):41–63. doi: 10.1534/g3.112.004044 (PMC3538342; doi:10.1534/g3.112.004044)
Supplement: Supporting Information [file supp_3.1.41_FigureS4.pdf]

A

```
Ptr H3           1 MARTKQTARKSTGGKAPRKQLASKAARKSAPSTGGVKKPHRYKPGTVALREIRRYQKSTE
Sn H3            1 MARTKQTARKSTGGKAPRKQLASKAARKSAPSTGGVKKPHRYKPGTVALREIRRYQKSTE
NcH3             1 MARTKQTARKSTGGKAPRKQLASKAARKSAPSTGGVKKPHRYKPGTVALREIRRYQKSTE
ScH3             1 MARTKQTARKSTGGKAPRKQLASKAARKSAPSTGGVKKPHRYKPGTVALREIRRFQKSTE
SpH3             1 MARTKQTARKSTGGKAPRKQLASKAARKAAPATGGVKKPHRYRPGTVALREIRRYQKSTE
HsH3.1           1 MARTKQTARKSTGGKAPRKQLATKAARKSAPATGGVKKPHRYRPGTVALREIRRYQKSTE
HsH3.3           1 MARTKQTARKSTGGKAPRKQLATKAARKSAPSTGGVKKPHRYRPGTVALREIRRYQKSTE

SNOG_03319      61 LLIRKLPFQRLVREIAQDFKSDLRFQSSAIGALQESVEAYLVSLFEDTNLCAIHAKRVTI
PTRG_11558.1    61 LLIRKLPFQRLVREIAQDFKSDLRFQSSAIGALQESVEAYLVSLFEDTNLCAIHAKRVTI
NcH3            61 LLIRKLPFQRLVREIAQDFKSDLRFQSSAIGLLQESVESYLVSLFEDTNLCAIHAKRVTI
ScH3            61 LLIRKLPFQRLVREIAQDFKTDLRFQSSAIGALQESVEAYLVSLFEDTNLAAIHAKRVTI
SpH3            61 LLIRKLPFQRLVREIAQDFKTDLRFQSSAIGALQEAVEAYLVSLFEDTNLCAIHGKRVTI
HsH3.1          61 LLIRKLPFQRLVREIAQDFKTDLRFQSSAVMALQEACEAYLVGLFEDTNLCAIHAKRVTI
HsH3.3          61 LLIRKLPFQRLVREIAQDFKTDLRFQSAAIGALQEASEAYLVGLFEDTNLCAIHAKRVTI

SNOG_03319     121 QSKDIQLARRLRGERG
PTRG_11558.1   121 QSKDIQLARRLRGERG
NcH3           121 QSKDIQLARRLRGERN
ScH3           121 QKKDIKLARRLRGERS
SpH3           121 QPKDMQLARRLRGERS
HsH3.1         121 MPKDIQLARRIRGERA
HsH3.3         121 MPKDIQLARRIRGERA
```

B

```
SNOG_15370    1 MAPKTPDRRNKARPSRGGRAS-------TGSRRESGKRVGQAGPAPDRAKKRYKPGTVAL
PtrCenH3      1 MAPKSPARRNKARPSRGGRVS-------VGSRKESGKRLGQPGPAPDRAKKRYKPGTVAL
Sc_Cse4       1 METEVPA------PVRTHSYA-------LDRYVRQKRREKQRKQSLKRVEKKYTPSELAL
Nc_CenH3      1 MPPKKGGVTKSKAVSKKAAAVPTPKATPPGRRKSRASSVQPGDPVPQGKKRRYRPGTLAL
Sp_Cnp1       1 -------------MAKKSLMA------------------EPGDPIPRPRKKRYRPGTTAL
MmCENpA       1 MGPRR---KPQTPRRR-PS-S------------PAPGPSRQSSSVGSQTLRR-RQKFMWL
HsCENpA       1 MGPRRRSRKPEAPRRRSPSPT------------PTPGPSRRGPSLGASSHQHSRRRQGWL

SNOG_15370   54 REIRRYQKSTDLLLLRTPFQRLVREIAQTVTT---ETGPTRWQSQAIQALQEATEAFLVN
PtrCenH3     54 REIKRYQKTTDLLLLKLPFQRLVREIAQSVTT---EDGPNRWQSQAIMALQEATEAFLVN
Sc_Cse4      48 YEIRKYQRSTDLLISKIPFARLVKEVTDEFTT---KDQDLRWQSMAIMALQEASEAYLVG
Nc_CenH3     61 KEIRNYQRTTDLLVAKLPFARLVREIAMQFRP---MDEEMRWQSQAILALQEAAEAFLVH
Sp_Cnp1      30 REIRKYQRSTDLLIQRLPFSRIVREISSEFVANFSTDVGLRWQSTALQCLQEAAEAFLVH
MmCENpA      43 KEIKTLQKSTDLLFRKKPFSMVVREICEKFSR----GVDFWWQAQALLALQEAAEAFLIH
HsCENpA      49 KEIRKLQKSTHLLIRKLPFSRLAREICVKFTR----GVDFNWQAQALLALQEAAEAFLVH

SNOG_15370  111 LFHDANLCAIHAKRVTIQQKDIQLARRLRAAWGAPV--
PtrCenH3    111 LFHDANLCAIHAKRVTIQQKDIQLARRLRAAWGAPV--
Sc_Cse4     105 LLEHTNLLALHAKRITIMKKDMQLARRIRGQFI-----
Nc_CenH3    118 LFEDTNLCAIHAKRVTIMQKDIQLARRIRGVWGGAGWV
Sp_Cnp1      90 LFEDTNLCAIHAKRVTIMQRDMQLARRIRGA-------
MmCENpA      99 LFEDAYLLSLHAGRVTLFPKDIQLTRRIRGFEGGLP--
HsCENpA     105 LFEDAYLLTLHAGRVTLFPKDVQLARRIRGLEEGLG--
```

Figure S4 Alignment of *bona fide* histone H3 proteins from *Ptr* with those of other fungi reveal the expected level of conservation. (A) Histone H3 is extremely well conserved among *Ptr*, *S. nodorum* (Sn), *N. crassa* (Nc), *Schizosaccharomyces pombe* (Sp), *Saccharomyces cervisiae* (Sc) and strongly resembles the replication-dependent H3.1 or replication-independent H3.3 from human (Hs). This is in stark contrast to alignments with the novel family of H3L proteins identified in *Ptr* (see Figure 3B). (B) Centromere-specific variants of H3, called CenH3 or CenpA, have variable N-terminal regions and more conserved histone-fold and C-terminal domains. While this variability suggested that H3Ls may be similar to CenH3 proteins, alignments of the histone-fold domain suggest that H3Ls are derived from canonical H3s (see Figure 3B). In both figures, completely conserved residues are shown in black, similar residues are in green and variable residues are in red.
