# Supplementary material for: Comparative Genomics of a Plant-Pathogenic Fungus, Pyrenophora tritici-repentis, Reveals Transduplication and the Impact of Repeat Elements on Pathogenicity and Population Divergence
Source: G3 (Bethesda). 2013 Jan 1;3(1):41–63. doi: 10.1534/g3.112.004044 (PMC3538342; doi:10.1534/g3.112.004044)
Supplement: Supporting Information [file supp_3.1.41_FigureS5.pdf]

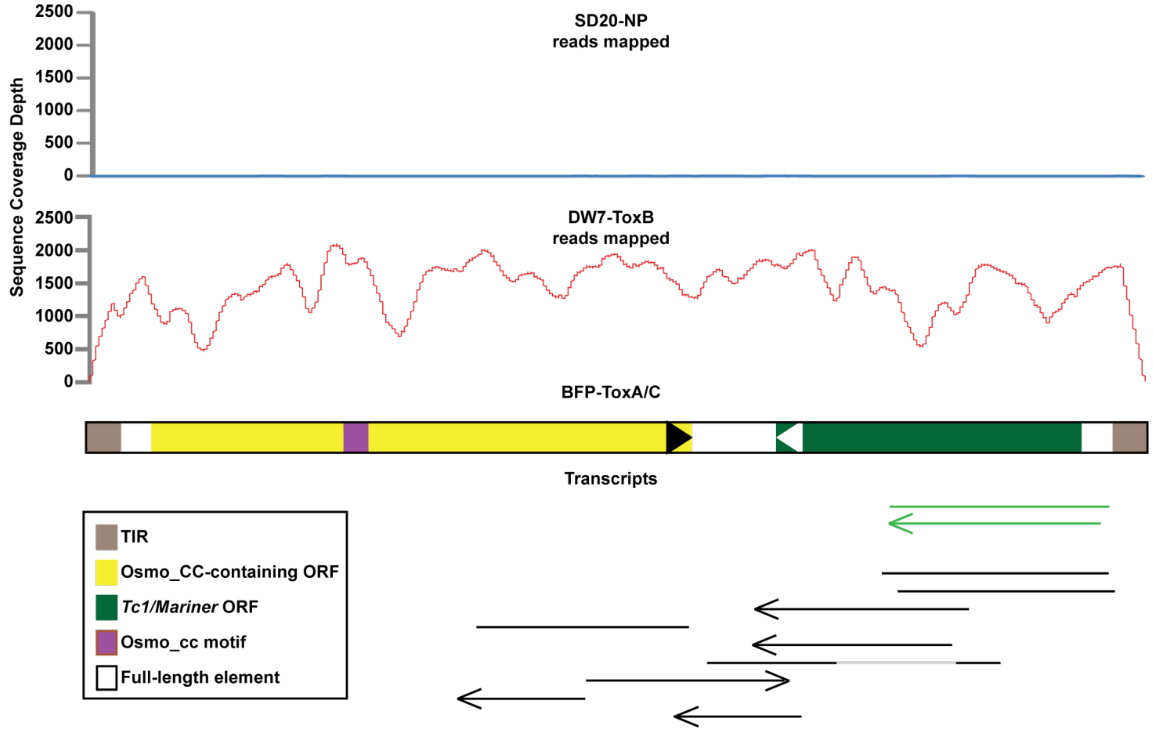

Figure S5 Transduplication of osmosensory transporter coiled-coil (Osmo-CC) domain in *Ptr*. The bar represents a full length element (3.4-kb) of this repeat family present in the reference genome of BFP-ToxAC. The graphs on the top are a result of mapping of all Illumina sequencing reads from each isolate to the reference and represents the sequence depth at that position. Arrows and lines below the element representation indicate alignments of transcripts present in various libraries (arrows - polyA tails, light gray - introns).
