# Supplementary material for: Comparative Genomics of a Plant-Pathogenic Fungus, Pyrenophora tritici-repentis, Reveals Transduplication and the Impact of Repeat Elements on Pathogenicity and Population Divergence
Source: G3 (Bethesda). 2013 Jan 1;3(1):41–63. doi: 10.1534/g3.112.004044 (PMC3538342; doi:10.1534/g3.112.004044)
Supplement: Supporting Information [file supp_3.1.41_FigureS6.pdf]

A. Putative Histone deacetylase inhibitor cluster in ***P. tritici-repentis***

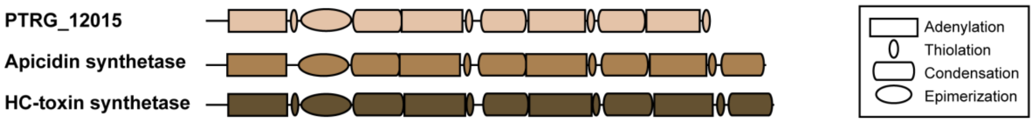

B. Tox2-like cluster of ***P. tritici-repentis***

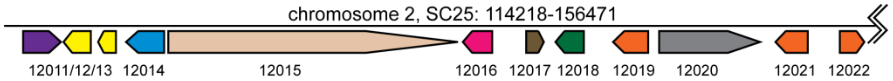

P. tritici-repentis		Best BlastP hit			
**Locus**	**Proposed function**	**Accession Number**	**Organism**	**Annotation**	**e value**
12011	FAD-linked oxidoreductase	ACZ66255.1	*F. incarnatum*	APS9	9e-160
		XP_001209092.1	*A. terreus*	conserved hypothetical	4e-104
12012	Short chain dehydrogenase	XP_965074.1	*N. crassa*	conserved hypothetical	8e-80
		XP_001239054.1	*C. immitis*	conserved hypothetical	1e-77
12013	Short chain dehydrogenase	XP_001820062.1	*A. oryzae*	hypothetical protein	3e-68
		XP_002374381.1	*A. flavus*	short chain type dehydrogenase, putative	9e-68
12014	MFS transporter	ACZ66257.1	*F. incarnaturm*	APS11	1e-123
		sp:Q00357	*C. carbonum*	HC-toxin efflux carrier	2e-102
12015	NRPS	ACZ66258.1	*F. incarnatum*	APS1	0.0
		sp:Q01886.2	*C. carbonum*	HC-toxin synthetase	0.0
12016	Basic leucine zipper transcription factor	ACZ66248.1	*F. incarnatum*	APS2	2e-26
		sp:O74205	*C. carbonum*	Transcription factor ToxE	2e-16
12017	Thioesterase	XP_001799397.1	*S. nodorum*	hypothetical protein SNOG_09095	2e-24
		XP_002378766.1	*A. flavus*	hypothetical protein	1e-23
12018	Aminotransferase	sp:Q9Y885.1	C. carbonum	Aminotransferase ToxF	9e-123
		ACZ66250.1	*F. incarnatum*	APS4	6e-105
12019	cytochrome P450	XP_001555869.1	*B. fuckeliana*	hypothetical protein	4e-105
		XP_001795303	*S. nodorum*	hypothetical protein	1e-96
12020	Fatty acid synthase	ACZ66251.1	*F. incarnatum*	APS5	0.0
		XP_002146311.1	*P. marneffei*	3-oxoacyl-[acyl-carrier-protein] synthase	0.0
12021	cytochrome P450	XP_001275091.1	*A. clavatus*	benzoate 4-monooxygenase, cytochrome P450	3e-134
		ACZ66253.1	*F. incarnatum*	APS7	8e-125
12022	cytochrome P450	ACZ66254.1	*F. incarnatum*	APS8	2e-109
		XP_002477830.1	*T. stipitatus*	cytochrome P450, putative	2e-76

Figure S6 Modular architecture and biosynthetic cluster of a putative histone deacetylase inhibitor biosynthetic cluster in *P. tritici-repentis.* (A) Modular architecture of PTRG_12015, APS1 (Apicidin synthetase) and HTS1 (HC-toxin synthetase). Domains (Adenylation, Thiolation, Condensation, and Epimerization) and spacing are to scale. (B) Putative biosynthetic cluster containing PTRG_12015. Putative open reading frames are designated as arrows. Proteins with similar function are coded with a similar color. Blastp search was performed against the NCBI nr database. Proposed function as determined by KOG or GO classification.
