# Supplementary material for: Comparative Genomics of a Plant-Pathogenic Fungus, Pyrenophora tritici-repentis, Reveals Transduplication and the Impact of Repeat Elements on Pathogenicity and Population Divergence
Source: G3 (Bethesda). 2013 Jan 1;3(1):41–63. doi: 10.1534/g3.112.004044 (PMC3538342; doi:10.1534/g3.112.004044)
Supplement: Supporting Information [file supp_3.1.41_FigureS7.pdf]

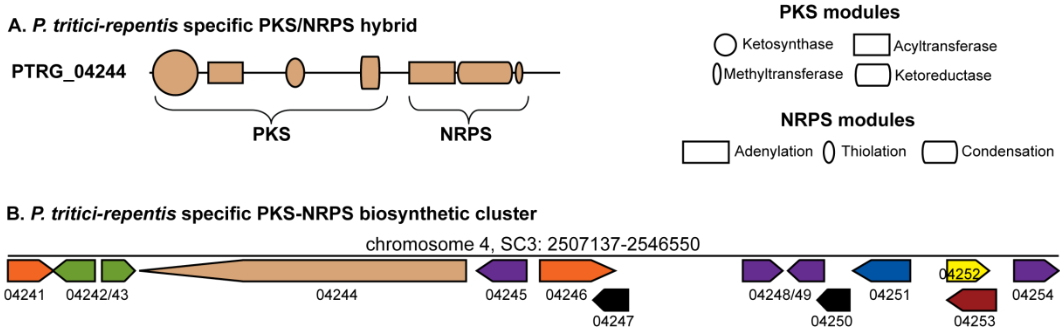

P. tritici-repentis		Best BlastP hit			
**Locus**	**Proposed function**	**Accession Number**	**Organism**	**Annotation**	**e value**
04241	cytochrome p450	gb|EEQ33486.1	*M. canis*	n-alkane inducible cytrochrome P450	9e-168
04242	Flavoprotein mono-oxygenase	XP_001801158.1	*S. nodorum*	conserved hypothetical	3e-109
04243	2OG-Fe(II) oxygenase	XP_001905187.1	*P. anserina*	unnamed protein product	3e-68
04244	PKS:NPS hybrid	XP_001905191.1	*P. anserina*	unnamed protein product	0.0
04245	FAD-linked oxidase	XP_391370.1	*F. graminearum*	hypothetical protein	1e-162
04246	cytochrome p450	XP_002487173.1	*T. stipitatus*	cytochrome P450 monooxigenase	2e-120
04247	predicted protein				
04248	alchohol oxidase	gb|EEU33852	*N. haematococca*	hypothetical protein	2e-117
04249	oxidoreductase	XP_002479371.1	*T. stipitatus*	oxidoreductase, putative	1e-162
04250	hypothetical protein	gb|EEU35594.1	*N. haematococca*	hypothetical protein	4e-08
04251	MFS efflux pump	XP_002341986.1	*T. stipitatus*	efflux pump antibiotic resistance protein, putative	2e-136
04252	putative short chain dehydrogenase	XP_001229641.1	*C. globosum*	hypothetical protein	2e-66
04253	O-methyltransferase	XP_001550242.1	*B. fuckeliana*	hypothetical protein	2e-66
04254	FAD-linked oxidase	XP_002483865.1	*T. stipitatus*	FAD-dependent isoamyl alcohol oxidase, putative	6e-122

Figure S7 Modular architecture and biosynthetic cluster of a *P. tritici-repentis*-specific PKS/NRPS hybrid. (A) Modular architecture of PTRG_04244. Domains and spacing are to scale. (B) Putative biosynthetic cluster containing PTRG_04244. Putative proteins are designated as arrows. Proteins with similar function are coded with a similar color. Blastp search against GenBank nr database. Proposed function as determined by KOG or GO classification.
