# Supplementary material for: Comparative Genomics of a Plant-Pathogenic Fungus, Pyrenophora tritici-repentis, Reveals Transduplication and the Impact of Repeat Elements on Pathogenicity and Population Divergence
Source: G3 (Bethesda). 2013 Jan 1;3(1):41–63. doi: 10.1534/g3.112.004044 (PMC3538342; doi:10.1534/g3.112.004044)
Supplement: Supporting Information [file supp_3.1.41_FigureS8.pdf]

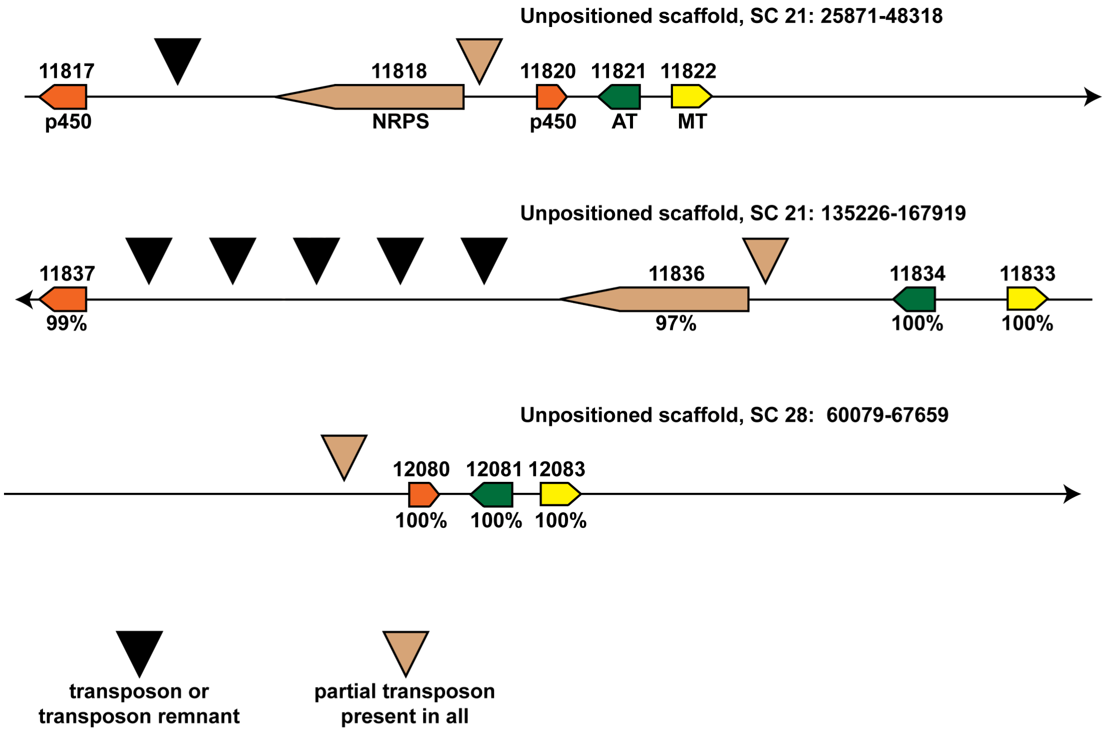


**Figure S8** Duplicated genomic regions associated with the NRPSs PTRG_11818 & 11836. Putative proteins are designated as arrows. Proteins with similar function are coded with similar color. AT - -acyltransferase; MT - methyltransferase. Protein coding regions and spacing between are to scale. Numbers above boxes indicate locus postion in the reference. Numbers beneath boxes represent the % identity of the proteins relative to PTRG_11817 through 11822. Triangles represent transposons or transposon remnants.
