# Supplementary material for: Comparative Genomics of a Plant-Pathogenic Fungus, Pyrenophora tritici-repentis, Reveals Transduplication and the Impact of Repeat Elements on Pathogenicity and Population Divergence
Source: G3 (Bethesda). 2013 Jan 1;3(1):41–63. doi: 10.1534/g3.112.004044 (PMC3538342; doi:10.1534/g3.112.004044)
Supplement: Supporting Information [file supp_3.1.41_TableS1.pdf]

Table S1 ***P. tritici-repentis*** **isolates used in this study**

Isolate name	Race	Geographic location	Path/non-path	Analyses
BFP-ToxA/C	1	S. Dakota, USA	Path	Reference Genome/EST/PCR
ASC1	1	Manitoba, Canada	Path	PCR
86-124	2	Manitoba, Canada	Path	PCR
D308	3	Manitoba, Canada	Path	PCR
DW2	5	N. Dakota, USA	Path	PCR
DW7	5	N. Dakota, USA	Path	Resequencing
SO3	?	Oregon, USA	Path	EST/PCR
SD20-NP	4	S. Dakota, USA	Non-path	Resequencing/EST/PCR
90-2	4	Manitoba, Canada	Non-path	PCR
98-31-2	4	N. Dakota, USA	Non-path	PCR
