# Supplementary material for: Comparative Genomics of a Plant-Pathogenic Fungus, Pyrenophora tritici-repentis, Reveals Transduplication and the Impact of Repeat Elements on Pathogenicity and Population Divergence
Source: G3 (Bethesda). 2013 Jan 1;3(1):41–63. doi: 10.1534/g3.112.004044 (PMC3538342; doi:10.1534/g3.112.004044)
Supplement: Supporting Information [file supp_3.1.41_TableS2.pdf]

Table S2 ***P. tritici-repentis*** **reference genome libraries sequenced**

Library	Reads	Physical Coverage (Fold)	Sequence Coverage * (Fold)
4kb Plasmid	266,377	14	4.73
10kb Plasmid	56,616	9	1.11
40kb Fosmid	64,483	35	1.09
**Total**	**387,476**	**58**	**6.93**

* Q20 base coverage
