# Supplementary material for: Comparative Genomics of a Plant-Pathogenic Fungus, Pyrenophora tritici-repentis, Reveals Transduplication and the Impact of Repeat Elements on Pathogenicity and Population Divergence
Source: G3 (Bethesda). 2013 Jan 1;3(1):41–63. doi: 10.1534/g3.112.004044 (PMC3538342; doi:10.1534/g3.112.004044)
Supplement: Supporting Information [file supp_3.1.41_TableS3.pdf]

Table S3 ***P. tritici-repentis*** **assembly anchored to the optical maps**

Optical Linkage group	Estimated Size (Mb)	Mapped Scaffolds	Scaffold Size (Mb)
chr1	10.166	10, 33, 18,1,17	9.504
chr2	5.206	16,2,25,23	4.322
chr3	3.708	12,14,13	3.361
chr4	3.301	3	3.079
chr5	3.151	5	2.677
chr6	2.951	4	2.787
chr7	2.753	30,15,11	2.612
chr8	2.391	8,24,26	2.115
chr9	2.287	9,22,27	2.026
chr10	2.204	6	1.985
chr11	2.113	7	1.888
**Total**	**40.231**		**36.355**
