# Supplementary material for: Comparative Genomics of a Plant-Pathogenic Fungus, Pyrenophora tritici-repentis, Reveals Transduplication and the Impact of Repeat Elements on Pathogenicity and Population Divergence
Source: G3 (Bethesda). 2013 Jan 1;3(1):41–63. doi: 10.1534/g3.112.004044 (PMC3538342; doi:10.1534/g3.112.004044)
Supplement: Supporting Information [file supp_3.1.41_TableS4.pdf]

Table S4 Illumina sequenced *P. tritici-repentis* isolate read alignment under different stringencies

	All repeats/Best hit* (Low Stringency)		No repeats/Best hit* (Medium Stringency)		No repeats/Unique hit** (High Stringency)	
	DW7	SD20	DW7	SD20	DW7	SD20
PE alignment (M)	75.59	56.87	22.18	44.10	21.65	36.40
SE alignment (M)	5.80	9.11	0.52	3.43	0.41	2.20
Unmapped (M)	2.92	17.26	6.72	19.56	7.36	28.49
Paired read count (M)	14.71	33.54				
**Total read count (M)**	**29.42**	**67.09**				

*Best hit = up to 4 mismatches; **Unique = no mismatches; Mapping performed with SOAP2
