# Supplementary material for: Comparative Genomics of a Plant-Pathogenic Fungus, Pyrenophora tritici-repentis, Reveals Transduplication and the Impact of Repeat Elements on Pathogenicity and Population Divergence
Source: G3 (Bethesda). 2013 Jan 1;3(1):41–63. doi: 10.1534/g3.112.004044 (PMC3538342; doi:10.1534/g3.112.004044)
Supplement: Supporting Information [file supp_3.1.41_TableS6.pdf]

Table S6 EST libraries produced to facilitate gene calling

Libraries	Pathogenicity	Conditions	Reads	Spans	Average Sequence Identity %
Pt-1C(BFP)	Yes-Race 1	In planta	10,083	3,088	
Pt-1C(BFP)	Yes-Race 1	Mixed culture	10,444	12,198	
SO3-P	Yes-Race 9	Mixed culture	10,185	12,115	98.08
SD20-NP	No-Race 4	Mixed culture	10,328	8,195	98.2
