# Supplementary material for: Comparative Genomics of a Plant-Pathogenic Fungus, Pyrenophora tritici-repentis, Reveals Transduplication and the Impact of Repeat Elements on Pathogenicity and Population Divergence
Source: G3 (Bethesda). 2013 Jan 1;3(1):41–63. doi: 10.1534/g3.112.004044 (PMC3538342; doi:10.1534/g3.112.004044)
Supplement: Supporting Information [file supp_3.1.41_TableS7.pdf]

Table S7 Serine codon usage in *P. tritici-repentis*

Ser codon	% usage	# of codons
AGC	19.4	118705
AGT	12.4	76150
TCA	18.4	112959
TCC	16.4	100858
TCG	16.8	103107
TCT	16.6	101583
**Total**	**100**	**613362**
