# Supplementary material for: Comparative Genomics of a Plant-Pathogenic Fungus, Pyrenophora tritici-repentis, Reveals Transduplication and the Impact of Repeat Elements on Pathogenicity and Population Divergence
Source: G3 (Bethesda). 2013 Jan 1;3(1):41–63. doi: 10.1534/g3.112.004044 (PMC3538342; doi:10.1534/g3.112.004044)
Supplement: Supporting Information [file supp_3.1.41_TableS8.pdf]

Table S8 Top five repeat familes shared between the *P. tritici-repentis* reference genome and resequenced pathogenic and non-pathogenic isolates

	Reference family	Repeat class	Annotation*
Pathogenic - DW7-ToxB			
	8	DNA transposon	*hAT* superfamily; similar to Tfo1 from *F. oxysporum*
	63	DNA transposon	*hAT* superfamily; similar to Restless
	105	DNA transposon	*Tc1/Mariner* superfamily
	49	DNA transposon	*Tc1/Mariner* superfamily; similar to Molly from *S. nodorum*
	619	DNA transposon	*hAT* superfamily; similar to Restless
Non-pathogenic-SD20-NP			
	26	Non-LTR retrotransposon	similar to Tad1 of *Blumeria*
	27	Non-LTR retrotransposon	similar to Tad1 of *Blumeria*
	238	Non-LTR retrotransposon	similar to Tad1 of *Blumeria*
	116	unknown	
	293	unknown	

*Repeat annotation performed in Censor
