# Supplementary material for: Comparative Genomics of a Plant-Pathogenic Fungus, Pyrenophora tritici-repentis, Reveals Transduplication and the Impact of Repeat Elements on Pathogenicity and Population Divergence
Source: G3 (Bethesda). 2013 Jan 1;3(1):41–63. doi: 10.1534/g3.112.004044 (PMC3538342; doi:10.1534/g3.112.004044)
Supplement: Supporting Information [file supp_3.1.41_TableS9.pdf]

Table S9 Count of identical 16-mers in 1X sampling of resequencing reads of DW7-ToxB and SD20-NP

	DW7-ToxB		SD20-NP	
Times present	Count	% of Total	Count	% of Total
1 to 10	21571304	88.03	22331413	94.66
11 to 20	1585463	6.47	534634	2.27
21 to 30	523010	2.13	309650	1.31
31 to 40	251216	1.03	250797	1.06
41 to 50	113859	0.46	102102	0.43
51 to 60	24937	0.10	41720	0.18
61 to 70	7766	0.03	9912	0.04
71 to 80	3857	0.02	3513	0.01
81 to 90	6792	0.03	1368	0.01
91 to 100	4498	0.02	382	0.00
101 to 110	2746	0.01	631	0.00
111 to 120	1566	0.01	737	0.00
121 to 130	2732	0.01	280	0.00
131 to 140	6719	0.03	0	0.00
141 to 150	11469	0.05	378	0.00
151 to 160	11769	0.05	2237	0.01
161 to 170	4064	0.02	900	0.00
171 to 180	4337	0.02	0	0.00
181 to 190	5051	0.02	0	0.00
191 to 200	4506	0.02	0	0.00
201 to 210	4314	0.02	0	0.00
211 to 220	3851	0.02	0	0.00
221 to 230	8033	0.03	0	0.00
231 to 240	12958	0.05	0	0.00
241 to 250	16405	0.07	0	0.00
251 to 260	11951	0.05	0	0.00
261 to 270	11559	0.05	0	0.00
271 to 280	9348	0.04	0	0.00
281 to 290	14204	0.06	0	0.00
291 to 300	18206	0.07	0	0.00
301 to 310	21485	0.09	0	0.00
311 to 320	36139	0.15	0	0.00
321 to 330	35316	0.14	0	0.00
331 to 340	38744	0.16	0	0.00
341 to 350	32057	0.13	0	0.00
351 to 360	23219	0.09	0	0.00
361 to 370	10710	0.04	0	0.00
371 to 380	10067	0.04	0	0.00

381 to 390	11319	0.05	0	0.00
391 to 400	10130	0.04	0	0.00
401 to 410	9831	0.04	0	0.00
411 to 417	6554	0.03	0	0.00
**Total**	**24504061**		**23590654**	
