# Supplementary material for: Comparative Genomics of a Plant-Pathogenic Fungus, Pyrenophora tritici-repentis, Reveals Transduplication and the Impact of Repeat Elements on Pathogenicity and Population Divergence
Source: G3 (Bethesda). 2013 Jan 1;3(1):41–63. doi: 10.1534/g3.112.004044 (PMC3538342; doi:10.1534/g3.112.004044)
Supplement: Supporting Information [file supp_3.1.41_TableS12.pdf]

Table S12 Pfam domains in repeats*

PFAM description	# in repeats
AMP-binding enzyme	6
AT hook motif	26
Bacterial regulatory helix-turn-helix proteins, AraC family	2
Bacterial regulatory proteins, lacI family	2
Bacterial SH3 domain	2
Centromere binding protein B, DNA binding	3
'chromo' (CHRromatin Organisation MOdifier) domain	32
Condensation domain	6
Core histone H2A/H2B/H3/H4	33
DDE superfamily endonuclease	111
hAT family dimerisation domain	178
helix-turn-helix, Psq domain	72
Integrase core domain	157
LTXXQ motif	14
MORN repeat variant	5
Motilin/ghrelin	7
Opioid growth factor receptor repeat	2
Osmosensory transporter coiled coil	23
Phosphopantetheine attachment site	8
PPAK motif	1
Protein of unknown function (DUF1299)	1
Protein of unknown function (DUF1409)	4
Retrotransposon gag protein	4
Reverse transcriptase (RNA-dependent DNA polymerase)	139
RNase H	33
SAMP Motif	1
Transposase	1
Viral A-type inclusion protein repeat	16
WCCH motif	19
WD domain, G-beta repeat	1
Zinc knuckle	28

*Pfam domains predicted by Broad Institute and present in custom repeat library
