# Supplementary material for: Comparative Genomics of a Plant-Pathogenic Fungus, Pyrenophora tritici-repentis, Reveals Transduplication and the Impact of Repeat Elements on Pathogenicity and Population Divergence
Source: G3 (Bethesda). 2013 Jan 1;3(1):41–63. doi: 10.1534/g3.112.004044 (PMC3538342; doi:10.1534/g3.112.004044)
Supplement: Supporting Information [file supp_3.1.41_TableS13.pdf]

Table S13 ***De novo* assembly of resequenced P. tritici-repentis isolates***

	DW7-ToxB	SD20-NP
k-mer	41	41
# contigs	21300	40383
largest contig (nt)	225936	320323
L50 (nt)	39556	69524
% contigs > 10 kb	85	88
% nt in contigs > 500 nt	98	98

* Assembly performed in Velvet
