# Supplementary material for: Comparative Genomics of a Plant-Pathogenic Fungus, Pyrenophora tritici-repentis, Reveals Transduplication and the Impact of Repeat Elements on Pathogenicity and Population Divergence
Source: G3 (Bethesda). 2013 Jan 1;3(1):41–63. doi: 10.1534/g3.112.004044 (PMC3538342; doi:10.1534/g3.112.004044)
Supplement: Supporting Information [file supp_3.1.41_TableS15.pdf]

Table S15 Putative NRPS-containing clusters in the *P. tritici-repentis* reference genome

P. tritici-repentis locus	Size (aa)	Putative function
PTRG_04244		
PTRG_04241	461	p450
PTRG_04242	410	flavoprotein monooxygenase
PTRG_04243	322	2OG-Fe(II) oxygenase
PTRG_04244	3850	pks:nrps hybrid
PTRG_04245	558	FAD-linked oxidase
PTRG_04246	491	p450 monooxigenase
PTRG_04247	384	predicted protein
PTRG_04248	452	alcohol oxidase
PTRG_04249	407	oxidoreductase
PTRG_04250	169	hypothetical protein
PTRG_04251	557	MFS efflux pump transporter
PTRG_04252	297	putative short chain dehydrogenase
PTRG_04253	401	O-methyltransferase
PTRG_04254	540	FAD-linked oxidase
PTRG_00447		
PTRG_00447	1282	nrps10
PTRG_00448	182	
PTRG_00449	199	
PTRG_00450	317	cyclin d-interacting protein
PTRG_00451	295	TBP-binding protein, activator of basal transricption
PTRG_00452	363	uncharacterized, conserved
PTRG_00453	347	
PTRG_00454	421	p450
PTRG_00455	317	unbiquitin ligase
PTRG_00456	296	zinc finger protein
PTRG_00457	554	AMP domain
PTRG_01683		
PTRG_01677	548	oxidoreductase
PTRG_01678	603	Long chain fatty acid acyl-CaA ligase
PTRG_01679	438	
PTRG_01680	423	Flavin-containing monooxygenase
PTRG_01681	1299	ABC transporter
PTRG_01682	219	N-acetyltransferase
PTRG_01683	1841	nrps6
PTRG_01684	475	
PTRG_01685	360	tyrosinase

PTRG_01800		
PTRG_01796	1270	splicing coactivator
PTRG_01797	539	MFS
PTRG_01798	89	
PTRG_01799	1489	ABC transporter
PTRG_01800	6909	nrps4
PTRG_01801	79	
PTRG_01802	369	Malic acid transporter
PTRG_01803	187	secreted protein
PTRG_01804	172	
PTRG_01805	507	p450
PTRG_01806	689	F-box domain
PTRG_01807	294	cyclin-like F-box domain
PTRG_08276		
PTRG_08272	858	translation initiation factor
PTRG_08273	906	YAKI-serine threonine kinase
PTRG_08274	529	Flavin-containing monooxygenase
PTRG_08275	1310	ABC transporter
PTRG_08276	5326	nrps2
PTRG_08280	402	Lipid phosphatase, PAP2 family
PTRG_08281	161	DNA-binding protein
PTRG_08282	476	mRNA splicing factor
PTRG_08283	276	transcription factor
PTRG_08284	1049	von Willebrands; may be two proteins or misannotated
PTRG_08285	743	AAA+-type ATPase
PTRG_08286	706	
PTRG_08287	855	RNA-binding protein
PTRG_08288	445	Hormone sensitive-lipase
PTRG_08289	165	
PTRG_08290	144	DNA methyltransferase
PTRG_08291	349	
PTRG_09101		
PTRG_09095	1432	ABC transporter
PTRG_09096	328	RTA1-like protein
PTRG_09097	391	Aminotransferase, class IV
PTRG_09098	2054	Fatty acid synthase, beta
PTRG_09099	1590	Beta-ketoacyl synthase
PTRG_09100	537	P450
PTRG_09101	6024	nrps
PTRG_09102	446	kynurenine 3-monooxygenase

PTRG_09103	552	MFS
PTRG_12015		
PTRG_12011	597	FAD-linked oxidoreductase
PTRG_12012	305	Short chain dehydrogenase
PTRG_12013	266	Short chain dehydrogenase
PTRG_12014	564	MFS transporter
PTRG_12015	4634	nrps HDAC
PTRG_12016	470	Basic leucine zipper transcription factor
PTRG_12017	242	Thioesterase
PTRG_12018	339	Aminotransferase
PTRG_12019	532	Cytochrome P450
PTRG_12020	1618	Fatty acid synthase
PTRG_12021	437	Cytochrome P450
PTRG_12022	338	Cytochrome P450
