# Supplementary material for: Comparative Genomics of a Plant-Pathogenic Fungus, Pyrenophora tritici-repentis, Reveals Transduplication and the Impact of Repeat Elements on Pathogenicity and Population Divergence
Source: G3 (Bethesda). 2013 Jan 1;3(1):41–63. doi: 10.1534/g3.112.004044 (PMC3538342; doi:10.1534/g3.112.004044)
Supplement: Supporting Information [file supp_3.1.41_TableS17.pdf]

Table S17 ***Pyrenophora tritici-repentis*-specific secreted proteins as predicted in Blast2GO**

Locus	Size (aa)	% Cys	HMM	E-value	Present DW7	Present SD20	EST			
							Race1 culture	Race1 Planta	Race4 culture	Race9 culture
Race 1 specific										
PTRG_05296	118	0.85			no	no				
PTRG_04919	123	0.82			no	no				
PTRG_10524	59	6.9			no	no				
Pathogen Specific										
PTRG_06853	122	1.65			yes	no				
PTRG_11888	86	14.12			yes	no	7	8		4
PTRG_12138	73	9.72			yes	no	14	2		
Discontinuously distributed										
PTRG_09431	73	4.17			no	yes				
Present in all sequenced isolates										
PTRG_00723	184	0.55			yes	yes				
PTRG_05020	85	0	GRP	0.0022	yes	yes				2
PTRG_10257	61	0			yes	yes	2		14	7
PTRG_00384	63	1.61			yes	yes				
PTRG_00529	52	0			yes	yes				
PTRG_00558	80	10.13			yes	yes		2		
PTRG_00758	75	4.05			yes	yes				
PTRG_00935	54	9.43			yes	yes				
PTRG_01132	87	2.33			yes	yes				
PTRG_01371	54	11.32			yes	yes		2		3
PTRG_01382	201	0			yes	yes				
PTRG_01617	59	0			yes	yes				
PTRG_01675	55	3.7			yes	yes				
PTRG_01823	133	6.06			yes	yes				
PTRG_01890	50	4.08			yes	yes				
PTRG_01923	78	7.79			yes	yes				
PTRG_02034	128	3.94			yes	yes				
PTRG_02202	90	2.25			yes	yes				
PTRG_02218	92	3.3			yes	yes				
PTRG_02241	99	6.12			yes	yes				
PTRG_02456	90	1.12			yes	yes				
PTRG_02606	91	5.56	PRA1	0.098	yes	yes				
			Innexin	0.036						
PTRG_02982	76	10.67			yes	yes				
PTRG_03151	69	1.47			yes	yes				
PTRG_03183	50	4.08			yes	yes	2	1		

PTRG_03424	129	6.25			yes	yes				
PTRG_03447	65	0			yes	yes				
PTRG_03460	79	2.56			yes	yes				
PTRG_03545	92	6.59			yes	yes				
PTRG_03740	137	0.74			yes	yes				
PTRG_03833	155	6.49			yes	yes				
PTRG_03972	117	3.45			yes	yes				
PTRG_04061	95	7.45			yes	yes				
PTRG_04438	132	3.05			yes	yes				
PTRG_04467	75	1.35			yes	yes				
PTRG_04476	130	3.88			yes	yes				
PTRG_04489	137	3.68			yes	yes				
PTRG_04511	57	5.36			yes	yes				
PTRG_04567	113	6.25	Peptidase_M19	0.076	yes	yes			2	2
			Meleagrin	0.089						
PTRG_04595	127	3.97			yes	yes				
PTRG_04645	109	3.7			yes	yes				
PTRG_04835	99	8.16			yes	yes			2	
PTRG_04870	73	9.72			yes	yes				
PTRG_05019	142	0			yes	yes				
PTRG_05021	143	1.41			yes	yes				
PTRG_05037	130	1.55			yes	yes				
PTRG_05069	95	6.38			yes	yes				
PTRG_05127	95	6.38			yes	yes				
PTRG_05187	141	0			yes	yes	8			2
PTRG_05203	130	0.78			yes	yes				2
PTRG_05392	96	3.16			yes	yes				
PTRG_05427	81	1.25	DEC-1_N	0.0014	yes	yes				
			PLRV_ORF5	0.048						
PTRG_05658	110	7.34			yes	yes				
PTRG_05788	395	0.51			yes	yes				2
PTRG_05858	77	0			yes	yes				
PTRG_05999	77	2.63			yes	yes				
PTRG_06015	56	1.82			yes	yes				
PTRG_06042	119	3.39			yes	yes				
PTRG_06295	57	0			yes	yes	2			
PTRG_06429	140	6.47			yes	yes		2		
PTRG_06505	295	0			yes	yes				
PTRG_06517	104	7.77			yes	yes				
PTRG_06556	94	3.23			yes	yes				
PTRG_06557	52	1.96			yes	yes				2
PTRG_06608	53	1.92			yes	yes				
PTRG_07001	69	8.82			yes	yes				

PTRG_07260	292	3.78			yes	yes				
PTRG_07271	88	5.75			yes	yes				
PTRG_07510	74	1.37			yes	yes				
PTRG_07515	128	7.09			yes	yes				
PTRG_07580	168	0	PIR	0.0000046	yes	yes	10	2	6	4
PTRG_07935	80	1.27	Peptidase_M66	0.0094	yes	yes				
PTRG_07936	164	3.68			yes	yes				
PTRG_08136	76	2.67	Mfp-3	0.032	yes	yes				
PTRG_08379	69	2.94			yes	yes	8		2	
PTRG_08398	107	1.89			yes	yes				
PTRG_08448	130	3.1			yes	yes				
PTRG_08572	87	16.28			yes	yes	2			
PTRG_08636	60	0			yes	yes				
PTRG_09109	92	6.59			yes	yes				
PTRG_09139	105	3.85			yes	yes	6			
PTRG_09257	293	1.71			yes	yes				
PTRG_09351	77	10.53			yes	yes				2
PTRG_09380	96	7.37			yes	yes	2		1	
PTRG_09458	49	4.17			yes	yes	2			
PTRG_09580	459	1.97			yes	yes				
PTRG_09958	97	6.25			yes	yes				
PTRG_10036	54	1.89			yes	yes				
PTRG_10069	70	4.35			yes	yes				
PTRG_10109	153	0.66			yes	yes	8		2	2
PTRG_10127	119	2.54			yes	yes	2			4
PTRG_10138	130	0			yes	yes				
PTRG_10852	61	5			yes	yes				
PTRG_10951	142	1.42			yes	yes				
PTRG_10990	123	3.28			yes	yes				
PTRG_11024	51	4			yes	yes				
PTRG_11036	135	4.48			yes	yes				
PTRG_11089	64	9.52			yes	yes	6	3		2
PTRG_11261	70	5.8			yes	yes				
PTRG_11351	94	0			yes	yes				
PTRG_11361	122	4.96			yes	yes				
PTRG_11416	60	1.69			yes	yes	2			
PTRG_11417	65	4.69	PsaX	0.05	yes	yes				
