# Supplementary material for: Comparative Genomics of a Plant-Pathogenic Fungus, Pyrenophora tritici-repentis, Reveals Transduplication and the Impact of Repeat Elements on Pathogenicity and Population Divergence
Source: G3 (Bethesda). 2013 Jan 1;3(1):41–63. doi: 10.1534/g3.112.004044 (PMC3538342; doi:10.1534/g3.112.004044)
Supplement: Supporting Information [file supp_3.1.41_TableS18.pdf]

Table S18 Total numbers of predicted CAZymes in *PTR* and selected ascomycetes

Species	GH	GT	PL	CE	CBM	EXPN
Pyrenophora tritici-repentis	245	91	10	39	45	2
Mycosphaerella graminicola	191	99	3	18	21	3
Trichoderma reesei	192	93	6	17	48	4
Fusarium graminearum	247	102	21	44	67	4
Neurospora crassa	173	76	4	22	42	1
Magnaporthe grisea	232	92	5	47	65	1
Aspergillus nidulans	251	91	21	31	41	1
Stagonospora nodorum	273	89	10	50	61	4

GH = glycoside hydrolases; GT = glycosyl transferases; PL = polysaccharide lyases; CE =carbohydrate esterases; CBM = carbohydrate binding modules; EXPN = distantly related to plant expansins. Predicted CAZymes were identified using the carbohydrate-active enzymes database tools
