# Supplementary material for: Comparative Genomics of a Plant-Pathogenic Fungus, Pyrenophora tritici-repentis, Reveals Transduplication and the Impact of Repeat Elements on Pathogenicity and Population Divergence
Source: G3 (Bethesda). 2013 Jan 1;3(1):41–63. doi: 10.1534/g3.112.004044 (PMC3538342; doi:10.1534/g3.112.004044)
Supplement: Supporting Information [file supp_3.1.41_TableS19.pdf]

Table S19 Comparison of pectinolytic enzymes in *P. tritici-repentis* vs. other grass and non-grass plant pathogens

Species	PL family							GH family					
	1	3	4	9	11	20	**Total**	28	78	88	95	105	**Total**
Magnaporthe grisea 70-15	2	1	1	0	0	1	**5**	3	1	1	1	3	**9**
Pyrenophora tritici-repentis Pt-1C-BFP	3	3	4	0	0	0	**10**	6	3	1	2	3	**15**
Phaeosphaeria nodorum SN15*	4	2	4	0	0	0	**10**	4	4	1	2	3	**14**
Gibberella zeae PH-1 (*F. graminearum*)	9	7	3	1	0	1	**21**	6	7	1	2	3	**19**
Gibberella moniliformis 7600 (*F. verticillioides*)	11	7	3	2	0	0	**23**	9	9	2	1	3	**24**
Fusarium oxysporum f. sp. lycopersici 4286	11	7	3	2	1	0	**24**	15	14	3	2	5	**39**
Verticillium albo-atrum VaMs.102	16	11	4	2	1	0	**34**	12	9	4	2	3	**30**
Verticillium dahliae VdLs.17	17	11	4	2	1	0	**35**	12	9	4	2	4	**31**

* Data from Amselem et al. 2011
