# Supplementary material for: Comparative Genomics of a Plant-Pathogenic Fungus, Pyrenophora tritici-repentis, Reveals Transduplication and the Impact of Repeat Elements on Pathogenicity and Population Divergence
Source: G3 (Bethesda). 2013 Jan 1;3(1):41–63. doi: 10.1534/g3.112.004044 (PMC3538342; doi:10.1534/g3.112.004044)
Supplement: Supporting Information [file supp_3.1.41_TableS20.pdf]

Table S20 GO enrichment analysis of ESTs present in the in planta library*

GO Term	Name	FDR	FWER	single test p-Value
GO:0003735	structural constituent of ribosome	2E-09	7.1E-09	7.9E-14
GO:0046933	hydrogen ion transporting ATP synthase activity, rotational mechanism	0.0003	0.002	0.000017
GO:0008553	hydrogen-exporting ATPase activity, phosphorylative mechanism	0.0048	0.047	0.00033
GO:0015935	small ribosomal subunit	0.0052	0.051	0.00037
GO:0045263	proton-transporting ATP synthase complex, coupling factor F(o)	0.0088	0.092	0.00058
GO:0043581	mycelium development	0.017	0.18	0.0013
GO:0031176	endo-1,4-beta-xylanase activity	0.02	0.29	0.0021
GO:0030245	cellulose catabolic process	0.02	0.29	0.0021
GO:0008810	cellulase activity	0.043	0.54	0.0035
GO:0004784	superoxide dismutase activity	0.043	0.54	0.0035
GO:0006414	translational elongation	0.043	0.54	0.0035
GO:0006801	superoxide metabolic process	0.053	0.65	0.0054
GO:0009279	cell outer membrane	0.075	0.8	0.0069
GO:0003871	5-methyltetrahydropteroyltriglutamate-homocysteine S-methyltransferase activity	0.075	0.8	0.0069
GO:0019843	rRNA binding	0.079	0.82	0.0078
GO:0005576	extracellular region	0.083	0.85	0.01
GO:0006099	tricarboxylic acid cycle	0.088	0.89	0.012
GO:0004775	succinate-CoA ligase (ADP-forming) activity	0.11	0.95	0.013
GO:0043022	ribosome binding	0.11	0.95	0.013

* Fisher's exact test performed in Blast2GO; Most specific terms
