# Supplementary material for: Comparative Genomics of a Plant-Pathogenic Fungus, Pyrenophora tritici-repentis, Reveals Transduplication and the Impact of Repeat Elements on Pathogenicity and Population Divergence
Source: G3 (Bethesda). 2013 Jan 1;3(1):41–63. doi: 10.1534/g3.112.004044 (PMC3538342; doi:10.1534/g3.112.004044)
Supplement: Supporting Information [file supp_3.1.41_TableS21.pdf]

Table S21 Comparison of CBM-containing protein families in *P. tritici-repentis* and other grass and non-grass plant pathogens

	CBM family																
Species	1	6	12	13	18	20	21	24	32	35	38	42	43	48	50	52	**Total**
Magnaporthe grisea 70-15	22	2	0	0	33	3	1	0	0	3	0	1	1	1	21	1	**89**
Pyrenophora tritici-repentis Pt-1C-BFP	11	1	0	0	19	2	1	0	1	3	0	1	2	3	0	0	**44**
Phaeosphaeria nodorum SN15 *	13	1	0	0	43	3	1	0	1	3	0	1	2	2	0	0	**70**
Gibberella zeae PH-1 (*F. graminearum*)	12	1	0	2	35	2	2	1	4	2	0	1	1	1	33	0	**97**
Gibberella moniliformis 7600 (*F. verticillioides*)	14	1	0	1	26	2	1	1	3	4	1	1	1	3	52	0	**111**
Fusarium oxysporum f. sp. lycopersici 4286	13	1	4	1	36	2	10	2	3	4	3	1	0	1	46	0	**127**
Verticillium albo-atrum VaMs.102	26	0	0	0	29	4	1	3	0	3	1	2	1	1	33	0	**104**
Verticillium dahliae VdLs.17	30	0	0	0	29	4	0	3	0	3	1	2	1	1	32	0	**106**

* Data from Anselem et al. 2011
